# Plasticity in ventral pallidal cholinergic neuron-derived circuits contributes to comorbid chronic pain-like and depression-like behaviour in male mice

**DOI:** 10.1038/s41467-023-37968-x

**Published:** 2023-04-17

**Authors:** Ya-Wei Ji, Zi-Lin Shen, Xue Zhang, Kairan Zhang, Tao Jia, Xiangying Xu, Huizhen Geng, Yu Han, Cui Yin, Jian-Jun Yang, Jun-Li Cao, Chunyi Zhou, Cheng Xiao

**Affiliations:** 1 Jiangsu Key Laboratory of Anesthesiology, Xuzhou Medical University, 221004 Xuzhou, China; 2 Jiangsu Province Key Laboratory of Anesthesia and Analgesia Application Technology, Xuzhou Medical University, 221004 Xuzhou, Jiangsu China; 3 NMPA Key Laboratory for Research and Evaluation of Narcotic and Psychotropic Drugs, School of Anesthesiology, Xuzhou Medical University, 221004 Xuzhou, Jiangsu China; 4 Department of Anesthesiology, Pain and Perioperative Medicine, The First Affiliated Hospital of Zhengzhou University, Zhengzhou, Henan China

**Keywords:** Neural circuits, Neuropathic pain, Depression

## Abstract

Nucleus- and cell-specific interrogation of individual basal forebrain (BF) cholinergic circuits is crucial for refining targets to treat comorbid chronic pain-like and depression-like behaviour. As the ventral pallidum (VP) in the BF regulates pain perception and emotions, we aim to address the role of VP-derived cholinergic circuits in hyperalgesia and depression-like behaviour in chronic pain mouse model. In male mice, VP cholinergic neurons innervate local non-cholinergic neurons and modulate downstream basolateral amygdala (BLA) neurons through nicotinic acetylcholine receptors. These cholinergic circuits are mobilized by pain-like stimuli and become hyperactive during persistent pain. Acute stimulation of VP cholinergic neurons and the VP-BLA cholinergic projection reduces pain threshold in naïve mice whereas inhibition of the circuits elevated pain threshold in pain-like states. Multi-day repetitive modulation of the VP-BLA cholinergic pathway regulates depression-like behaviour in persistent pain. Therefore, VP-derived cholinergic circuits are implicated in comorbid hyperalgesia and depression-like behaviour in chronic pain mouse model.

## Abstract (web summary)

The cholinergic circuits involved in pain modulation remain poorly understood. Here, the authors show that reversal of plasticity in the ventral pallidum-basolateral amygdala cholinergic pathway relieves hyperalgesia and depression-like behaviours in a chronic pain mouse model.

## Introduction

Chronic pain seriously affects patients’ quality of life and is a common causal factor in disabilities^1–4^, but its treatment is challenging because of central sensitization of the nervous system^5–7^. Another difficulty in the management of chronic pain is the comorbidity of emotional disorders, including depression and anxiety^8,9^. Neural circuits that separately or commonly modulate the sensory and emotional aspects of pain may be promising targets for treatment.

The cholinergic (ChAT) system shows promise for pain management. For instance, enhancing ChAT transmission by systemic administration of nicotinic acetylcholine (ACh) receptor (nAChR) agonists, acetylcholinesterase inhibitors, and ACh precursors mitigates inflammatory pain, migraine, and neuropathic pain^10–13^. However, blockade or down-regulation of nAChRs in the prefrontal cortex and basolateral amygdala (BLA) confers antidepressant-like effects^14–16^. Thus, although enhancement of the ChAT system confers analgesic effects, it may exacerbate comorbid depression in chronic pain. This paradoxical phenomenon may be due to the complexity of the ChAT system in the modulation of pain and emotion.

Accumulating studies have revealed that ChAT modulation of pain and emotion differ across brain regions and neuronal types, and the subtypes and subcellular locations of ACh receptors^14,15,17–21^. This diversity may be partially associated with the anatomical features of the endogenous ChAT system with discrete ChAT nuclei in the brain preferentially innervating particular brain regions^22–27^. For example, ChAT neurons in the basal forebrain project to several nuclei associated with nociception and emotion, including the prefrontal cortex, anterior cingulate cortex, somatosensory cortex, thalamic nuclei, hippocampus, basolateral amygdala (BLA), etc^25,26,28,29^. Ablation of basal forebrain ChAT neurons significantly blunts thermal responses^30^. Although inhibition of ChAT projections from the medial septum to the anterior cingulate cortex attenuates inflammatory pain responses in mice^23^, the neural circuit bases of the ChAT modulation of pain modalities and comorbid emotional disorders are far from being elucidated.

Some studies hint that the ventral pallidum (VP), a basal forebrain nucleus, may modulate pain and emotion. For instance, direct injection of a μ-opioid agonist in the VP in mice reduces ACh release in the BLA and prefrontal cortex and increases tolerance to thermal stimulation^28,31,32^, whereas stimulating glutamatergic inputs to the VP reduces mechanical and thermal pain thresholds^33^. In the VP, glutamatergic and GABAergic neurons play distinct roles in aversion processing and reward reinforcement learning, respectively; parvalbumin-positive neurons modulate depression-like behaviours^34–37^. Although the role of VP ChAT neurons in pain and emotion has not been investigated, their highly bifurcated collateral branches in the VP and their projections to the prefrontal cortex and amygdala^28^ suggest that they may regulate pain and emotion through downstream neurons.

In this work, we report that VP ChAT neurons innervate VP non-ChAT neurons and BLA neurons via nAChRs in mice; hyperactivity in these ChAT circuits contributes to pathophysiology in chronic pain states and is necessary and sufficient to confer hyperalgesia and depression-like behaviour in neuropathic pain mouse model. These results implicate VP-derived ChAT circuits in the modulation of both sensory and emotional aspects of pain.

## Results

### nAChRs in the VP regulate the mechanical withdrawal threshold

**Fig. 1:**
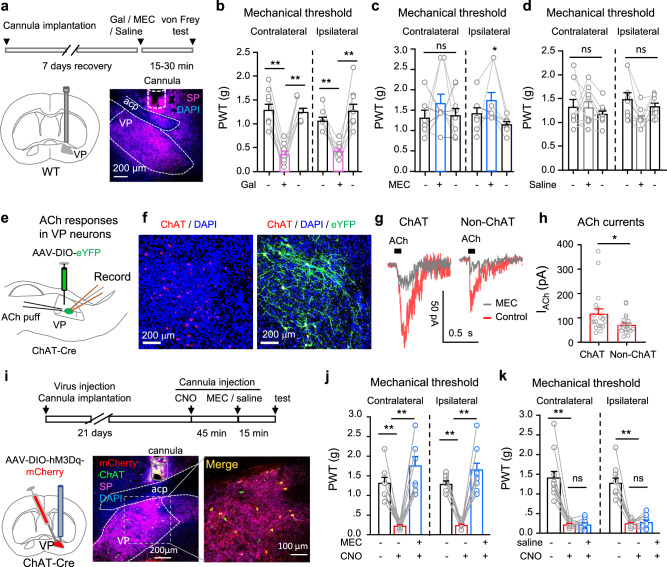
The VP ChAT circuit regulates the mechanical threshold. **a** Galantamine (Gal, 10 μM), mecamylamine (MEC, 10 μM), and saline were microinjected into the right VP through a cannula. A representative image of substance P (SP, purple) antibody-stained coronal brain section among nine repeats. **b**‒**d** Mechanical paw withdrawal thresholds (PWT) on both hind paws before (baseline), 15–30 min and 24 h after injection of Gal (**b**), MEC (**c**), and saline (**d**) into the VP (*n* = 9 mice). **b** Contralateral: *F*_(1.167, 9.338)_ = 29.45, *P* < 0.001; *t* = 6.83, df = 8, *P* < 0 001, Gal vs baseline. Ipsilateral: *F*_(1.626, 13.00)_ = 19.56, *P* < 0.001; *t* = 5.99, df = 8, *P* < 0 001, Ga1 vs baseline. **c** Contralateral: *F*_(1.895, 15.16)_ = 0.87, *P* = 0.44. Ipsilateral: *F*_(1.384, 11.07)_ = 5.48, *P* = 0.02; *t* = 3.31, df = 8, *P* = 0.01, MEC vs recovery. **d** Contralateral: *F*_(1.336, 10.69)_ = 0.80, *P* = 0.48. Ipsilateral: *F*_(1.620, 12.96)_ = 0.88. *P* = 0.45. **e** Whole-cell patch-clamp recordings were performed from virally labeled VP ChAT neurons to record responses to acetylcholine (ACh). **f** Representative images among five sets of repeats showing eYFP-labeled VP ChAT (ChAT) neurons. **g** ACh-evoked MEC-sensitive currents in VP ChAT and non-ChAT neurons. **h** Amplitude of ACh-evoked inward currents in ChAT (*n* = 19) and non-ChAT (*n* = 20) neurons in the VP from five mice. *t* = 2.12, df = 37, *P* = 0.04. **i** Schematic diagram for chemogenetic activation of VP ChAT neurons by delivering clozapine-N-oxide (CNO, 10 μM) into the VP through a cannula. Representative image is from 11 sections (one from each mouse) stained with ChAT (green) and SP (purple) antibodies and DAPI (blue). **j** Mechanical PWTs were measured before and 45–60 min after CNO or CNO-MEC was injected into the VP. *n* = 11 mice. **k** Mechanical PWTs were measured before and 45–60 min after CNO or CNO-saline was injected into the VP. *n* = 11 mice. One-way repeated-measures ANOVAs with Bonferroni tests and Geisser–Greenhouse correction were used in (**b**‒**d**) and (**j**, **k**). Two-tailed *t*-test was used in (**h**). *P < 0.05; **P < 0.01; ns not significant.

As inhibition of VP neurons relieves pain and stimulation of these neurons exacerbates pain^31–33^, we wondered whether enhancement of ChAT innervation in the VP would alter pain thresholds. We infused galantamine (Gal, 10 μM, 0.2 μl), an acetylcholinesterase inhibitor, to elevate the level of ACh in the right VP in mice (Fig. 1a), and observed a significant reduction in mechanical paw withdrawal threshold (PWT) in both left and right hind paws (Fig. 1b). However, neither mecamylamine (MEC, 10 μM, 0.2 μl), a nAChR antagonist, nor saline (0.2 μl) injection into the VP robustly altered PWT in the left or right hind paws (Fig. 1c, d). These data indicate that enhancement of ChAT transmission in the unilateral VP causes bilateral mechanical allodynia, but baseline ChAT innervation may not be crucial for the maintenance of normal mechanical thresholds.

To address whether ChAT and non-ChAT neurons in the VP mediate the effects of elevated ACh on the mechanical pain threshold, we labeled ChAT neurons in the VP in ChAT-Cre mice with a viral vector (AAV-EF1α-DIO-eYFP) (Fig. 1e, f). We then obtained whole-cell patch-clamp recordings from VP ChAT and non-ChAT neurons in brain slices to record the response to ACh puffed onto the somata^38–40^. We detected inward currents evoked by ACh puff (0.3 mM, 100 ms, 8 pSi) in 85% (28/33) of ChAT neurons and 83% (52/63) of non-ChAT neurons (Fig. 1g, h). The inhibition of the great majority of ACh responses by MEC (Fig. 1g) indicates that nAChRs primarily mediate ACh responses in VP neurons.

The axons of VP ChAT neurons have abundant bifurcations and form boutons with adjacent neurons in the VP^28^. This morphological feature suggests that VP ChAT neurons may innervate non-ChAT neurons in the VP. We next examined whether stimulation of VP ChAT neurons exerts effects on the mechanical threshold similar to galantamine, which causes accumulation of ACh released from VP ChAT neurons and ChAT inputs from other nuclei. In this set of experiments, we labeled VP ChAT neurons with AAV-EF1α-DIO-hM3Dq-mCherry, which allows selective activation of VP ChAT neurons with local injection of 10 μM clozapine-N-oxide (CNO) (Fig. 1i and Supplementary Fig. S1a–c). In Fig. 1j, k, we observed that infusion of CNO in the VP reduced the PWT; infusion of MEC, but not saline, into the VP 15 min earlier blocked VP CNO-infusion-induced mechanical allodynia in both hind paws (Fig. 1j, contralateral: *F*_(1.234, 12.34)_ = 21.73, *P* = 0.0003; *t* = 7.09, df = 10, *P* < 0.0001, baseline vs. CNO; *t* = 7.00, df = 10, *P* < 0.0001, CNO vs CNO + MEC. Ipsilateral: *F*_(1.391, 13.91)_ = 61.40, *P* < 0.0001; *t* = 13.24, df = 10, *P* < 0.0001, baseline vs. CNO; *t* = 8.85, df = 10, *P* < 0.0001, CNO vs CNO + MEC) (Fig. 1k, contralateral: *F*_(1.064, 10.64)_ = 42.77, *P* < 0.0001; *t* = 6.94, df = 10, *P* < 0.0001, baseline vs CNO; *t* = 0.04, df = 10, *P* = 0.97, CNO vs CNO + saline. Ipsilateral: *F*_(1.165, 11.65)_ = 56.22, *P* < 0.0001; *t* = 8.24, df = 10, *P* < 0.0001, baseline vs CNO; *t* = 0.73, df = 10, *P* = 0.48, CNO vs CNO + saline).

These results support the hypothesis that VP ChAT neurons are a source of ACh for the local ChAT circuit and modulate the mechanical threshold primarily through activation of nAChRs.

### Activation of VP ChAT neurons is involved in acute and persistent pain

**Fig. 2:**
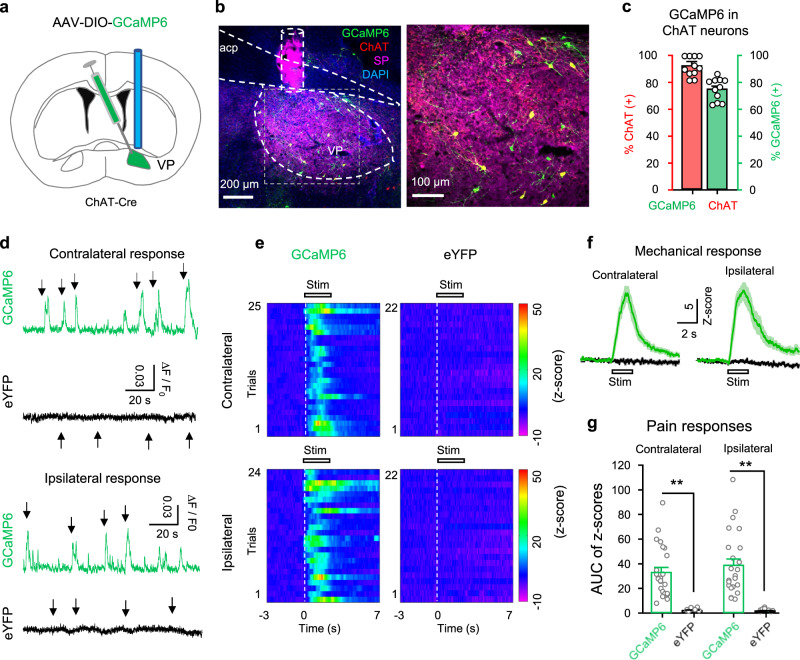
Enhanced activity in VP ChAT neurons upon acute pain-like stimulation. **a** AAV-EF1α-DIO-GCaMP6s was injected into the VP of ChAT-Cre mice. **b** A representative image of a coronal section containing GCaMP6 (green)-labeled neurons and ChAT- and SP-antibody-stained neurons and tissue. **c** Efficiency (green) (74 ± 3.20%, *n* = 12 section from six mice) and specificity (red) (93 ± 2.4%, *n* = 12 sections from six mice) of GCaMP6-labeling of ChAT neurons in the VP (*n* = 5 mice). **d** Representative raw traces showing the responses of GCaMP6-labeled (green) and eYFP-labeled (black) ChAT neurons in the VP to pain-like stimulation (pressure) on the contralateral and ipsilateral hind paws. Arrow, pain-like stimulation. **e** Heat maps showing the relative fluorescent signals from five GCaMP6 mice (*n* = 22 trials) and five eYFP mice (*n* = 25 trials) before (3 s), during (2 s), and after (7 s) the application of stimulation to the contralateral and ipsilateral hind paws. Each panel includes data from five mice (4–5 trials from each mouse). **f** The fluorescence intensities of GCaMP6 (green) and eYFP (black) were transformed into their z-scores (mean ± SEM). Each trace summarizes data from the same trials as this in (**e**). **g** The peak amplitudes of GCaMP6 (*n* = 22 trials from 5 GCaMP6 mice) and eYFP (*n* = 25 trials from five eYFP mice) responses to pain-like stimulation on hind paws. Contralateral: *t* = 7.91, df = 45, *P* < 0.001; ipsilateral: *t* = 7.38, df = 44, *P* < 0.001, two-tailed *t*-test. **P < 0.01.

To understand whether VP ChAT neurons respond to acute pain, we recorded fiber photometry signals in ChAT-Cre mice subjected to microinjection of AAV-EF1α-DIO-GCaMP6f or AAV-EF1α-DIO-eYFP and implantation of an optical fiber into the right VP (Fig. 2a–c). We observed that pressure (2 s, 200 g/mm^2^) on either left or right hind paw evoked a response in the VP of GCaMP6 mice, but not in eYFP mice (Fig. 2d, e). The peak amplitudes of the responses to stimulation on the left and right hind paws were comparable (Fig. 2f, g). These bilateral responses are consistent with our data showing that activation of the ChAT circuit in the right-hemisphere VP regulates the mechanical threshold on both sides (Fig. 1).

**Fig. 3:**
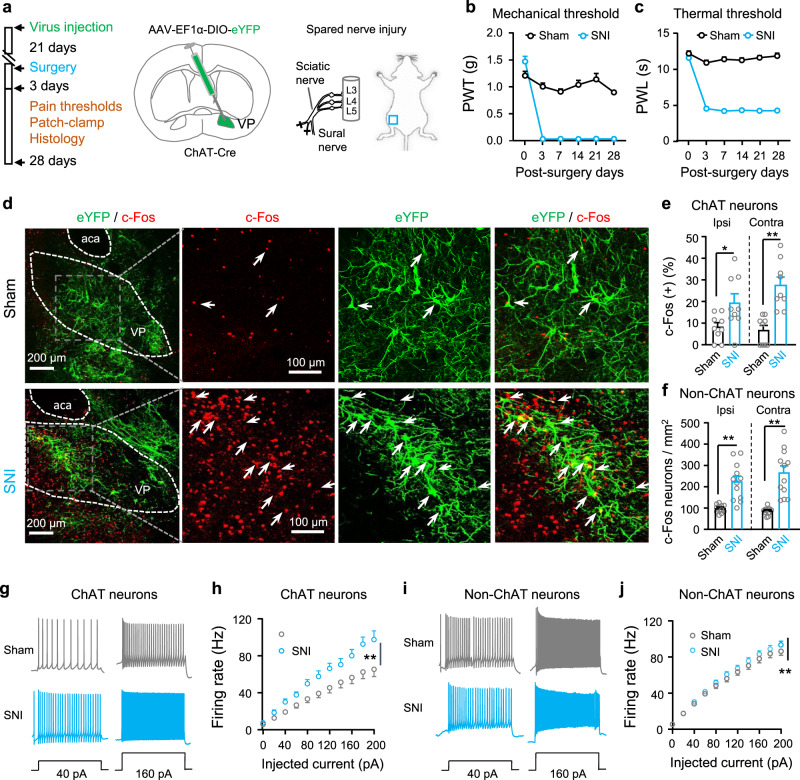
Mice with neuropathic pain exhibit hyperactivity in ChAT and non-ChAT neurons in the VP. **a** Schematic diagram for eYFP-labeling of VP ChAT neurons and for spared sciatic nerve injury (SNI) on the left side. **b** The mechanical threshold on the left hind paw was measured with von Frey filaments after surgery. Sham: *n* = 15 mice; SNI: *n* = 16 mice. Group: *F*_(1, 29)_ = 330.7, *P* < 0.0001. Time: *F*_(5, 145)_ = 75.51, *P* < 0.0001. **c** The thermal threshold on the left hind paw was measured with a plantar anesthesia tester after surgery. Sham: *n* = 15 mice; SNI: *n* = 16 mice. Group, *F*_(1, 29)_ = 688.2, *P* < 0.0001. Time, *F*_(5, 145)_ = 86.09, *P* < 0.0001. **d**–**f** Representative images (from contralateral VP) and summary showing c-Fos-positive neurons (red) in ChAT neurons (eYFP-labeled, green) and non-ChAT neurons (red only) in the VP on both sides. Sham: *n* = 9 sections from five mice; SNI: *n* = 9 sections from five mice. **e** Ipsilateral: *t* = 2.44, df = 16, *P* = 0.026; contralateral: *t* = 4.90, df = 16, *P* = 0.0002. **f** Ipsilateral: *t* = 4.96, df = 22, *P* < 0.0001; contralateral: *t* = 5.65, df = 22, *P* < 0.0001. **g**, **h** Representative traces and summary of firing rates in response to depolarizing current injections in ChAT neurons (*n* = 22 neurons from five Sham mice and *n* = 12 neurons from five SNI mice). Current: *F*_(10, 231)_ = 34.49, *P* < 0.0001, Group, *F*_(1, 121)_ = 113.2, *P* < 0.0001. **i**, **j** Representative traces and summary of firing rates in response to depolarizing current injections in non-ChAT neurons (*n* = 41 neurons from five Sham mice and *n* = 50 neurons from five SNI mice). Current: *F*_(10, 539)_ = 150, *P* < 0.0001; Group, *F*_(1, 440)_ = 7.75, *P* = 0.006. Two-way repeated-measures ANOVAs were used in (**b**, **c**, **h**, **j**). Two-tailed *t*-test was used in (**e**, **f**). **P < 0.01; ns not significant. Ipsi ipsilateral, Contra contralateral.

As chemogenetic stimulation of VP ChAT neurons led to hypersensitivity to mechanical stimulation (Fig. 1i–k), we hypothesized that VP ChAT neurons may be hyperactive in persistent pain states. To test this hypothesis, we established unilateral spared nerve injury (SNI) mouse model of neuropathic pain. The SNI mice exhibited a dramatic reduction in mechanical and thermal pain thresholds on the ipsilateral hind paw within 3 days, which lasted for more than 4 weeks (Fig. 3a–c). We sacrificed a group of SNI mice 10–15 days after surgery and observed a robust increase in c-Fos-positive neurons bilaterally in the VP (Fig. 3d), including ChAT neurons and non-ChAT neurons (Fig. 3e, f). To confirm that VP ChAT neurons are hyperactive in SNI mice, we performed patch-clamp recordings from virally labeled ChAT neurons and unlabeled non-ChAT neurons in sham and SNI mice 10–15 days after surgery (Fig. 3g, i). We observed that rates of firing evoked by depolarizing currents were significantly enhanced in both ChAT and non-ChAT neurons from SNI mice, relative to those in sham mice (Fig. 3g–j).

To address whether the release of ACh in the VP is elevated in SNI mice relative to sham mice, we microinjected AAV-hSyn-GACh3.0 into the VP to transfect VP neurons with an ACh sensor (GACh)^41,42^ (Supplementary Fig. S2a, b). We found that electrical-stimulation-triggered ACh release in VP slices (Supplementary Fig. S2c) was enhanced in SNI mice relative to sham mice (Supplementary Fig. S2d–f).

We wondered whether the difference in excitability of non-ChAT neurons in the VP between sham and SNI mice is caused by hyperactivity of ChAT neurons. To solve this puzzle, we recorded spontaneous firing from VP non-ChAT neurons in brain slices before and during MEC application. We observed that perfusion of 10 μM MEC inhibited most non-ChAT neurons in the VP in both sham and SNI mice, with a greater effect in SNI mice (Supplementary Fig. S3a–d). The enhanced spontaneous firing of non-ChAT neurons in SNI mice was normalized by MEC (Supplementary Fig. S3e). Furthermore, MEC also normalized depolarizing current-induced firing of non-ChAT neurons in SNI mice (Supplementary Fig. S3f–h). These data suggest that in the VP of SNI mice, the hyperactivity in non-ChAT neurons may be associated with higher excitability in ChAT neurons.

Therefore, VP ChAT neurons are activated in both acute and persistent pain mouse model. In persistent pain mouse model, neuronal hyperactivity and facilitation of ACh release are two forms of ChAT enhancement in the VP.

### Photostimulation of VP ChAT neurons induces hyperalgesia and depression-like behaviours

**Fig. 4:**
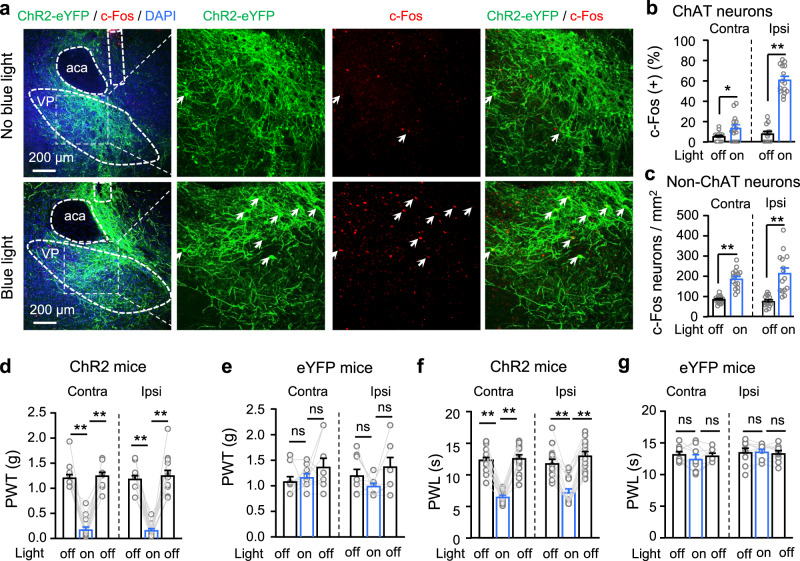
Optogenetic stimulation of VP ChAT neurons reduces mechanical and thermal thresholds and increases depression-like behaviours. **a**–**c** Representative images (ipsilateral side) and summary showing that unilateral optogenetic stimulation of VP ChAT (ChAT) neurons increased the number of c-Fos-positive ChAT neurons (red + green) and non-ChAT neurons (red only) in the VP on both sides. **b** ChAT neurons. Contralateral: *t* = 2.46, df = 28, *P* = 0.02. Ipsilateral: *t* = 12.99, df = 28, *P* < 0.0001. **c** non-ChAT neurons. Contralateral: *t* = 7.61, df = 28, *P* < 0.0001. Ipsilateral: *t* = 5.28, df = 28, *P* < 0.0001). No light: *n* = 15 sections; light: *n* = 15 sections. All from five mice in each group. **d**–**g** Mechanical PWT and thermal paw withdrawal latency (PWL) on hind paws before (off), during (on) (between 1–3 min light on), and after (off) (10 min after light off) illumination of the VP in ChR2 mice (*n* = 15) and eYFP mice (*n* = 9). **d** Contralateral: F_(1.817, 25.44)_ = 92.75, *P* < 0.001. *t* = 11.33, df = 14, *P* < 0.001, off vs on. Ipsilateral: *F*_(1.357, 18.99)_ = 71.08, *P* < 0.001. *t* = 15.92, df = 14, *P* < 0.001, off vs on. **e** eYFP mice. Contralateral: *F*_(1.422, 11.37)_ = 1.43, *P* = 0.27. Ipsilateral: *F*_(1.893, 15.14)_ = 3.01, *P* = 0.08. **f** Contralateral: *F*_(1.988, 27.83)_ = 94.43, *P* < 0.001. *t* = 12.01, df = 14, *P* < 0.001, off vs on. Ipsilateral: *F*_(1.723, 24.12)_ = 88.83, *P* < 0.001. *t* = 8.44, df = 14, *P* < 0.001, off vs on. **g** Contralateral: *F*_(1.738, 13.90)_ = 1.60, *P* = 0.24. Ipsilateral: *F*_(1.325, 10.60)_ = 0.17, *P* = 0.84. One-way repeated-measures ANOVAs with Bonferroni tests were used for (**d**–**g**). Two-tailed *t*-tests were used for (**b**, **c)**. **P < 0.01; ns not significant. Contra contralateral, Ipsi ipsilateral.

We next examined whether there is a causal link between the hyperactivity in VP ChAT neurons and hyperalgesia. To test this, we labeled VP ChAT neurons by microinjecting AAV-EF1α-DIO-ChR2-eYFP into the right VP in ChAT-Cre mice (Supplementary Fig. S1d, e). This strategy allows activation of VP ChAT neurons with high temporal resolution (Supplementary Fig. S1f). We observed that stimulation (light pulses, 5 ms, 20 Hz, 4 mW) (2 min episodes with 2 min intervals for 30 min) of ChAT neurons increased the number of c-Fos-positive ChAT neurons and non-ChAT neurons (Fig. 4a–c). Similar to our chemogenetic activation experiments (Fig. 1i–k), this acute optogenetic stimulation (light pulses, 5 ms, 20 Hz, 4 mW, 1–3 min) of VP ChAT neurons reversibly reduced mechanical PWT (Fig. 4d, e) and thermal paw withdrawal latency (PWL) (Fig. 4f, g) on both sides while the stimulation was present.

**Fig. 5:**
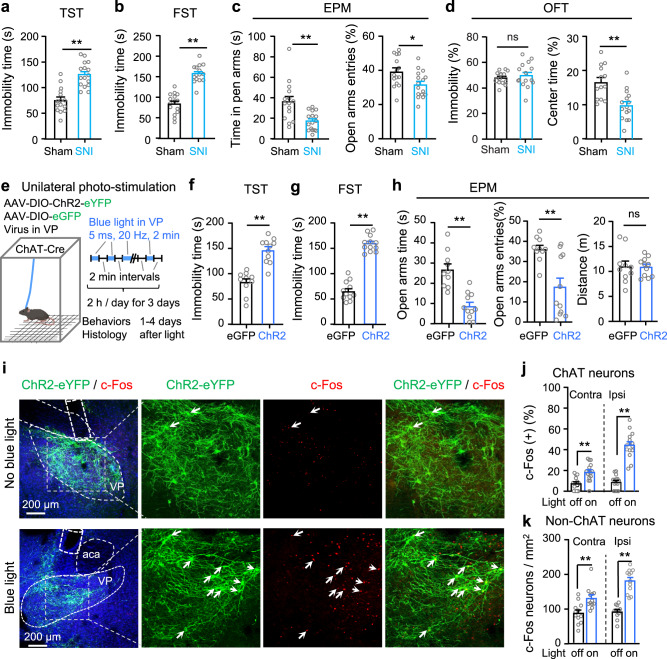
Repetitive optogenetic stimulation of VP ChAT neurons causes depression- and anxiety-like behaviours. **a**–**d** Tests for emotion-like behaviours were conducted in Sham mice (*n* = 13–15) and SNI mice (*n* = 15–20). **a** Tail suspension test (TST). *t* = 8.48, df = 28, *P* < 0.0001. **b** Forced swim test (FST). *t* = 5.79, df = 29, *P* < 0.0001. **c** Elevated plus maze (EPM). Time in open arms: *t* = 3.55, df = 29, *P* = 0.0013. Open-arm entries: *t* = 2.57, df = 29, *P* = 0.016. **d** Open field arena (OFT). Immobility: *t* = 0.73, df = 26, *P* = 0.47. Time in center: *t* = 3.88, df = 26, *P* = 0.0006. **e** Schematic diagram for repetitive (3-day) unilateral photo-stimulation of VP ChAT neurons in ChR2 and eGFP mice. **f** Immobility time in the TST. *t* = 6.13, df = 19, *P* < 0.0001. eGFP: *n* = 10; ChR2: *n* = 11. **g** Immobility time in the FST. *t* = 12.80, df = 21, *P* < 0.0001. eGFP: *n* = 11; ChR2: *n* = 12. **h** Time in and entries into the open arms of the EPM. Time in open-arms: *t* = 5.35, df = 19, *P* < 0.0001; Open-arm entries: *t* = 3.65, df = 19, *P* = 0.002; Distance in EPM: *t* = 0.13, df = 19, *P* = 0.90. eGFP: *n* = 10; ChR2: *n* = 11. **i**–**k** Representative images and summary showing c-Fos-positive ChAT and non-ChAT neurons in the VP in ChR2 mice 3–4 days after discontinuation of repetitive photo-stimulation of the VP. **j** ChAT neurons. Left: *t* = 4.04, df = 28, *P* = 0.0004. Right: *t* = 9.96, df = 28, *P* < 0.0001. *n* = 15 sections from five mice in each group. **k** Non-ChAT neurons. Left: *t* = 3.4, df = 22, *P* = 0.0026. Right: *t* = 7.93, df = 22, *P* < 0.0001. *n* = 12 sections from five mice in each group. Two-tailed *t*-tests were used for (**a**–**d**, **f**–**h**, **j**–**k**). **P < 0.01; ns not significant. Contra contralateral, Ipsi ipsilateral.

Consistent with our previous study^7^, we observed depression- and anxiety-like behaviours in SNI mice 4–6 weeks after surgery. SNI mice exhibited increased immobility time in the tail suspension test (TST) (Fig. 5a) and the forced swim test (FST) (Fig. 5b) than sham mice. Relative to sham mice, SNI mice showed less overall time spent in and number of entries to the open arms in the elevated plus maze (EPM) (Fig. 5c). In the open field test (OFT), SNI mice spent less time traveling through the central area of an open field arena without changes in immobility (Fig. 5d).

To understand whether hyperactive VP ChAT neurons are causally linked to these negative emotion-like behaviours in SNI mice, we applied a repetitive photo-stimulation paradigm to activate VP ChAT neurons in naive mice in vivo for 2 h each day on three consecutive days (Fig. 5e). To avoid heating effects of sustained light on the tissue, we applied 2 min episodes of 20 Hz blue light (5 ms pulses) with 2 min inter-episode intervals. We performed behavioural tests between 1–4 days after discontinuation of photo-stimulation, and then sacrificed mice for c-Fos-staining. This multi-day repetitive stimulation of VP ChAT neurons increased the number of c-Fos-positive ChAT neurons and non-ChAT neurons in the VP on both sides even 4 days after discontinuation of the stimulation (Fig. 5i–k). Accompanying the hyperactivity in the VP ChAT circuit were depression- and anxiety-like behaviours. Depression-like behaviour was represented by increased immobility time in the TST (Fig. 5f) and FST (Fig. 5g). Anxiety-like behaviour was manifested by reduced time in the open arms in the EPM (Fig. 5h). Note that, 3–4 days after discontinuation of the photo-stimulation, the mechanical and thermal thresholds and locomotion were not significantly different from those measured before multi-day repetitive photo-stimulation (Supplementary Fig. S4a–i). These data suggest that multi-day repetitive stimulation of VP ChAT neurons is sufficient to induce depression- and anxiety-like behaviours that persist after cessation of the stimulus, but not to alter pain thresholds.

These data suggest that persistent stimulation of VP ChAT neurons may be needed to reduce pain thresholds, whereas longer-term activation of ChAT neurons may be sufficient to induce depression- and anxiety-like behaviours.

### Inhibition of VP ChAT neurons relieves both sensation of pain-like stimuli and anxiety- and depression-like behaviours

**Fig. 6:**
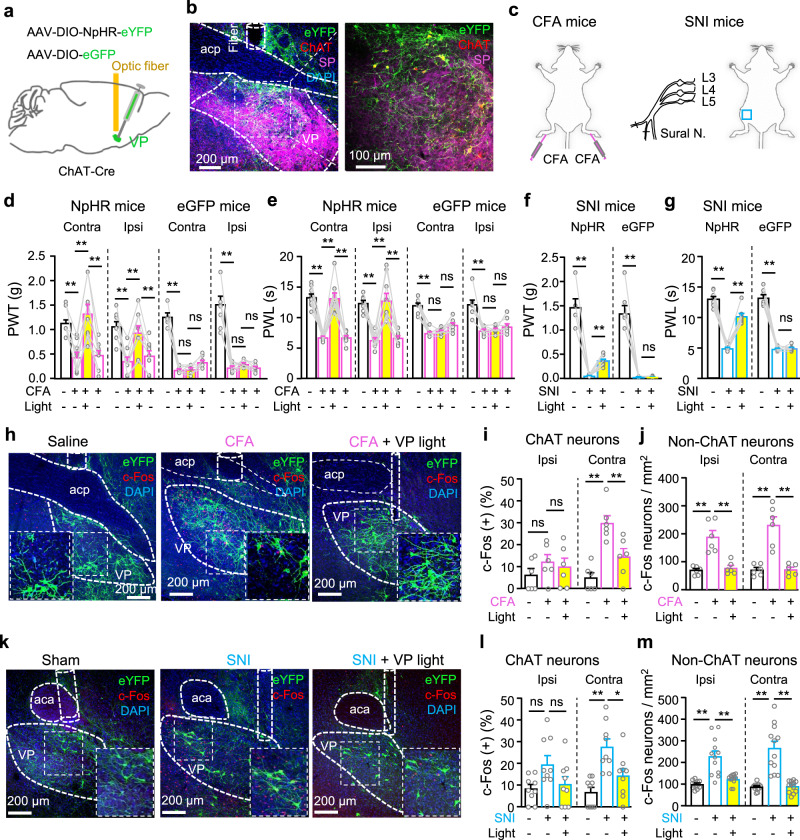
Optogenetic inhibition of VP ChAT neurons mitigates the reduction in pain thresholds in persistent pain. **a**, **b** Schematic diagram and a representative image out of 9 for optogenetic (NpHR) inhibition of VP ChAT neurons. **c** Establishment of pain mouse models. **d**, **e** PWT and PWL for both hind paws of NpHR (*n* = 9) and eGFP (*n* = 8) mice before, during, and after yellow-light illumination of the right VP, corresponding to before and 24, 48, 72 h after CFA injection. **f**, **g** PWT and PWL on the left hind paw of NpHR (*n* = 8) and eYFP (*n* = 7) mice before and during yellow light in the right VP in SNI mice. **f** NpHR: *F*_(1.060, 7.420)_ = 53.90, *P* = 0.0001; *t* = 8.24, df = 7, *P* = 0.0001, baseline vs SNI; *t* = 8.75, df = 7, *P* < 0.001, SNI vs SNI-VP-light. eYFP: *F*_(1.002, 6.010)_ = 67.52, *P* = 0.0002; *t* = 8.19, df = 6, *P* = 0.0004, baseline vs SNI; *t* = 0.39, df = 6, *P* = 0.71, SNI vs SNI-VP-light. **g** NpHR: *F*_(1.321, 9.245)_ = 104.3, *P* < 0.0001; *t* = 14.98, df = 7, *P* < 0.0001, baseline vs SNI; *t* = 14.14, df = 7, *P* < 0.001, SNI vs SNI-VP-light. eYFP: *F*_(1.224, 7.346)_ = 236.40, *P* < 0.0001; *t* = 14.97, df = 6, *P* < 0.0001, baseline vs SNI; *t* = 0.45, df = 6, *P* = 0.67, SNI vs SNI-VP-light. **h**–**j** Representative images and summary showing NpHR-labeled VP ChAT neurons and c-Fos-positive VP neurons from saline (*n* = 6), CFA (*n* = 6), and CFA-VP-light (*n* = 6) mice. **k**–**m** Representative images and summary showing NpHR-labeled VP ChAT neurons and c-Fos-positive ChAT and non-ChAT neurons in the VP from Sham (*n* = 9), SNI (n = 9), and SNI-VP-light (*n* = 9) mice. One-way repeated-measures ANOVAs with Bonferroni tests and Geisser–Greenhouse corrections were used for (**d**–**g**). One-way ANOVAs with Bonferroni tests were used for (**i**, **j**, **l**, **m**). *P < 0.05; **P < 0.01; ns not significant. Contra contralateral, Ipsi ipsilateral.

To examine whether reversing the hyperactivity in VP ChAT neurons effectively relieves chronic pain, we labeled VP ChAT neurons with AAV-EF1α-DIO-NpHR3.0-eYFP in ChAT-Cre mice (Fig. 6a, b and Supplementary Fig. S1g–h) so that we could inhibit them with constant yellow light (598 nm, 3 mW) (Supplementary Fig. S1i). We observed that, although acute optogenetic inhibition of VP ChAT neurons did not change PWT or PWL in naive mice (Supplementary Fig. S5a–d), it elevated the PWT and PWL on both sides in an acute inflammatory pain mouse model (Supplementary Fig. S5e–l) and a CFA-induced persistent inflammatory pain mouse model (Fig. 6c) (Fig. 6d, PWT. NpHR: contralateral, *F*_(1.952, 15.62)_ = 25.38, *P* < 0.001; *t* = 5.45, df = 8, *P* < 0.001, baseline vs CFA; *t* = 6.13, df = 8, *P* < 0.001, CFA vs CFA-VP-light; ipsilateral, *F*_(1.711, 13.68)_ = 12.69, *P* < 0.001; *t* = 4.74, df = 8, *P* = 0.003, baseline vs CFA; *t* = 8.20, df = 8, *P* = 0.001, CFA vs CFA-VP-light. eYFP: contralateral, *F*_(1.650, 11.55)_ = 141.5, *P* < 0.0001; *t* = 12.42, df = 7, *P* < 0.001, baseline vs CFA; *t* = 0.46, df = 7, *P* = 0.66, CFA vs CFA-VP-light; ipsilateral: *F*_(1.145, 8.014)_ = 60.80, *P* < 0.001; *t* = 7.64, df = 7, *P* = 0.0004, baseline vs CFA; *t* = 1.46, df = 7, *P* = 0.19, CFA vs CFA-VP-light) (Fig. 6e, PWL. NpHR: contralateral, *F*_(1.488, 11.91)_ = 51.60, *P* < 0.001; *t* = 11.90, df = 8, *P* < 0.001, baseline vs CFA; *t* = 8.62, df = 8, *P* < 0.001, CFA vs CFA-VP-light; ipsilateral, *F*_(1.277, 10.21)_ = 27.23, *P* < 0.001; *t* = 6.38, df = 8, *P* < 0.001, baseline vs CFA; *t* = 6.78, df = 8, *P* < 0.001, CFA vs CFA-VP-light. eYFP: contralateral, *F*_(1.980, 13.86)_ = 63.30, *P* < 0.001; *t* = 10.64, df = 7, *P* < 0.001, baseline vs CFA; *t* = 1.04, df = 7, *P* = 0.33, CFA vs CFA-VP-light; ipsilateral, *F*_(1.386, 9.705)_ = 20.72, *P* = 0.0006; *t* = 5.27, df = 7, *P* = 0.004, baseline vs CFA; *t* = 0.89, df = 7, *P* = 0.79, CFA vs CFA-VP-light). Acute optogenetic inhibition of VP ChAT neurons also elevated PWT and PWL on contralateral side in SNI mice (Supplementary Fig. S5m–o) (Fig. 6c, f, g). These results hint that the hyperactivity of VP ChAT neurons plays an important role in the maintenance of hypersensitivity to mechanical and thermal stimulation in both acute and persistent pain mouse models.

We conducted another set of experiments in which we established cohorts of CFA-induced persistent inflammatory pain mouse models and SNI mice. We observed that optogenetic inhibition of VP ChAT neurons on the side contralateral to CFA injection and SNI surgery significantly reduced the number of c-Fos-positive neurons in the VP on the photo-inhibited side (Fig. 6h, k). Combination of NpHR-eYFP-labeling of ChAT neurons and c-Fos-antibody-staining revealed that photo-inhibition of VP ChAT neurons reduced the number of c-Fos-positive ChAT and non-ChAT neurons on the photo-inhibited side by about 50% and 70%, respectively (Fig. 6i, ChAT neurons. Ipsilateral: *F*_(2, 15)_ = 0.79, *P* = 0.47. Contralateral: *F*_(2, 15)_ = 15.33, df = 15, *P* = 0.0002. *t* = 5.45, df = 15, *P* < 0.001, Saline vs CFA; *t* = 3.4, df = 15, *P* = 0.008, CFA vs CFA-VP-light) (Fig. 6j, non-ChAT neurons. Ipsilateral: *F*_(2, 15)_ = 21.40, *P* = 0.0004. Contralateral: *F*_(2, 15)_ = 25.83, *P* < 0.001) (Fig. 6l, ChAT neurons. Ipsilateral: *F*_(2, 24)_ = 3.22, *P* = 0.057. Contralateral: *F*_(2, 24)_ = 11.7, *P* = 0.0003) (Fig. 6m, non-ChAT neurons. Ipsilateral: *F*_(2, 33)_ = 19.52, *P* < 0.0001. Contralateral: *F*_(2, 33)_ = 29.71, *P* < 0.0001). Note that photo-inhibition also reduced the activity of non-ChAT neurons, but not ChAT neurons, on the side ipsilateral to CFA and SNI and contralateral to the photo-inhibition (Fig. 6i, j, l, m). These data suggest that the ChAT circuit in the VP, composed of both ChAT and non-ChAT neurons and governed by local ChAT neurons, is hyperactive in persistent pain states; inhibition of ChAT neurons is sufficient to normalize this circuit and mitigates the hypersensitivity to mechanical and thermal stimuli.

To clarify whether photoinhibition of VP ChAT neurons alleviates bothersome emotions caused by SNI, we performed conditioned place preference assay (Supplementary Fig. S6a–d). We observed that after 3 conditioning sessions, sham mice subjected to eYFP or NpHR transfection in VP ChAT neurons did not exhibit preference to photoinhibition-paired chamber (Supplementary Fig. S6e–g); in contrast, SNI mice with NpHR in VP ChAT neurons displayed preference to VP photoinhibition-paired chamber (Supplementary Fig. S6h–j). These data suggest that photoinhibition of VP ChAT neurons may confer positive emotions in SNI mice.

**Fig. 7:**
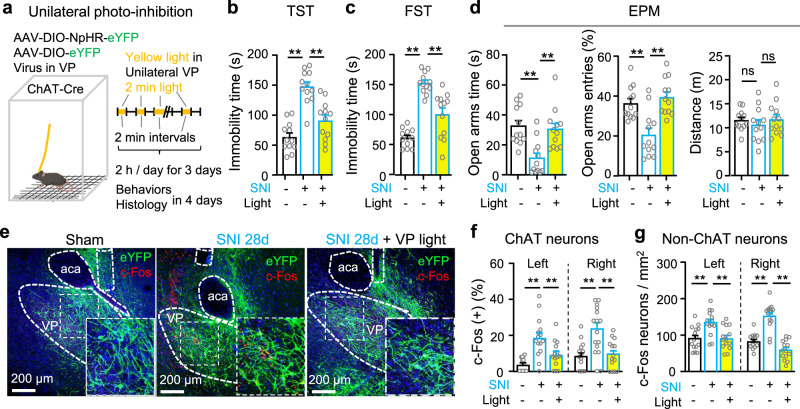
Repetitive photoinhibition of VP ChAT neurons mitigates depression- and anxiety-like behaviours in persistent pain. **a**–**d** Schematic diagram for repetitive optogenetic inhibition of VP ChAT neurons and behavioural tests in sham (*n* = 12), SNI (*n* = 12), SNI-VP-light (*n* = 12) mice. **b** TST. *F*_(2, 33)_ = 27.86, *P* < 0.0001. *t* = 7.32, df = 33, *P* < 0.0001, Sham vs SNI; *t* = 4.94, df = 33, *P* < 0.0001, SNI vs SNI-VP-light. **c** FST. *F*_(2, 33)_ = 39.43, df = 33, *P* < 0.0001. *t* = 8.85, df = 33, *P* < 0.0001, Sham vs SNI. *t* = 5.06, df = 33, *P* < 0.0001, SNI vs SNI-VP-light. **d** EPM. Time in open-arms: *F*_(2, 33)_ = 10.31, *P* = 0.0003. *t* = 4.12, df = 33, *P* = 0.0005, Sham vs SNI. *t* = 3.71, df = 33, *P* = 0.0008, SNI vs SNI-VP-light; Open-arm entries: *F*_(2, 33)_ = 12.95, *P* < 0.0001. *t* = 3.97, df = 33, *P* = 0.0004, Sham vs SNI; *t* = 4.74, df = 33, *P* < 0.0001, SNI vs SNI-VP-light; Distance: *F*_(2, 33)_ = 0.47, *P* = 0.63. **e**–**g** Representative images and summary of c-Fos(+) ChAT and non-ChAT neurons in sham (*n* = 15), SNI (*n* = 14), and SNI-VP-light (*n* = 14) sections from 5 mice in each group. **f** ChAT neurons. Left: *F*_(2, 41)_ = 13.51, *P* < 0.0001; *t* = 5.15, df = 41, *P* < 0.0001, sham vs SNI; *t* = 3.17, df = 41, *P* = 0.006, SNI vs SNI-VP-light. Right: F_(2, 41)_ = 12.73, *P* < 0.0001; *t* = 4.56, df = 41, *P* = 0.0001, sham vs SNI; *t* = 4.12, df = 41, *P* = 0.0004, SNI vs SNI-VP-light. **g** Non-ChAT neurons. Left: *F*_(2, 41)_ = 9.78, *P* = 0.0003; *t* = 3.89, df = 41, *P* = 0.001, sham vs SNI; *t* = 3.75, df = 41, *P* = 0.001, SNI vs SNI-VP-light. Right: *F*_(2, 41)_ = 44.84, *P* < 0.0001; *t* = 6.75, df = 41, *P* < 0.0001, sham vs SNI; *t* = 9.10, df = 41, *P* < 0.0001, SNI vs SNI-VP-light. One-way ANOVAs with Bonferroni tests were used for (**b**–**d**, **f**–**g**). * *P* < 0.05; **P < 0.01; ns not significant.

We then performed 3-day repetitive optogenetic inhibition [2 h yellow light stimulation (2 min episode of constant light with 2 min inter-episode intervals) per day for 3 days] of VP ChAT neurons in SNI mice 4 weeks after surgery and behavioural and histological assay between 1 and 4 days after discontinuation of optogenetic inhibition (Fig. 7a). We found that the multi-day repetitive photo-inhibition attenuated depression- and anxiety-like behaviours in SNI mice (Fig. 7b–d): causing a significant reduction in immobility time in TST and FST, and increasing time in the open arms in the EPM. By contrast, the 3-day photo-inhibition paradigm did not affect mechanical allodynia, thermal hypersensitivity, or movement in SNI mice, which were tested 3–4 days after discontinuation of photo-inhibition (Supplementary Fig. S5p–u). Furthermore, even 4 days after the discontinuation of the 3-day photo-inhibition paradigm, the number of c-Fos-positive ChAT and non-ChAT neurons remained significantly reduced (Fig. 7e–g). These results suggest that hyperactivity in VP ChAT circuit is crucial for the maintenance of hyperalgesia and depression- and anxiety-like behaviours in chronic pain mouse model.

To understand whether repetitive photo-inhibition of VP ChAT neurons mitigates anxiety- and depression-like behaviours with other etiology, we examined its effects on these behaviours in eYFP and NpHR mice subjected to either chronic restraint stress (CRS) or chronic unpredictable mild stress (CUMS) (Supplementary Fig. S7a–d). Chronically stressed mice showed anxiety- and depression-like behaviours in EPM, OFT, FST, and TST (Supplementary Fig. S7e–p) without activation of VP ChAT and non-ChAT neurons (Supplementary Fig. S8a–f). Repetitive yellow light illumination of the VP ChAT neurons did not reverse these behaviours (Supplementary Fig. S7e–p). These data suggest that VP ChAT neurons do not regulate anxiety- and depression-like behaviours following chronic stress.

### VP ChAT neurons modulate the activity of neurons in the basolateral amygdala

**Fig. 8:**
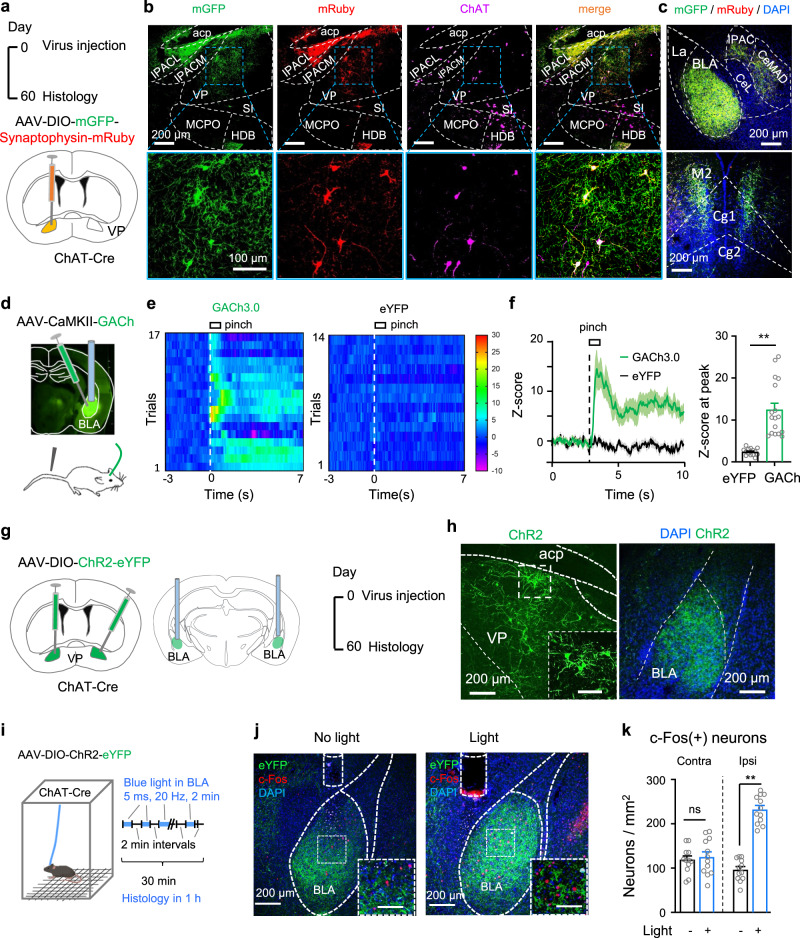
VP ChAT neurons innervate BLA neurons via functional connections. **a** AAV-EF1α-DIO-mGFP-Synpatophysin-mRuby was injected into the VP of ChAT-Cre mice. The mice were sacrificed 2 months later. **b** Histological verification of mGFP and mRuby in VP ChAT neurons. **c** mGFP and mRuby were colocalized in the BLA, the medial central amygdala (CeM), the cingulate cortex (Cg), and the secondary motor cortex (M2). **d** AAV-CaMKII-GACh, an ACh sensor, and AAV-hSyn-eYFP were transfected into the BLA in mice. Fiber photometry recordings were performed to monitor ACh release in the BLA. **e**, **f** GACh and eYFP signals in the BLA were transformed into z-scores. Heat-maps, summarized traces, and summary of the amplitudes of GACh and eYFP signals in response to a 1 s tail pinch are shown. Peak amplitudes: *t* = 5.683, df = 32, *P* < 0.0001, *n* = 17 trials from 6 GACh mice, *n* = 17 trials from six eYFP mice, two-tailed *t*-test. **g** AAV-EF1α-DIO-ChR2-eYFP was transfected in the VP of ChAT-Cre mice. **h** Representative images showing ChR2-expressing neurons in the VP and dense ChR2-expressing axonal fibers in the BLA. **i** AAV-EF1α-DIO-ChR2-eYFP was transfected into the right VP of ChAT-Cre mice and an optical fiber was implanted in the right BLA. Two-minute blue light stimulation (5 ms, 20 Hz, 4 mW) episodes with 2 min inter-episode intervals were delivered into the BLA for 30 min. The mice were sacrificed 1 h after stimulation for histological experiments. **j** Representative images showing that 30 min optogenetic stimulation of the VP^ChAT^-BLA projection increased the number of c-Fos-positive neurons in the BLA. **k** c-Fos-positive neurons in the BLA in both hemispheres in mice with and without light illumination. Contralteral: *t* = 0.47, df = 22, *P* = 0.64. Ipsilateral: *t* = 11.87, df = 22, *P* < 0.0001. *n* = 12 slices from five mice, two-tailed *t*-test. **P < 0.01; ns not significant. Contra contralateral, Ipsi ipsilateral. Scale bar in (**h**, **j**): 100 μm.

The preceding data demonstrate that VP ChAT neurons regulate both sensation of pain-like stimuli and associated anxiety- and depression-like behaviours. We wondered whether these neurons have connections with classical circuitry implicated in the transmission and modulation of pain signals. To address this question, we microinjected AAV-hSyn-FLEX-mGFP-T2A-Synaptophysin-mRuby into the VP of ChAT-Cre mice (Fig. 8a). This viral vector allows labeling somata and processes of Cre-positive neurons with mGFP and the terminals of these neurons with mRuby^43^ (Fig. 8b and Supplementary Fig. S9a). Therefore, the areas where both mGFP and mRuby reside are the major projection targets of VP ChAT neurons. We observed that the densest projections from VP ChAT neurons were in the BLA (Fig. 8c).

Above, we demonstrated that pain stimulation enhances activity in VP ChAT neurons (Figs. 2 and 3). We reasoned that if these pain-like stimulation-activated neurons project to the BLA, pain-like stimulation should increase ACh release in the BLA. To test this, we microinjected AAV-CaMKII-GACh3.0^41,42^ into the BLA so that we could monitor ACh levels using fiber photometry (Fig. 8d and Supplementary Fig. S2g, h). We observed that tail pinch evoked an elevation of ACh in the BLA (Fig. 8e, f). These data support the hypothesis that pain-like-stimulation-evoked activation of VP ChAT neurons may lead to an increase in ACh release in the BLA. Furthermore, in vivo stimulation of the VP^ChAT^-BLA projection increased the number of c-Fos-positive neurons in the BLA (Fig. 8g–k).

These data indicate that stimulation of the VP^ChAT^-BLA projection modulates neuronal activity in the BLA.

### Stimulation of the VP^ChAT^-BLA projection reduces pain thresholds and induces depression-like behaviours

**Fig. 9:**
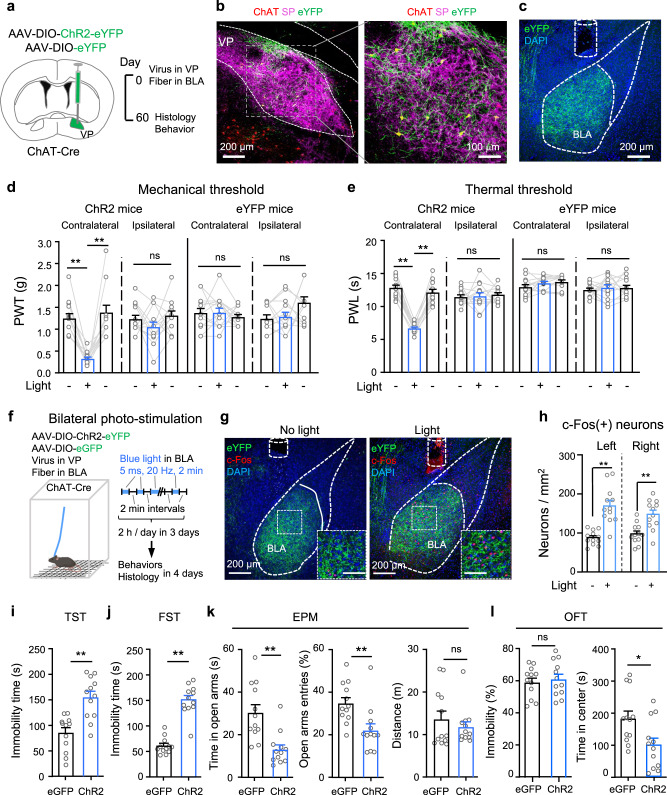
Optogenetic stimulation of the VP^ChAT^-BLA projection reduces pain thresholds and induces depression- and anxiety-like behaviours. **a**–**c** Schematic diagram and representative images for unilateral optogenetic stimulation of the VP-BLA ChAT projection. **d**, **e** Mechanical PWT and thermal PWL on hind paws before, during, and after unilateral BLA light in ChR2 (*n* = 13) and eYFP (*n* = 13) mice. **d** PWT. ChR2: contralateral: *F*_(1.555, 18.66)_ = 24.22, *P* < 0.001; *t* = 8.18, df = 12, *P* < 0.0001, off vs on; ipsilateral: *F*_(1.983, 23.80)_ = 1.79, *P* = 0.19. eYFP: contralateral, *F*_(1.993, 23.92)_ = 0.41, *P* = 0.67; ipsilateral, *F*_(1.432, 17.18)_ = 3.19, *P* = 0.08, off vs on. **e** PWL. Contralateral: *F*_(1.935, 23.22)_ = 128.1, *P* < 0.0001, *t* = 16.18, df = 12, *P* < 0.001, off vs on; ipsilateral: *F*_(1.631, 19.57)_ = 0.17, *P* = 0.80. eYFP: contralateral, *F*_(1.254, 15.04)_ = 1.81, *P* = 0.20; ipsilateral, *F*_(1.831, 21.97)_ = 0.27, *P* = 0.27. **f** Schematic diagram for 3-day repetitive bilateral stimulation of the VP^ChAT^-BLA projection. **g**, **h** Representative images (**g**) and summary (**h**) showing that repetitive photo-stimulation of the VP^ChAT^-BLA projection increased the number of c-Fos-positive neurons in the BLA, measured 2 days after discontinuation of photo-stimulation. Left: *t* = 5.58, df = 22, *P* < 0.0001; Right: *t* = 3.81, df = 22, *P* = 0.001. no light: *n* = 12 sections from seven mice; light: *n* = 12 sections from six mice. **i**–**l** Behavioural tests in ChR2 (*n* = 12) and eGFP (*n* = 12) mice. **i** TST. T = 4.46, df = 22, *P* = 0.0002. **j** FST. *t* = 9.31, df = 22, *P* < 0.0001. **k** EPM. Time in open-arms: *t* = 3.92, df = 22, *P* = 0.0007. Open-arms entries: t = 3.05, df = 22, *P* = 0.006. **l** OFT. Immobility: F = 0.45, df = 22, *P* = 0.66. Time in center: *t* = 2.57, df = 22, *P* = 0.017. One-way repeated-measures ANOVAs with Bonferroni tests and Geisser–Greenhouse corrections were used for (**d**, **e**). Two-tailed *t*-tests were used for (**h**–**l**) (left panel). Kurskal Wallis one-way ANOVA on ranks test was used for **l** (right panel). *P < 0.05; **P < 0.01; ns not significant.

To stimulate the VP^ChAT^-BLA projection, we microinjected AAV-EF1α-DIO-ChR2-eYFP into the VP and implanted an optical fiber in the right BLA of ChAT-Cre mice to stimulate the axon terminals (Fig. 9a–c and Supplementary Fig. S4j). Stimulation of the VP^ChAT^-BLA projection reduced mechanical and thermal thresholds on the contralateral side, but not on the ipsilateral side (Fig. 9d, e), unlike stimulation of VP ChAT somata, which reduced thresholds bilaterally (Fig. 4d, f). This result indicates that the VP^ChAT^-BLA projection mediates the effect of VP ChAT neurons on the pain threshold on the contralateral side, but not on the ipsilateral side. To confirm this result, we did another set of experiments in which we combined chemogenetic stimulation of VP ChAT neurons with optogenetic inhibition of the VP^ChAT^-BLA projection (Supplementary Fig. S9b–e). Chemogenetic stimulation of VP ChAT neurons alone reduced mechanical and thermal thresholds on both sides (Supplementary Fig. S9f, g); simultaneous photo-inhibition of the VP-BLA ChAT projection attenuated the effect of VP ChAT neurons on the contralateral hind paw, but not that on the ipsilateral hind paw (Supplementary Fig. S9f, g). Additionally, photo-inhibition of the VP-BLA ChAT projection significantly attenuated the activation of ipsilateral but not contralateral BLA neurons by chemogenetic stimulation of ipsilateral VP ChAT neurons (Supplementary Fig. S9h–j).

We next applied 3 days of repetitive bilateral photo-stimulation of the VP^ChAT^-BLA projection using the same protocol for repetitive stimulation of VP ChAT neurons in Fig. 5e (Fig. 9f). This manipulation resulted in hyperactivity in BLA neurons on both sides (Fig. 9g, h). We observed that 3-day photo-stimulation induced depression-like and anxiety-like behaviours: an increase in immobility time in the TST and FST and a reduction in open-arms visits in the EPM and time in center area in the OFT (Fig. 9i–l). However, repetitive photo-stimulation of the VP-BLA projection did not alter locomotion and mechanical and thermal thresholds (Fig. 9l and Supplementary Fig. S4k–m). These results suggest that bilateral repetitive stimulation of the VP^ChAT^-BLA projection is sufficient to induce depression-like behaviours in mice.

### Inhibition of the VP^ChAT^-BLA projection mitigates chronic pain

We transfected AAV-CaMKII-GACh3.0 into BLA neurons to monitor ACh levels in the BLA in brain slices in vitro (Supplementary Fig. S2g–i). We found that electrical stimulation induced greater ACh release in the BLA in SNI mice than in sham mice (Supplementary Fig. S2j–l). This result suggests that the ChAT inputs to the BLA are enhanced in persistent pain mouse model.

We next performed in vivo optrode single-unit recordings (Supplementary Fig. S10a, b) to address how optogenetic stimulation of VP ChAT inputs modulates BLA neurons. We found that spike rates of BLA neurons before and during photostimulation of the VP-BLA ChAT projection were significantly higher in SNI mice than sham mice (Supplementary Fig. S10c). Detailed analysis revealed that photostimulation of the VP-BLA ChAT projection stimulated about half of BLA neurons, but inhibited or did not affect some other BLA neurons (Supplementary Fig. S10d, e). The proportions of these effects were similar between sham and SNI mice (Supplementary Fig. S10e). Interestingly, photostimulation of the VP-BLA ChAT projection had a stronger excitatory effect, but a similar inhibitory effect, on spike rate of single-units in the BLA in SNI mice relative to sham mice (Supplementary Fig. S10f–i). These data suggest that in SNI, the excitatory effect of the VP-BLA ChAT projection is augmented.

We also labeled BLA projecting ChAT neurons with ChAT-antibody and fluorogold tracing from the BLA (Supplementary Fig. S11a–c). We observed that these ChAT neurons scarcely overlapped with CaMKII(+) neurons (mostly glutamatergic neurons) (Supplementary Fig. S11c, d) and 1/6 of these neurons overlapped with GAD67(+) neurons (GABAergic neurons) (Supplementary Fig. S11e–g). These data are consistent with previous study showing that ChAT, glutamatergic, and GABAergic neurons are largely separated in the VP^28,44^. It suggests that the excitatory effect of the VP-BLA ChAT inputs on BLA neurons may be mainly mediated by ACh release.

**Fig. 10:**
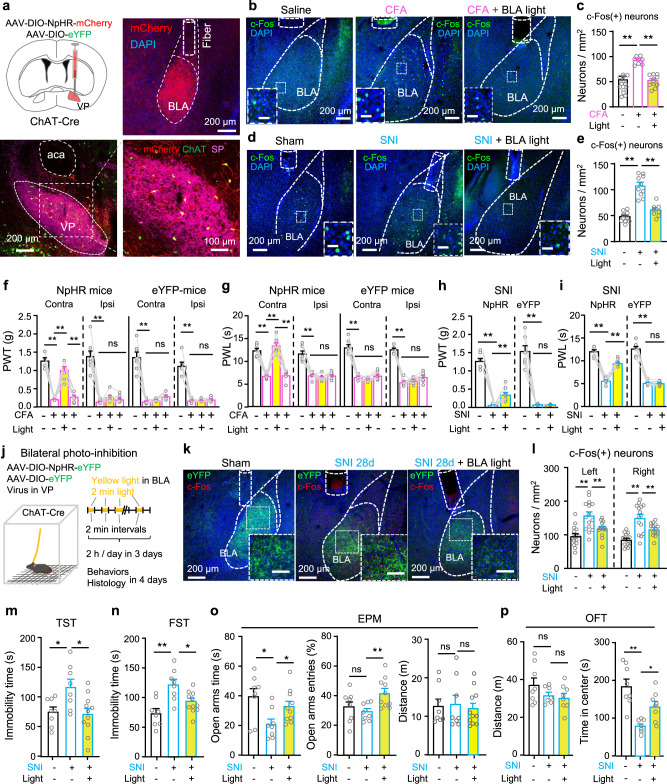
Optogenetic inhibition of the VP^ChAT^-BLA projection mitigates hyperalgesia and depression- and anxiety-like behaviours in persistent pain. **a** Schematic and representative images for unilateral optogenetic inhibition of the VP-BLA ChAT projection. **b**, **c** Representative images and summary of numbers of c-Fos(+) BLA neurons in Saline (*n* = 11), CFA (*n* = 8), CFA-BLA-light (*n* = 11) mice. *F*_(2, 27)_ = 41.18, *P* < 0.0001; *t* = 8.68, df = 27, *P* < 0.0001, saline vs CFA; *t* = 7.19, df = 27, *P* < 0.0001, CFA vs CFA-BLA-light. **d**, **e** Representative images and summary of numbers of c-Fos(+) BLA neurons in Sham (*n* = 10), SNI (*n* = 10), SNI-BLA-light (*n* = 8) mice. *F*_(2, 25)_ = 44.24, *P* < 0.0001; *t* = 9.00, df = 25, *P* < 0.0001, sham vs SNI; *t* = 6.65, df = 25, *P* < 0.0001, SNI vs SNI + BLA light. **f**, **g** Mechanical and thermal thresholds on both hind paws of NpHR and eYFP mice were measured before, during, and after illumination of the right VP^ChAT^-BLA projection, corresponding to before and 24, 48, and 72 h after CFA injection. eYFP-CFA: *n* = 7 mice; NpHR-CFA: *n* = 8 mice. **h**, **i** Mechanical and thermal thresholds on the left hind paw of NpHR (*n* = 8) and eYFP (*n* = 8) mice were measured before and during yellow light illumination of the right VP^ChAT^-BLA projection, 10 days after SNI surgery on the left side. **j** Schematic diagram showing 3-day bilateral optogenetic inhibition of the VP-BLA ChAT projection in sham and SNI mice. **k,** **l** Representative images and summary showing the numbers of c-Fos(+) BLA neurons in Sham, SNI, and SNI-BLA-light mice. *n* = 15 sections from five mice in each group. **m**–**p** Depression-like behaviours in eGFP-Sham (*n* = 8), SNI (*n* = 8), and SNI-BLA-light (*n* = 11) mice. One-way ANOVAs with Bonferroni tests were used in (**c**, **e**, **l**–**p**). Friedman Repeated-Measures ANOVA on Ranks was used in (**f**). One-way repeated-measures ANOVAs was used in (**g**–**i**). *P < 0.05; **P < 0.01; ns not significant. Scale bars in insets: 50 μm in (**b**, **d**); 100 μm in (**k**).

To address whether reversing the enhancement of ACh release and excitatory effect of the VP^ChAT^-BLA projection alleviates persistent pain-like behaviour, we labeled VP ChAT neurons specifically with AAV-EF1α-DIO-NpHR3.0-mCherry or AAV-EF1α-DIO-eYFP and implanted optical fibers in the BLA in ChAT-Cre mice (Fig. 10a and Supplementary Fig. S12a). We observed that photo-inhibition (589 nm laser, 3 mW, 2 min constant light with 2 min intervals for 30 min) of the VP^ChAT^-BLA projection (Supplementary Fig. S12b) was sufficient to reduce the number of c-Fos-positive BLA neurons in CFA and SNI mice to the levels in saline and sham mice (Fig. 10b–e). This is consistent with a previous study showing that inhibition of basal forebrain ChAT neurons compromises activity in BLA neurons^45^. Similar to inhibition of VP ChAT neurons (Supplementary Fig. S5a–d), photo-inhibition (589 nm, 3 mW, 1–3 min constant light) of the VP^ChAT^-BLA projection did not affect baseline mechanical and thermal thresholds in naive mice, but significantly increased the PWT in mice with capsaicin-induced acute inflammatory pain (Supplementary Fig. S12c–j), and elevated both PWT and PWL in CFA-induced persistent inflammatory pain and SNI neuropathic pain mouse models (Fig. 10f–i) (Fig. 10f, CFA, PWT. NpHR-CFA: contralateral, Chi-square = 19.95, *P* < 0.001, *q* = 2.91, *P* = 0.02, on vs off; ipsilateral, Chi-square = 19.63, *P* < 0.001; *q* = 3.15, *P* = 0.12. eYFP-CFA: contralateral, Chi-square = 17.91, *P* < 0.001, *q* = 1.17, *P* = 0.84, off vs on; ipsilateral, Chi-square = 13.48, *P* = 0.004, *q* = 0.59, *P* = 0.98, off vs on) (Fig. 10g, CFA, PWL. NpHR-CFA: contrateral, F_(2.532, 17.72)_ = 73.02, *P* < 0.001, *t* = 11.43, df = 7, *P* < 0.001, on vs off; ipsilateral, *F*_(1.549, 10.84)_ = 58.32, *P* < 0.001, *t* = 1.06, df = 7, *P* = 1.00, on vs off. eYFP-CFA: contralateral, *F*_(1.697, 10.18)_ = 115.10, *P* < 0.001, *t* = 1.15, df = 6, *P* = 1.00, on vs off; ipsilateral, *F*_(2.359, 14.15)_ = 141.49, *P* < 0.001, *t* = 0.79, df = 6, *P* = 1.00, off vs on) (Fig. 10h, SNI, PWT. NpHR-SNI: *F*_(1.502, 10.51)_ = 127.80, *P* < 0.001; *t* = 3.59, df = 7, *P* = 0.009, on vs off; eYFP: *F*_(1.007, 7.049)_ = 96.78, *P* < 0.001; *t* = 0.08, df = 7, *P* = 1.00, on vs off) (Fig. 10i, SNI, PWL. NpHR: *F*_(1.754, 12.28)_ = 101.38, *P* < 0.001; *t* = 8.40, df = 7, *P* < 0.001; eYFP: *F*_(1.180, 8.261)_ = 170.34, *P* < 0.001; *t* = 0.07, df = 7, *P* = 1.00, on vs off). However, the effects on pain thresholds were restricted to the contralateral hind paw, consistent with our data showing that stimulation of the VP^ChAT^-BLA projection induced contralateral mechanical allodynia and thermal hypersensitivity (Fig. 9d, e). Therefore, we postulate that hyperactivity of the VP^ChAT^-BLA projection is an important pathophysiology in persistent pain state.

We next examined whether 3-day repetitive inhibition of the VP^ChAT^-BLA projection counteracts hyperalgesia and depression- and anxiety-like behaviours in chronic pain mouse model. We found that bilateral 3-day photoinhibition significantly reduced the number of c-Fos-positive neurons in the BLA on both sides in SNI mice (Fig. 10j–l) (Left: *F*_(2, 41)_ = 12.87, *P* < 0.0001; *t* = 3.08, df = 41, *P* = 0.008, SNI vs SNI-BLA-light. Right: *F*_(2, 41)_ = 18.48, *P* < 0.0001; *t* = 3.14, df = 41, *P* = 0.007, SNI vs SNI-BLA-light) and relieved depression- and anxiety-like behaviours in SNI mice (10m-p). For instance, immobility times in the TST (*F*_(2, 24)_ = 5.45, *P* = 0.01. *t* = 2.61, df = 24, *P* = 0.02, eGFP-Sham vs SNI; *t* = 3.11, df = 24, *P* = 0.01, SNI vs SNI-BLA-light) and FST (*F*_(2, 24)_ = 9.76, *P* = 0.0008. *t* = 4.38, df = 24, *P* = 0.0006, eGFP-Sham vs SNI. *t* = 2.87, df = 24, *P* = 0.02, SNI vs SNI-BLA-light) were reduced. Time in and number of entries into the open arms in the EPM (time in open arms: *F*_(2, 24)_ = 4.65, *P* = 0.02. Open-arm entries: *F*_(2, 24)_ = 6.32, *P* = 0.01. Distance: *F*_(2, 24)_ = 0.10, *P* = 0.91) and time in the central area of the open field arena (Distance: *F*_(2, 20)_ = 1.77, *P* = 0.20. Time in center: *F*_(2, 20)_ = 13.06, *P* = 0.0002; *t* = 5.11, df = 20, *P* < 0.001, sham vs SNI; *t* = 2.53, df = 20, *P* = 0.02, SNI vs SNI-BLA-light) were prolonged (Fig. 8m–p). Similar to repetitive stimulation of the VP^ChAT^-BLA projection, repetitive inhibition of the VP^ChAT^-BLA projection did not alter pain thresholds and immobility in SNI mice (Supplementary Fig. S12k–p). However, it increased distance in central area in open field arena (Supplementary Fig. S12p). These results suggest that bilateral inhibition of the VP^ChAT^-BLA projection mimics inhibition of VP ChAT neurons and improves pain-associated depression- and anxiety-like behaviours in SNI mice.

### The VP-BLA ChAT pathway and cortisol, corticosterone, and heart rate variability

As literature frequently reports alterations in serum concentration of stress hormone^46,47^ and heart rate variability^48,49^ in rodent depression models, we next examined whether the VP-BLA ChAT pathway regulates these parameters.

We did not observe difference in serum concentration of cortisol and corticosterone between sham and SNI mice (Supplementary Fig. S13a, b), but the VP-BLA ChAT pathway exerted complex effects on the level of cortisol and corticosterone. Multi-day repetitive stimulation of either VP ChAT neurons or the VP-BLA projection did not change serum concentration of cortisol, whereas repetitive stimulation of the VP-BLA ChAT projection, but not VP ChAT neurons, elevated the concentration of corticosterone (Supplementary Fig. S13c, d, g, h). Repetitive inhibition of VP ChAT neurons reduced serum concentration of cortisol, but increased serum concentration of corticosterone; repetitive inhibition of the VP-BLA ChAT projection did not change serum concentration of cortisol and corticosterone (Supplementary Fig. S13e, f, i, j).

We observed that 5 weeks after surgery, relative to sham mice, SNI mice remained unchanged in heart rate, intervals between peaks of P and R waves, and duration of QRS complex, but exhibited a reduced variation of heart rate, represented by the standard deviation of R-R intervals (SDRR) (Supplementary Fig. S14a–e). These results are consistent with previous studies reporting that the variation of heart rate becomes narrower in patients with chronic anxiety and depression^50,51^. Repetitive inhibition of VP ChAT neurons did not change heart rate, RR interval, QRS duration, and SDRR in SNI mice (Supplementary Fig. S14e).

These results hint that the VP-BLA ChAT pathway may regulate anxiety- and depression-like behaviours in SNI mice probably through mechanisms involved in the central nervous system, but probably not stress hormone and autonomic nervous system.

## Discussion

Basal forebrain ChAT neurons are implicated in the modulation of multiple behaviours, including learning and memory, reward, emotion, arousal and sleep, anesthesia, pain, etc.^14,21–23,52–55^. Most of these studies regard the basal forebrain as a single nucleus and overlook its anatomical and neurochemical heterogeneity. Using a cell-type-specific chemogenetic technique, Jiang et al. demonstrated the involvement of medial septal ChAT neurons in pain modulation^23^. In the present study, we employed a repertoire of cell- and projection-specific strategies to demonstrate the role of VP ChAT neurons in pain modulation. First, a large majority of VP ChAT and non-ChAT neurons expressed functional nAChRs. Second, VP ChAT neurons responded to nociceptive stimulation. Third, chemogenetic and optogenetic stimulation of VP ChAT neurons reduced pain thresholds. Fourth, in persistent pain models (CFA and SNI mice), reversing the hyperactivity in VP ChAT neurons relieved mechanical allodynia and thermal hypersensitivity. These data explicitly implicate VP ChAT neurons in the perception of the pain-like stimulation and modulation of pain-like response. Extending the finding that lesion of basal forebrain ChAT neurons blunts thermal sensation^30^, our results suggest that VP ChAT neurons are among the pro-nociceptive ChAT neurons of the basal forebrain.

ChAT neurons accounting for only around 10% of VP neurons possess highly bifurcated collateral branches within the VP^28,34,56^; however, whether they modulate non-ChAT neurons have not been confirmed. In the present study, we found that optogenetic manipulations of VP ChAT neurons regulated non-ChAT neurons: optogenetic stimulation of VP ChAT neurons activated both ChAT and non-ChAT neurons in the VP; in persistent pain mouse models, both ChAT and non-ChAT neurons were hyperactive and inhibition of ChAT neurons reduced the number of activated non-ChAT neurons. Additionally, hyperactivity of VP non-ChAT neurons in SNI mice was normalized to the level in sham mice by blocking nicotinic receptors. In pain-like behavioural tests, we also observed that chemogenetic stimulation of VP ChAT neurons caused mechanical allodynia, and blockade of nAChRs in the VP eliminated this effect. These data demonstrate that the connections between ChAT and non-ChAT neurons in the VP are implicated in modulation of pain-like responses. Moreover, in mouse models of neuropathic pain, ACh release in the VP was exaggerated; inhibition of VP ChAT neurons mitigated hyperalgesia. These results support the hypothesis that enhancement of the ChAT circuit in the VP may affect non-ChAT neurons and contribute to central sensitization in neuropathic pain.

In addition to the modulation of pain thresholds, VP ChAT neurons are also implicated in regulation of anxiety- and depression-like behaviours in neuropathic pain mouse model. We observed that repetitive stimulation of VP ChAT neurons induced anxiety- and depression-like behaviours in naive mice; repetitive inhibition of VP ChAT neurons caused conditioned place preference and mitigated anxiety- and depression-like behaviours in SNI mice. Surprisingly, repetitive inhibition of VP ChAT neurons did not alleviate anxiety- and depression-like behaviours in chronic stress mouse models. These data suggest that VP ChAT neurons may regulate anxiety- and depression-like behaviours with particular etiology, such as neuropathic pain, instead of chronic stress.

VP ChAT neurons provide major ChAT innervation of the BLA^28,57,58^. Consistent with this, we demonstrated that VP ChAT neurons send dense projections to the BLA and regulate BLA neurons; furthermore, these projections modulated pain-like response. Interestingly, the VP-BLA ChAT projection regulated ipsilateral BLA neurons and mechanical and thermal thresholds on the contralateral hind paws, thereby differing from the bilateral actions of VP ChAT neurons. The bilateral effects of VP ChAT neurons in response to nociceptive stimulation and in pain modulation is similar to VP responses reported in previous studies^33,59^, whereas the unilateralism of the VP-BLA projection in pain modulation is consistent with the role of the amygdala in nociception^60,61^. Moreover, we observed that blocking the VP-BLA ChAT projection on one side eliminated the reduction in mechanical and thermal thresholds produced by unilateral stimulation of VP ChAT neurons, but only on the contralateral side, leaving the reduction on the ipsilateral side intact. From these data, we postulate that VP ChAT neurons affect VP and BLA neurons on both sides, whereas the VP-BLA ChAT projection affects the BLA on the ipsilateral side only; as a result, VP ChAT neurons and the VP-BLA ChAT projection regulate pain thresholds bilaterally and on the contralateral side, respectively. Chaves-Coira et al. reported that ChAT neurons in the diagonal band of Broca and substantia innominata project to cortical areas in both hemisphere but that those in the nucleus basalis magnocellularis project to ipsilateral cortical areas^62^. Further investigations are needed to address whether VP ChAT neurons affect contralateral VP neurons through cortical circuits.

The present study demonstrate that acute inhibition of either the VP ChAT circuit or the VP-BLA projection alleviates not only mechanical and thermal hypersensitivity, but also anxiety- and depression-like behaviours in neuropathic pain. In contrast to previous studies revealing that systemic administration of ChAT drugs exerts paradoxical effects on pain and depression- and anxiety-like behaviours^14,20^, our data hint that inhibition of VP ChAT neurons and the VP-BLA ChAT projection provide potential strategies to treat these symptoms. Furthermore, we identified different modulation patterns that conferred either analgesic- or anxiolytic-/antidepressant-like effects. Multi-day, repetitive inhibition of either VP ChAT neurons or the VP-BLA projection in SNI mice produced effects that outlasted the period of photo-inhibition, including attenuation of the hyperactivity of VP and BLA neurons and improvements in anxiety- and depression-like behaviours. On the contrary, multi-day repetitive stimulation of either VP ChAT neurons or their projections to the BLA led to hyperactivity in VP and BLA neurons, accompanied by anxiety- and depression-like behaviours. These results are consistent with previous findings showing that blocking or down-regulating nAChRs in the BLA induces anxiolytic- and antidepressant-like effects^15^. Although the long-lasting effects of multi-day repetitive stimulation or inhibition of the VP-BLA ChAT pathway effectively regulated anxiety- and depression-like behaviours, acute manipulation of VP neurons or the VP-BLA pathway was required to affect pain thresholds. These results suggest that repetitive and acute modulation of VP ChAT neurons and the VP-BLA projections may recruit different downstream circuits regulating pain thresholds and emotional states, respectively. Further investigations are needed to clarify this issue.

Therefore, in the VP, ChAT neurons regulate pain-, anxiety-, and depression-like behaviours through local ChAT circuits and the VP-BLA projection, whereas glutamatergic, GABAergic, and parvalbumin-positive neurons regulate aversion, reinforcement learning, arousal, and emotion, mainly via their projections to the lateral habenula, ventral tegmental area, and lateral hypothalamus^34–37,63^. Our results advance understanding of the physiology and anatomy of the VP.

There are several limitations of this study. First, we used optogenetic and chemogenetic technique to stimulate VP ChAT neurons and c-Fos staining to label the activated VP and BLA neurons. This method cannot clarify whether VP ChAT neurons directly and/or indirectly modulate VP non-ChAT neurons and BLA neurons. Sophisticated electrophysiology, receptor pharmacology, and molecular manipulations are required to address this mystery. Second, we found that VP ChAT neurons modulate non-ChAT neurons but, because of technique limitations, we did not address the exact role of these non-ChAT neurons in the modulation of pain-, anxiety-, and depression-like behaviours. Third, the results of this study were from male mice, and may not be extended to female mice. Fourth, we observed that there are a small proportion of BLA-projecting VP ChAT neurons overlapping with GABAergic neurons. Further investigations are needed to address the role of this group of ChAT neurons in modulation of pain-, anxiety-, and depression-like behaviours. Fifth, in this study, we only provide information about neuronal activity, ACh release, and behaviours. Biochemical and physiological bases linking these processes may warrant further investigations.

In summary, we described two pro-nociceptive circuits originating from VP ChAT neurons: one innervates non-ChAT neurons in the VP; the other projects to the BLA. Hyperactivity in these circuits may contribute to the pathophysiology of comorbid hyperalgesia-, anxiety-, and depression-like behaviours in persistent pain mouse models; disruption of the hyperactivity in different patterns may be required to mitigate these behaviours. Our data suggest that targeting VP ChAT-neuron-derived circuits may be a promising therapeutic strategy for the treatment of comorbid chronic pain and depression.

## Methods

### Animals

The care and use of animals and the experimental protocols (202011A363) used in this study were approved by the Institutional Animal Care and Use Committee and the Office of Laboratory Animal Resources of Xuzhou Medical University under the Regulations for the Administration of Affairs Concerning Experimental Animals (1988) in China. C57BL/6J background ChAT-IRES-Cre mice were purchased from the Jackson Laboratory (stock no. 006410). C57BL/6J mice were purchased from the animal facility of Xuzhou Medical University. The mice were group housed (no more than four per cage) on a 12-h light/dark cycle in an environment with stable temperature (21–23 °C) and humidity (40–70%). They have *ad libitum* access to water and food. Male C57BL/6J and heterozygous transgenic mice at least 8 weeks old were used for the experiments. All behavioural experiments were performed during the light cycle. Efforts were made to minimize animal suffering and to reduce the number of mice used.

### Viral vectors

The AAV serotype 2 viral vectors (AAV2) were used for neuronal tracing, neuromodulation, and ACh detection. AAV-EF1α-DIO-ChR2(H134R)-eYFP (Cat# AG20298), AAV-EF1α-DIO-NpHR3.0-eYFP (Cat# AG26966), AAV-EF1α-DIO-NpHR3.0-mCherry (Cat# H4882), AAV-EF1α-DIO-GCaMP6 (Cat# H10010), AAV-EF1α-DIO-eGFP (Cat# H3303), and AAV-EF1α-DIO-eYFP (Cat# AG20296) were purchased from OBIO Technology (Shanghai, China). The AAV2 viral vectors (1 × 10^12^ to 5 × 10^12^ vg/ml): AAV-EF1α-DIO-hM3Dq-mCherry (Cat# PT0019) was purchased from Brain VTA (Wuhan, China). AAV-hSyn-DIO-mGFP-T2A-synaptophysin-mRuby (Cat# BC-0316) was purchased from Brain Case (Shenzhen, China). AAV2-hSyn-GACh3.0 (YL001006) and AAV2-CaMKII-GACh3.0 (YL001003) were purchased from WZ Biosciences Inc. (Jinan, China). The titers of the viral vectors are between 1 × 10^12^ to 5 × 10^12^ vg/ml.

### Mouse survival surgeries

Male mice were anesthetized with an intraperitoneal injection of sodium pentobarbital (40 mg/kg) and stabilized in a stereotaxic apparatus (RWD Life Science Co., Shenzhen, China) with a heating pad. The surgery was performed as described previously^7,33,64,65^. After the mice were deeply anesthetized, confirmed by the loss of response to tail pinch, the fur on the head was shaved and the skin was thoroughly sterilized with 75% ethanol. More sodium pentobarbital (10–20 mg/kg) was administered if the mice responded to tail pinch. An incision from 3 mm before the Bregma to 3 mm after the Lamda was made with a scalpel and the skin was opened with two hemostats to widely expose the skull. The tissue on the skull was scraped and wiped away with cotton swabs to assure that the Bregma and Lamda were clearly discernible and the skull was rough. The level of the ear bars and the nose cone on the stereotaxic apparatus was adjusted to set the Bregma and Lamda on the same horizontal plane, and two corresponding points on left-right skull 1.5 mm away from the midline were on the same horizontal plane, as well. Then, a hole above the VP (AP, 0.5 mm; ML, 1.5 mm) or BLA (AP, −1.4 mm; ML, 3.1 mm) was drilled with a drill bit (0.5 mm in diameter, RWD Life Science Co., Shenzhen, China). These procedures allowed for the subsequent viral injections and optical fiber/cannula implantation.

Viral vectors (0.2 µl) or Fluorogold (2%, 0.1 µl) (KeyGEN BioTECH, Cat# KGMP023) were injected with a 10 μl Hamilton syringe (Timis County, Romania) driven by microinjection pump (KD Scientific, Holliston, MA, USA) at a rate of 50 nl/min. The coordinates for viral injection were as follows: VP (AP, 0.5 mm; ML, 1.5 mm; DV, 4.8 mm) and BLA (AP, −1.4 mm; ML, 3.1 mm; DV, 4.7 mm).

The optical fiber implants (200 μm in diameter, NA 0.37) (Inper, Hangzhou, China) were placed 200 µm above the injection site for optogenetic modulation, but at the injection site for fiber photometry recordings. The optical fiber implants were fixed to the skull with dental cement.

For pharmacological manipulation, stainless steel guide cannulas (26 G) (RWD Life Science, Shenzhen, China) were implanted 500 μm above the viral injection site in the VP as the needle (30 G) for drug delivery is 500 μm longer than the cannula.

For postoperative pain relief, meloxicam (4 mg/kg) was subcutaneously administered once per day for 3 days. The mice subjected to viral injection were allowed to recover for at least 3 weeks before the behavioural tests, optogenetic or pharmacological manipulations, electrophysiological recordings, and morphological assays. The mice subjected to cannula implantation were allowed to recover at least one week. The expression of viral vectors and the position of the optical fibers or cannula implants in mice were confirmed histologically after the experiments.

### Fiber photometry

A fiber photometry instrument (ThinkerTech, Nanjing, China)^66,67^ was used to monitor GCaMP6 signals in VP ChAT neurons and GACh signals in BLA neurons. We adjusted the instrument by setting the excitation light to 50 μW and the gain to a level that gave a background signal of 3 units measured when the end of the input cable was in the dark. After the input cable was connected to the optical implant in the mouse brain with a ceramic sleeve, the instrument read the total light signals. The difference between the total signal and the background signal was used as the baseline GCaMP6 signal. To evaluate responses in the VP and BLA to nociceptive stimuli, we designated the GCaMP6 or GACh signal during the 3 s just prior to the stimulation as the baseline value (F_0_); the peak responses in the GCaMP6 or GACh signal traces were quantified as [peak signal (F) − F_0_]/F_0_. To summarize the response, we calculated the mean and standard deviation (SD) of the GCaMP6 and GACh signal during the 3 s just prior to the stimulation and used these parameters to calculate the z-score ((F‒Mean)/SD) for each point in the GCaMP6 and GACh trace, thus transforming the GCaMP6 and GACh signal into a z-score trace. We then measured the area under the curve of the z-score plot to quantify the response to pain-like stimuli in the VP and BLA.

### Optogenetic manipulation

For optogenetic manipulations^65,68^, 473-nm and 598-nm laser generators (Newdoon, Hangzhou, China) were used to generate light to activate ChR2 and NpHR respectively. For optogenetic stimulation of ChR2-transfected neurons or their terminals, blue light (473 nm, 20 Hz, 4 mW) was delivered to the optical fiber implanted in the VP or BLA through a patch cable. The light was on for 30–120 s and the response to one trial of pain-like mechanical or thermal stimulation was examined within this time window. Two-minute blue light stimulation episodes were delivered with 2 min intervals for 30 min in c-Fos-immunostaining experiments and for 2 h in multi-day repetitive stimulation experiments. For optogenetic inhibition of NpHR-transfected neurons or their terminals, yellow light (589 nm, constant, 3 mW) was delivered with similar paradigm to blue light for examining the pain-like response, c-Fos-immunostaining, and multi-day repetitive inhibition.

### Pharmacological modulation

Mice with cannula implants in the VP were anesthetized with isoflurane and 0.2 μl saline with or without drugs was injected into the VP with a microinjector (KD Scientific, Holliston, MA, USA)^68^. Drugs, purchased from MedChemExpress, include galantamine (HY-76299, CAS: 357070-0), mecamylamine (HY-B1395A, CAS: 60-40-2), and clozapine-N-oxide (HY-17366, CAS: 34233-69-7). The drugs (10 μM galantamine, 10 μM mecamylamine, 10 μM clozapine-N-oxide) were individually loaded in a 10 μl Hamilton syringe (Timis County, Romania) which was connected to the injection needle with a tafflon tubing. When the mice were anesthetized with 1.5% isoflurane through a mask, the injection needle was inserted into the guide cannula and secured with a fixing screw. The syringe was driven by a microinjection pump (KD Scientific, Holliston, MA, USA) to inject 0.2 μl drug or saline into the VP at a rate of 50 nl/min. After injection, the needle was retained in place for 5 min before withdrawal to allow drug diffusion and then isoflurane was discontinued. The mice were allowed for 10–15 min to recover from anesthesia before behavioural tests.

In mice without cannula implants, clozapine-N-oxide (3 mg/kg) was intraperitoneally administered for chemogenetic modulation, and behavioural tests began 45 min after the injection.

### Establishment of pain-like mouse models

*Inflammatory pain mouse model* Capsaicin (0.01%, 20 µl in 10% DMSO/saline) (From MedChemExpress, HY-10448, CAS: 404-86-4) was injected in the lower hind limb and Complete Freund’s Adjuvant (CFA) (20 µl, from Sigma-Aldrich, F5581, CAS: 2098850-81-6) without dilution was injected into the plantar area of the hind paw.

*Neuropathic pain mouse model* Spared nerve injury (SNI) was performed according to previous reports^7,65^. Mice were anesthetized with an intraperitoneal injection of sodium pentobarbital (40 mg/kg), the fur in the operation area from the knee to the hip was shaved, and the skin was sterilized with 75% alcohol. A longitudinal incision was made. A blunt dissection was performed so that the biceps femoris muscle was separated to expose the sciatic nerve and its branches (sural, common peroneal, and tibial nerves). The common peroneal and tibial nerves were tightly ligated with a nylon suture and a section of the distal nerve bundle (2 mm) in each nerve was removed, but the sural nerve was kept intact (Figs. 3a and 5c). The muscle and skin incisions were subsequently sutured and mice were allowed to recover on a heating pad. Some mice were not subjected to nerve ligation and nerve severing. These mice were used as sham controls. Pain threshold in the skin area innervated by the sural nerve was measured.

### Chronic stress mouse models

Two chronic stress mouse models were established^69,70^. A group of mice were subjected to 4 h body restraint per day for 14 days (chronic restraint stress, CRS). Another group of mice were alternatively subjected to unpredictable mild stress in light or dark cycle listed in the following table for 21 days.

### Schedule for chronic unpredictable mild stress

**Table:** 

Day	Light cycle	Dark cycle
1	Swim in 24 °C cold water for 30 min	Keep the light on
2	Deprive of water and food	Put cage on a 45° slope
3	Deprive of water	Deprive of water
4	Restrain in a tube for 3 h	Keep the light on
5	Deprive of water and food	Deprive of water
6	Wet the bedding with 200 ml water	Put cage on a 45° slope
7	Swim in 24 °C cold water for 30 min	Keep the light on
8	Put cage in the dark	Deprive of water and food
9	Swim in 24 °C cold water for 30 min	Deprive of water and food
10	Deprive of water and food	Swim in 24 °C cold water for 30 min
11	Restrain in a tube for 3 h	Wet the bedding with 200 ml water
12	Isolate from companions for 3 h	Wet the bedding with 200 ml water
13	Deprive of water and food	Put cage on a 45° slope
14	Deprive of water	Keep the light on
15	Swim in 24 °C cold water for 30 min	Deprive of food
16	Restrain in a tube for 3 h	
17	Wet the bedding with 200 ml water	Wet the bedding with 200 ml water
18	Deprive of food	Wet the bedding with 200 ml water
19	Deprive of water and food	Deprive of water
20	Put cage on a 45° slope	Wet the bedding with 200 ml water
21	Restrain in a tube for 3 h	

### Pain threshold tests

The von Frey filaments (Anesthesio, San Jose, CA, USA) and a plantar anesthesia tester (Boerni, Tianjin, China) were used to measure the mechanical and thermal thresholds, respectively, of both hind paws of acclimatized mice in a test compartment on a wide gauge wire mesh supported by an elevated platform as described previously^7,33,71^.

The *von Frey* filaments ranged in force from 0.008 g to 4 g were used to measure mechanical threshold with an up-down method^7,65,72^. In brief, the first fiber (0.4 g) was applied to the hind paw until the fiber bent, and then held in place for 2 s. If this did not elicit a withdrawal response, the next higher force fiber was applied. This process continued until a withdrawal response was observed. If the mice responded, the fiber one fiber-force lower was applied on the subsequent trial. A cut-off value of 3.0 g in fiber force was assigned because this filament lifts the hind paw before it bends. The 50% paw withdrawal threshold (PWT) was calculated using Dixon’s up-down method^72^.

Thermal paw withdrawal latencies (PWL) in both hind paws were recorded with a plantar anesthesia tester (Boerni, Tianjin, China) in acclimatized mice in a test compartment on a glass surface^7,33,71^. A heat light source was positioned over the plantar surface of the hind paw and the withdrawal latency was measured. A 20 s cut-off time was used to prevent potential tissue damage to the surface of the paw.

### Conditioned place preference

In this test, mice with transfection of either eYFP or NpHR in VP ChAT neurons and with implantation of optical fibers in the VP were subjected to sham or SNI surgery. Four weeks after surgery, the conditioned place preference (CPP) was performed on these mice according to a previous study^65^ with some modifications (Supplementary Fig. S6). We used a two-chamber box (length × width × height: 40 × 20 × 30 cm^3^): the right chamber had black-and-white vertical stripes on the walls and a smooth white floor; the left chamber had black-and-white horizontal stripes on the walls and a mesh floor with gray background.

On Day 1 (pre-test session), mice were allowed to freely roam in the two chambers and the time that the mice explored in each chamber was recorded. From Day 2 to Day 4 (conditioning session), the mice were restricted in one chamber and received constant yellow light illumination of the VP for 20 min (2 min 3 mW constant yellow light with 2 min intervals) in the morning and were confined in the other chamber without illumination of the VP for 20 min in the afternoon. On Day 5 (test session), the mice were allowed to freely explore in the box for 20 min and the time spent in each chamber was recorded. On the pre-test and test days, the animal’s movement was video-tracked and analyzed offline with EthoVision XT 14.0 video tracking software (Noldus Information Technology, Wageningen, Netherlands). We calculated the time spent in the light-paired side on the pre-test and test days. Mice were not used if they spent more than 80% of the total time in either chamber in the pre-test session.

### Anxiety- and depression-like behavioural tests in mice

#### Open field test

Each mouse was placed in the center of a round open field arena (a cylinder, 30 cm in diameter, 40 cm in height) and allowed to explore freely for 20 min. Locomotor activity of the mice was recorded with a video camera controlled by Ethovision XT 14.0 software (Noldus Information Technology, Wageningen, Netherlands)^68^. The locomotor activity in the arena and the 15 cm-diameter central area was analyzed.

#### Tail suspension test

Tail suspension test (TST) is a behavioural task used for assessment of depression-like state^73^. Each mouse was hung from a bar about 50 cm above the ground by its tail using an adhesive tape that was placed about 1 cm away from the tip of the tail. Each mouse was tested only once for 6 min. The mice’s behaviour was recorded with a video camera from the side, and the duration of immobility was measured in the last 5 min.

#### Forced swim test

Forced swim test (FST) is a method to assess depression-like state^74^. Each mouse was placed in a glass cylinder (30 cm height and 20 cm diameter) that filled with water (25 ± 1 °C). The water level was 15 cm from the bottom. Each mouse was tested only once for 6 min. The animal’s behaviour was recorded with a video camera from the side, and the duration of immobility of each mouse during the last 5 min of the test was determined.

#### Elevated plus maze

Elevated plus maze (EPM) is a method to test anxiety levels^75,76^.

A Plexiglas maze in form of a plus was elevated 50 cm above the ground. The maze consists of a central platform (5 × 5 cm) and four arms: two open arms (30 × 5 cm) and two closed arms (30 × 5 cm, 20 cm tall walls) with open roofs. Same as the two closed arms, the two open arms are opposite to each other and converge into the central platform. A video camera was mounted above the maze to record mouse behaviour for 5 min. The video data were analyzed with EthoVision XT 14.0 software online or offline.

### Brain-slice patch-clamp recordings

Parasagittal slices (300 µm) were prepared with a vibratome (VT-1200S, Leica Microsystems, Nussloch, Germany) in ice-cold modified sucrose-based artificial cerebral spinal fluid (sACSF)^7,33,77,78^, saturated with 95% O_2_/5% CO_2_ (carbogen) containing (in mM) 85 NaCl, 75 sucrose, 2.5 KCl, 1.25 NaH_2_PO_4_, 4.0 MgCl_2_, 0.5 CaCl_2_, 24 NaHCO_3_, and 25 glucose. Slices were allowed to recover in sACSF at 32 °C for 75 min, and then held at room temperature in carbogenated normal ACSF containing (mM) 125 NaCl, 2.5 KCl, 1.25 NaH_2_PO_4_, 1.2 MgCl_2_, 2.4 CaCl_2_, 26 NaHCO_3_, and 11 glucose for at least 30 min before use.

Neurons in brain slices were visualized under an upright microscope (FN-1, Nikon) equipped with a CCD-camera (Flash 4.0 LTE, Hamamatsu). Whole-cell patch-clamp signals were recorded from neurons with a MultiClamp 700B amplifier, a Digidata 1550B analog-to-digital converter, and a pClamp 10.7 software (Molecular Devices, San Jose, CA). The patch electrodes had a resistance of 4–6 MΩ when filled with a low-chloride intrapipette solution containing (in mM) 135 K gluconate, 5 KCl, 0.2 EGTA, 0.5 CaCl_2_, 10 HEPES, 2 Mg-ATP, and 0.1 GTP^33,77^. The pH was adjusted to 7.2 with Tris-base and the osmolarity was adjusted to 300 mOsm with sucrose. To obtain light-evoked responses, blue light (460 nm, 5 or 300 ms, 2.5 mW, to activate ChR2) or lime light (550 nm, 1 s, 2 mW, to activate NpHR) was delivered through an optical fiber (200 µm, NA 0.37) connected to a PlexBright LED light source (Plexon Inc, Hong Kong, China).

### In vivo electrophysiological recordings

ChAT-Cre mice with viral vector (AAV-EF1α-DIO-ChR2-eYFP) injected into the VP were subjected to sham or SNI surgery. These mice were prepared for in vivo single-unit recordings 4–5 weeks after SNI surgery (6–7 weeks after virus injection). Before recordings, the mice were deeply anesthetized with sodium pentobarbital and stabilized on a stereotaxic apparatus (RWD Inc., Shenzhen, China). A 2 mm-diameter hole was made on the skull with the center at the coordinates above the BLA (AP: −1.4 mm; ML: 3.1 mm). An 8-channel optrode (ME-8-circle-L7-Diameter600μM-G1-R2-D50-OM16/1-2) (Plexon Inc, Hong Kong, China) was inserted into the hole and reached 4.5 mm deep from the Bregma above the BLA, then the optrode was lowered to probe neuronal spikes at the rate of 2–20 μm per step with a *z*-axis micromanipulator (Scientifica, East Sussex, United Kingdom) attached on the manipulator of the stereotaxic apparatus. Data were acquired with a Plexon neural data acquisition system composed of an Omniplex D system chassis, a 16-Channel DigiAmp subsystem, a PlexControl user interface software, and OmniPlex software (Plexon Inc, Hong Kong, China). One-minute traces were recorded only when visually identified spikes stably discharged for at least 2 min and the depth of the electrode was registered. A 5 s blue light stimulation (5 ms, 20 Hz, 4 mW) was delivered into the BLA through the optrode between 30 and 35 s to stimulate ChAT terminals from VP ChAT neurons. After experiments, the mice were sacrificed for histological confirmation of viral expression and location of the deepest electrodes (Supplementary Fig. S10b). The registered position of the electrodes were corrected according to the corresponding position of the deepest electrode in the mouse brain atlas.

The spikes were sorted according to their waveforms with an Offline Sorter V4 software (Plexon Inc., Hong Kong, China) and imported into NeuroExplore V5 (Plexon Inc., Hong Kong, China) from which spike rate and spike timestamps were exported in the format of Microsoft Excel file. SigmaPlot 14.0 (SPSS Inc.) was used to plot Figures and to analyze the data.

### Electrocardiograph recordings

Three groups of mice were respectively subjected to sham, SNI or SNI plus repetitive optogenetic inhibition of VP ChAT neurons and received two-lead electrocardiograph recordings (ECG). The mice were anesthetized with sodium pentobarbital and ECG recording was conducted with the Power Lab data acquisition system, controlled by the Lab Chart 8 software (ADInstrument, Sydney, Australia). The skin of the right fore limb, left and right hind limbs were cleaned, sterilized with 75% ethanol, and respectively connected to anode, cathode, and ground of the probe. Sampling rate and low-pass filter were set to 2 kHz and 500 Hz, respectively. ECG signals were recorded between 15–20 min after the mice were anesthetized. Heart rate, intervals between peaks of two consecutive QRS waves (RR interval), duration of QRS wave, and the variation of RR intervals were calculated with the Lab Chart 8 software (ADInstrument, Sydney, Australia).

### Measurement of cortisol and corticosterone in serum

Mice were euthanized with CO_2_. Blood was collected from ocular artery and centrifuged at 2500 *g* at 4 °C for 20 min. The enzyme-linked immunosorbent assay (ELISA) was performed to measure the concentration of cortisol and corticosterone with ELISA kits and a Thermo Scientific microplate reader (1510, Waltham, MA) according to manufacturer’s manual (JiangsuMeimian Industrial Co., Ltd, Yancheng, China).

### Histology

Mice were sacrificed in a CO_2_ chamber and then subjected to cardiac perfusion with phosphate-buffered saline (PBS), followed by 4% paraformaldehyde (PFA) in PBS. Mouse brains were removed and fixed in 4% PFA overnight at 4 °C. Brain samples were cut into 30 μm coronal sections with a CM1950 cryostat (Leica, Nussloch, Germany) and the sections were mounted onto glass slides. For immunostaining^33,40^, brain sections were incubated in a blocking buffer containing 5% donkey serum and 0.1% Triton X-100 for 90 min at room temperature. Then the sections were incubated with primary antibody diluted in blocking buffer for 48 h at 4 °C.

Primary antibodies include (1) rabbit anti-c-Fos IgG, 1:2000, c-Fos (9F6) Rabbit mAb, Cell Signaling Technology, Catalogue No. 2250 S; (2) Goat anti-ChAT IgG, 1:200, Millipore, Catalogue No. AB144P; (3) rat anti-SP IgG, 1:200, Sigma-Aldrich, Catalogue No. MAB356; (4) rabbit anti-Fluorogold, 1:1000, Millipore, Catalogue No. AB153; (5) Mouse anti-CaMKII, 1:300, Cell signaling Technology, Catalogue No. 50049; (6) Mouse anti-GAD67, 1:250, Millipore, Catalogue No. MAB5406.

After washing three times (10 min each) with PBS, the sections were incubated with secondary antibodies (Jackson ImmunoResearch) for 90 min at room temperature.

Secondary antibodies include (1) Donkey anti-rabbit Alexa 488, 1:500, Code: 711-545-152; RRID: AB_2313584; (2) Donkey anti-rabbit Cy3, 1:500, Code: 711-165-152; RRID: AB_2307443; (3) Donkey anti-rabbit Alexa 647, 1:500, Code: 711-605-152; RRID: AB_2492288; (4) Donkey anti-goat Alexa 488, 1:500, Code: 705-545-003; RRID: AB_2340428; (5) Donkey anti-goat Alexa 647, 1:500, Code: 705-605-003; RRID: AB_2340436; (6) Donkey anti-goat Cy3, 1:500, Code: 705-165-003; RRID: AB_2340411; (7) Donkey anti-rat Alexa 647, 1:500, Code: 712-605-150; RRID: AB_2340693; (8) Donkey anti-mouse Alexa 647, 1:500, Code: 715-605-150; RRID: AB_2340862.

The sections were washed three times (10 min each) with PBS, dried in the dark, and then cover-slipped in mounting medium.

The sections were imaged with a confocal microscope controlled by Zen2 software (LSM 880, Zeiss, Oberkochen, Germany) and the images were processed with ImageJ (NIH, Bethesda, MD, USA).

### Statistics and reproducibility

For each experiment, we did an initial test with a sample size of 5, calculated the variation (standard deviation) in the parameters, and used the power analysis function in SigmaPlot 14.0 software (*α* = 0.05, *β* = 0.85) to estimate the sample sizes needed to obtain reliable statistics. Sample sizes are all greater than these values. In experiments with positive results, SigmaPlot 14.0 gives P values and power values at alpha = 0.05. Power value > 0.80 means that it is likely to detect the actual difference. In experiments with negative results, we made sure that the sample sizes were similar to those in experiments with positive results.

For optogenetic and behavioural experiments, mice were randomly assigned to control/sham and experimental groups. All summarized data are expressed as the mean ± SEM. The error bars in histogram, time courses, and input-output curves are SEM (see Figs. 1b–d, 1h, 1j, k; 2c, 2f, g; 3b, c, 3e, f, 3h, 3j; 4b–k, 5f–h, 5j, k; 6d–g, 6i, j, 6l, m, 7b–d, 7f, g; 8f, 8k; 9d, e, 9h–l; 10c, 10e–i, 10l–p, and Figs. S1b, c, 1e, 1h; 2e, f, 2k, l; 3g, h; 4a–i, 4k–m; 5a–u; 6f, g, 6i, j; 7e–p; 8b, c, 8e, f; 9c, 9f, g, 9j; 10f, 10h; 12c–p; 13a–j; 14b–e). In box plots (Supplementary Fig. S10g, i), lower and higher bounds, central line, lower and higher whiskers respectively represent 25th, 75th, 50th, 5th, and 95th percentiles. Two-tailed paired or unpaired *t*-tests, two-way ANOVAs, one-way ANOVAs followed by post-hoc Bonferroni tests, and one-way repeated-measures ANOVAs with post-hoc Bonferroni tests and Geisser–Greenhouse correction were used to compare the behavioural, electrophysiological, and histological data, as indicated in the figure legends. If equal-variance and normal distribution assumptions were not valid, statistical significance was evaluated by the Mann-Whitney rank sum test or by two-way or one-way ANOVA rank test. Source data in all figures are provided in a Source Data file submitted with this paper. In all tests, a value of two-tailed *P* < 0.05 was considered statistically significant. GraphPad Prism (version 7.0) and SigmaPlot 14.0 were used for all statistical analyses.

When we finished each experiments, we sacrificed every mouse for histological evidence to confirm the expression of viral vectors and localization of cannula for drug delivery and optical fiber for optogenetic modulation and fiber photometry. The images in Figs. 1a, 1f, 1i, 2b, 5b, 6b, c, 6h, 6j, 7b, c and Supplementary Figs. S2b, 2h, 6c, 7c, d, 9a, b, and 10a, b, etc. are representative ones among all from mice we collected data in individual experiments.

### Reporting summary

Further information on research design is available in the Nature Portfolio Reporting Summary linked to this article.

## Supplementary information

Supplementary Information

Reporting Summary

## Data Availability

The data generated in this study are provided in the Source Data file submitted with this paper. Source data are provided with this paper.

## Source data

Source Data
