# Supplementary material for: Plasticity in ventral pallidal cholinergic neuron-derived circuits contributes to comorbid chronic pain-like and depression-like behaviour in male mice
Source: Nat Commun. 2023 Apr 17;14:2182. doi: 10.1038/s41467-023-37968-x (PMC10110548; doi:10.1038/s41467-023-37968-x)
Supplement: Supplementary file 1 — Supplementary Information [file 41467_2023_37968_MOESM1_ESM.pdf]

Supplementary figures

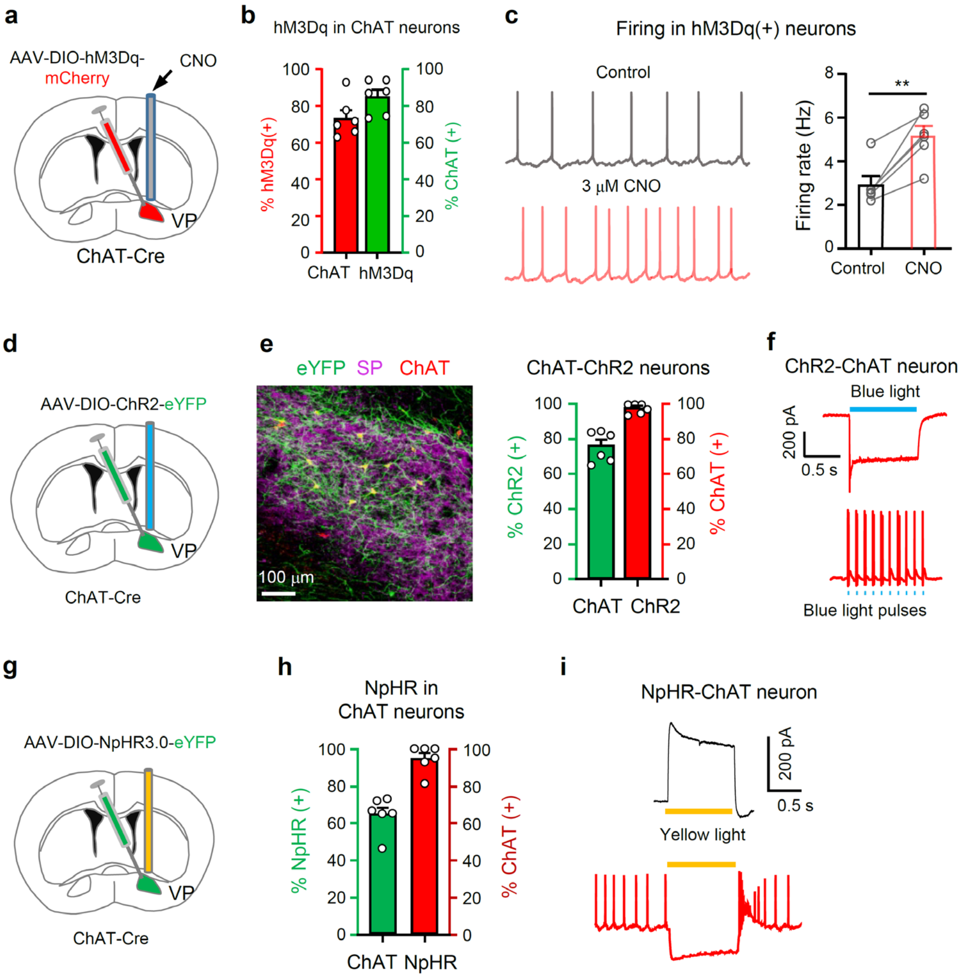

Figure S1 Efficiency and specificity of neuronal labeling with viral vectors. a-b The diagram and summary of efficiency (red bar) (72 ± 4.4%) and specificity (green bar) (84 ± 3.7%) of cholinergic (ChAT) neurons transfected with AAV-EF1α-DIO-hM3Dq-mCherry in the VP (n = 6 mice). **c** CNO increased firing rate in hM3Dq-labeled VP neurons (n = 6). t = 5.41, df = 5, P = 0.003, two-tailed paired *t*-test. **d** AAV-EF1α-DIO-ChR2-eYFP was injected into the VP of ChAT-Cre mice. **e** A representative image and summary showing the efficiency (green bar) (76 ± 3.5%) and specificity (red bar) (97 ± 1.1%) of ChR2-eYFP-labeling of VP ChAT neurons (n = 6). **f** In a ChR2-labeled VP ChAT neuron, 1 s blue light (2 mW) evoked an inward photocurrent in the voltage-clamp mode (upper panel) and 5 ms blue light (2 mW) at 10 Hz triggered firings in the current-clamped mode (lower panel). **g-h** The diagram and summary of efficiency

(green bar) (64 ± 3.9%) and specificity (red bar) (94 ± 3%) of NpHR-eYFP-labeling of VP ChAT neurons (n = 6). **i** In a NpHR-labeled VP cholinergic neuron, 1 s yellow light (2 mW) evoked an outward photocurrent in the voltage-clamp mode (upper panel) and a hyperpolarized membrane potential in the current-clamp mode (lower panel).

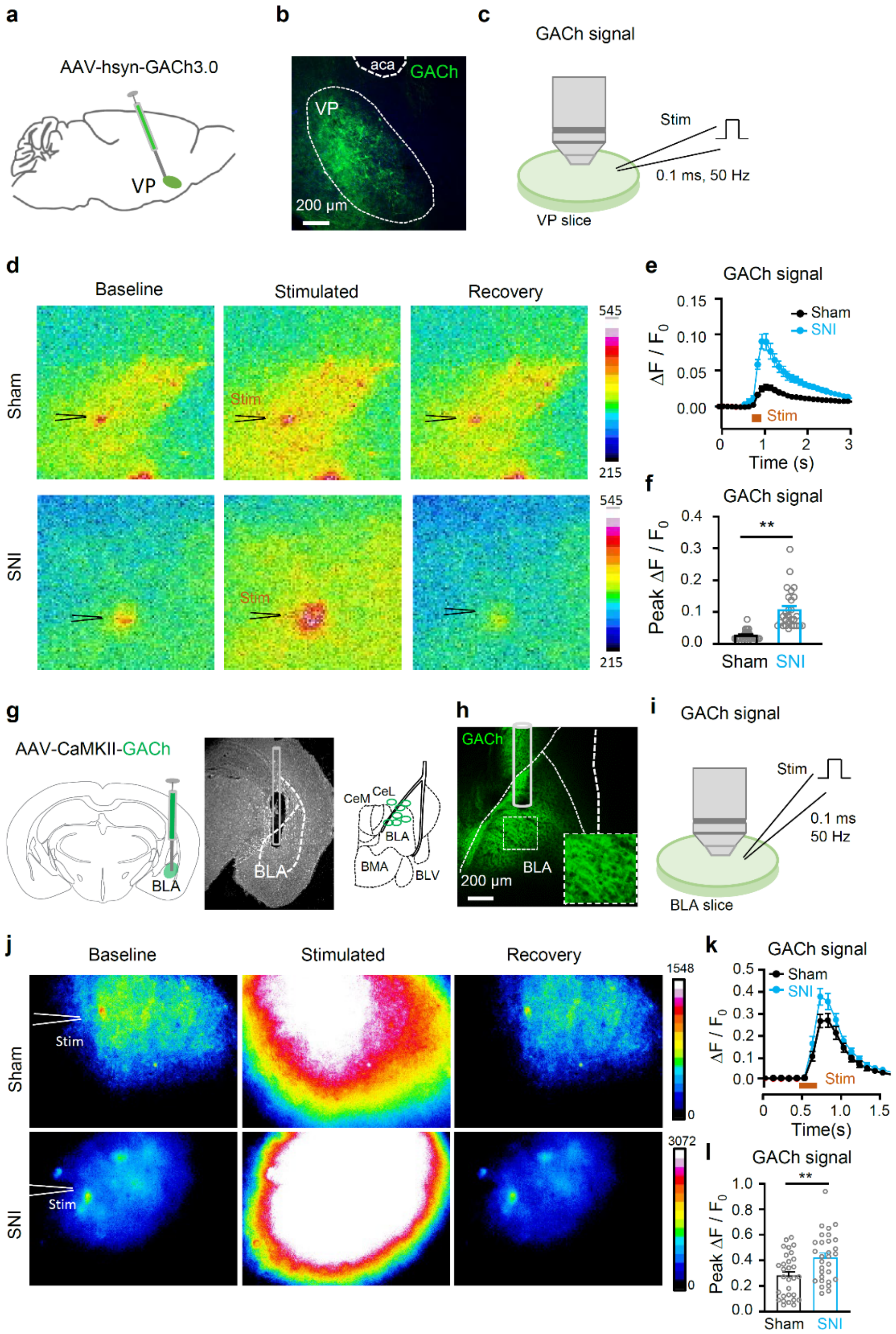

Figure S2 Acetylcholine release in the VP and BLA is enhanced in SNI mice.

a AAV-hSyn-GACh3.0, a viral vector, carrying acetylcholine (ACh) sensor (GACh3.0), was injected into the VP. **b** A representative image from 14 (one from each mouse) showing the expression of GACh3.0 in the VP. **c** Schematic diagram showing that

electrical stimulation-evoked ACh release in the VP of live brain slice preparations was monitored by measuring the green fluorescence intensity of GACh3.0 under an epi-fluorescent microscope equipped with a 40× water-immersion objective (WD: 3.2 mm; NA: 0.75). **d** Representative heatmaps showing that electrical stimulation (100 μs, 50 Hz 10 pulses) evoked ACh release in the VP of a sham mouse and an SNI mouse. **e** Summarized GACh responses (Mean ± SEM) to electrical stimulation (100 μs, 50 Hz 10 pulses) in the VP of 19 brain slices from sham (n = 7) and SNI (n = 7) mice (4 slices from each mouse). **f** Summary of electrical stimulation-evoked peak of GACh responses in **e**. t = 6.72, df = 36, P < 0.0001. Two-tailed t-test. **g** Schematic diagram showing that AAV-CaMKII-GACh3.0 was injected into the BLA. A representative bright field image and a summary showing the positions of optical fibers for Fig. 6d-f. **h** A representative image from 6 (one from each mouse) showing the expression of GACh in the BLA. **i** ACh release in the BLA was measured by illuminating GACh. **j-l** Representative heatmaps, summarized traces (n = 30 from 6 mice in each group), and peak amplitude of GACh responses to electrical stimulation (100 μs, 50 Hz 10 pulses). t = 3.10, df = 58, P = 0.003, sham vs SNI. Two-tailed t-test. ** P < 0.01

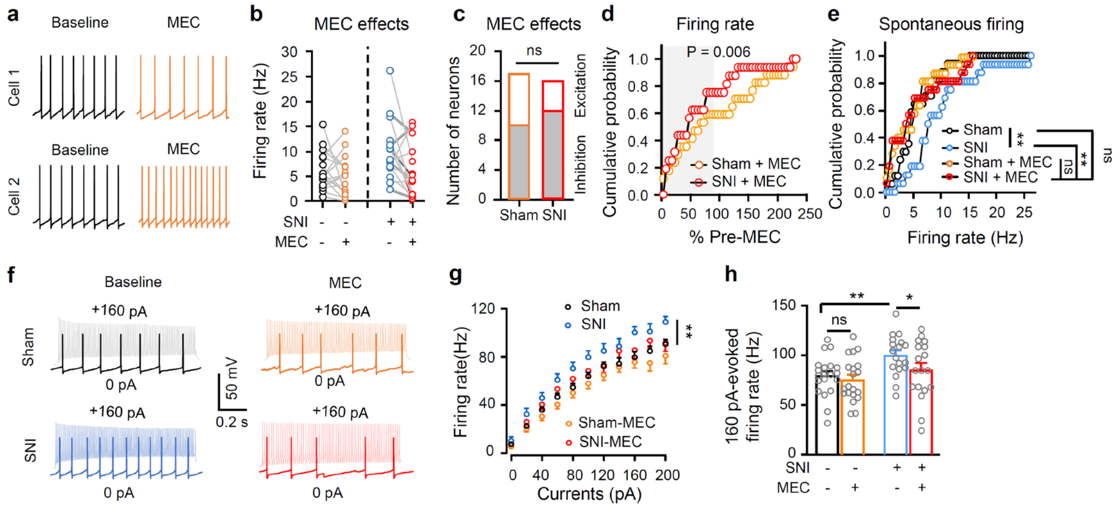

Fig. S3 Mecamylamine normalizes hyperactivity of VP non-ChAT neurons in SNI mice. ChAT-Cre mice were injected with AAV-EF1α-DIO-eYFP into the VP to label ChAT neurons with eYFP. Whole-cell patch-clamp recordings were performed on VP non-ChAT neurons from sham and SNI mice. **a-c** Representative traces, scatter plot, and summary showing that

mecamylamine (MEC, 10 μM) caused either inhibition or excitation of non-ChAT neurons in sham (n = 18 cells from 5 mice) and SNI (n = 15 cells from 5 mice) mice. **c** $x^2 = 0.97$, P = 0.32. Two-tailed Chi-square test. **d** Cumulative probability curves of firing rate during MEC incubation normalized to baseline firing rate in non-ChAT neurons of sham and SNI mice. KS = 0.45, P = 0.006, Kolmogorov-Smirnov (KS) test. **e** Cumulative probability curves of spontaneous firing rate of non-ChAT neurons from sham and SNI mice before and during MEC incubation. KS = 0.57, p < 0.0001, sham vs SNI; KS = 0.09, P = 0.97, sham vs sham + MEC; KS = 0.40, P = 0.0004, SNI vs SNI + MEC; KS = 0.19, P = 0.30, SNI + MEC vs sham + MEC. KS tests. **f-h** Representative traces, input-output curves, and summary of depolarizing current-evoked firing in non-ChAT neurons from sham and SNI mice. **g** The frequency of depolarizing current-evoked firing in VP non-ChAT neurons in sham (n = 18 cells from 5 mice) and SNI mice (n = 12 cells from 5 mice) with or without incubation of MEC. In baseline condition: Group, $F_{(1)} = 6.85$, P = 0.01; Currents, $F_{(12)} = 175.54$, P < 0.001; Interaction, $F_{(12)} = 1.12$, P = 0.34. In the presence of MEC: Group, $F_{(1)} = 1.85$, P = 0.18; Currents, $F_{(12)} = 241.72$, P < 0.001; Interaction, $F_{(12)} = 1.54$, P = 0.11. Two-way repeated measures ANOVA. **h** The frequency of 160 pA-evoked firing in non-ChAT neurons from sham mice (n = 18 cells from 5 mice) and SNI mice (n = 12 cells from 5 mice) with and without incubation of MEC. Group, $F_{(1)} = 6.55$, P = 0.015; MEC, $F_{(1)} = 4.37$, P = 0.04; Interaction, $F_{(1)} = 1.38$, P = 0.25. Two-way repeated measures ANOVA. * P < 0.05; ** P < 0.01; ns, not significant.

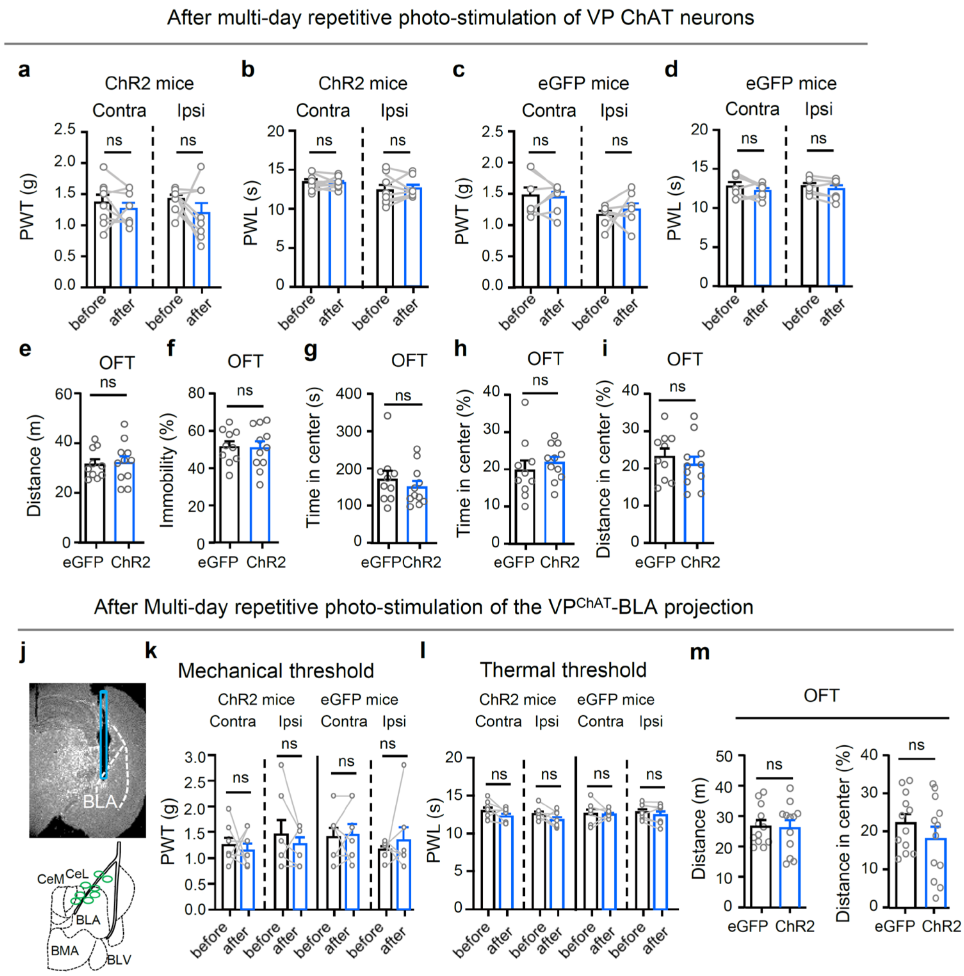

Figure S4 Repetitive optogenetic stimulation of VP cholinergic neurons and the VP-BLA cholinergic projection does not change pain thresholds and locomotion.

In mice subjected to multi-day repetitive optogenetic stimulation of VP cholinergic neurons or their terminals in the BLA (Fig. 5e, Fig. 9f), mechanical and thermal thresholds and locomotion were examined 2–3 days after discontinuation of blue light illumination in the VP.

a-b Mechanical and thermal thresholds on both sides in mice that were bilaterally injected with AAV-EF1α-DIO-ChR2-eYFP into the VP (ChR2 mice) before initiation and after discontinuation of repetitive light stimulation. $n = 8$. PWT: Contralateral, $t = 0.64$, $df = 7$, $P = 0.53$; Ipsilateral, $t = 1.31$, $df = 7$, $P = 0.21$. PWL: Contralateral, $t = 0.25$, $df = 7$, $P = 0.81$; Ipsilateral, $t = 0.31$, $df = 7$, $P = 0.76$. **c-d** Mechanical and thermal thresholds on both sides in mice that were bilaterally injected with AAV-EF1α-DIO-

eGFP into the VP (eGFP mice). n = 7. PWT: Contralateral, t = 0.26, df = 6, P = 0.81; Ipsilateral, t = 0.65, df = 6, P = 0.54. PWL: Contralateral, t = 1.32, df = 6, P = 0.24; Ipsilateral, t = 1.62, df = 6, P = 0.16. Parameters in locomotion test before initiation and 2–3 days after discontinuation of repetitive light stimulation were shown in **e-i**. **e** Distance traveled. t = 0.22, df = 19, P = 0.83. **f** Immobility ratio. t = 0.15, df = 19, P = 0.88. **g** Time in center. t = 0.44, df = 19, P = 0.79. **h** Time in center %. t = 0.73, df = 19, P = 0.48. **i** Distance in center. t = 0.76, df = 19, P = 0.46.

j Before the initiation of or two to three days after discontinuation of repetitive blue light illumination of the VP^{ChAT}-BLA projection, the mechanical **(k)** and thermal **(l)** thresholds measurement were performed in ChR2 (n = 7) and eGFP (n = 7) mice. **k** ChR2 mice: Contralateral, t = 0.54, df = 6, P = 0.60; Ipsilateral, t = 0.61, df = 6, P = 0.56. eGFP mice: Contralateral, t = 0.16, df = 6, P = 0.88; Ipsilateral, t = 0.68, df = 6, P = 0.51. **l** ChR2 mice: Contralateral, t = 1.21, df = 6, P = 0.25; Ipsilateral, t = 1.57, df = 6, P = 0.14. eGFP mice: Contralateral, t = 0.13, df = 6, P = 0.90; Ipsilateral, t = 0.59, df = 6, P = 0.57. **m** Locomotion between eGFP (n = 12) and ChR2 (n = 12) mice after discontinuation of repetitive blue light illumination of the VP^{ChAT}-BLA projection. Distance: t = 0.18, df = 22, P = 0.87; Distance in center: t = 1.12, df = 22, P = 0.28.

Two-tailed paired *t*-tests were used for **(a-d)** and **(k-l)**. Two-tailed *t*-test was used in **(e-i)**. Contra: contralateral; Ipsi: ipsilateral. ns, not significant.

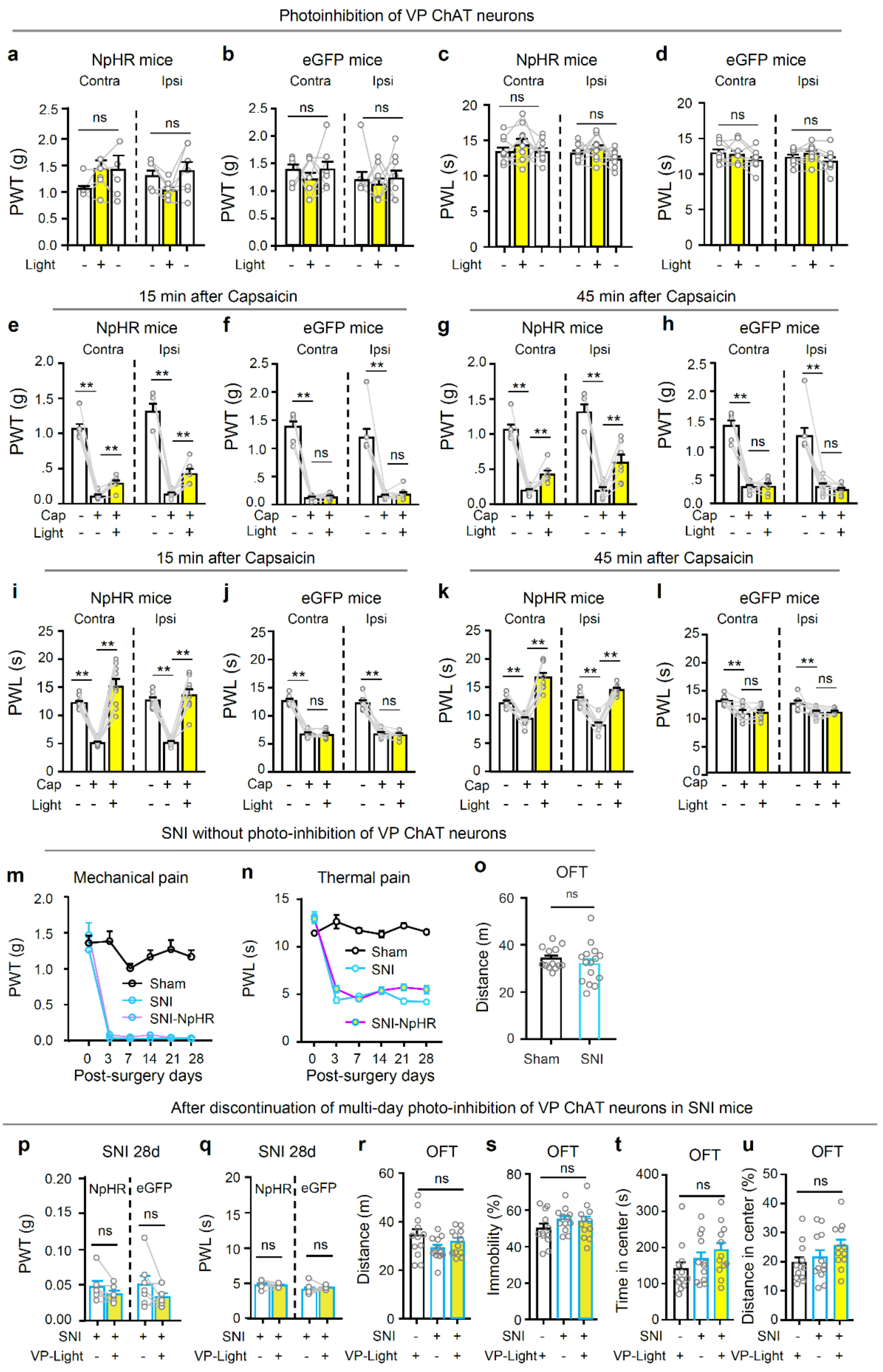

Figure S5 Effects of optogenetic inhibition of VP cholinergic neurons on mechanical and thermal thresholds. ChAT-Cre mice received injection of AAV-

EF1α-DIO-NpHR3.0-eYFP (NpHR mice) and AAV-EF1α-DIO-eGFP (eGFP mice) into the VP. **a-b** Yellow light illumination in the right VP did not change mechanical threshold on both sides in NpHR mice (n = 7) and eGFP mice (n = 7). NpHR mice: Contralateral, $F_{(1.757, 14.06)} = 2.45$, $P = 0.12$; Ipsiateral, $F_{(1.527, 12.22)} = 0.96$, $P = 0.40$. eGFP mice: Contralateral, $F_{(1.816, 12.71)} = 0.58$ = 0.58, $P = 0.58$; Ipsilateral, $F_{(1.983, 13.88)} = 0.13$, $P = 0.88$. **c-d** Yellow light illumination in the VP did not change thermal threshold on both sides in NpHR mice (n = 7) and eGFP mice (n = 7). NpHR mice: Contralateral, $F_{(1.552, 12.42)} = 0.78$, $P = 0.48$; Ipsilateral, $F_{(1.961, 15.69)} = 1.77$, $P = 0.20$. eGFP mice: Contralateral, $F_{(1.881, 13.17)} = 2.57$, $P = 0.11$; Ipsilateral, $F_{(1.129, 7.902)} = 2.71$, $P = 0.10$. **(e-f)** 15 min after capsaicin (Cap) injection in hind paws, yellow light illumination in the right VP increased mechanical threshold on both sides in NpHR mice (n = 7), but neither side in eGFP mice (n = 8). NpHR mice: Contralateral, $F_{(1.473, 8.837)} = 155.21$, $P < 0.001$; $t = 16.59$, $df = 6$, $P < 0.001$, baseline vs Cap; $t = 3.16$, $df = 6$, $P = 0.02$, Cap vs Cap-VP-light. Ipsilateral, $F_{(1.283, 7.698)} = 76.95$, $P < 0.001$; $t = 11.91$, $df = 6$, $P < 0.001$, baseline vs Cap; $t = 2.94$, $df = 6$, $P = 0.037$, Cap vs Cap-VP-light. eGFP mice: Contralateral, $F_{(1.063, 7.443)} = 169.74$, $P < 0.001$; $t = 12.92$, $df = 7$, $P < 0.001$, baseline vs CFA; $t = 0.59$, $df = 7$, $P = 0.57$; Ipsilateral, $F_{(1.081, 7.565)} = 51.95$, $P < 0.001$; $t = 7.90$, $df = 7$, $P < 0.001$, baseline vs Cap; $t = 0.86$, $df = 7$, $P = 0.42$, Cap vs Cap-light. **(g-h)** 45 min after Cap injection in lower hind legs, yellow light illumination in the right VP increased mechanical threshold on both sides in NpHR mice (n = 7), but on neither side in eGFP mice (n = 8). NpHR mice: Contralateral, $F_{(1.599, 9.597)} = 156.81$, $P < 0.001$; $t = 15.21$, $df = 6$, $P < 0.001$, baseline vs Cap; $t = 4.21$, $df = 6$, $P = 0.006$, Cap vs Cap-light; Ipsilateral, $F_{(1.283, 7.698)} = 53.64$, $P < 0.001$; $t = 17.59$, $df = 6$, $P < 0.001$, baseline vs Cap; $t = 3.60$, $df = 6$, $P = 0.01$, Cap vs Cap-light. eGFP mice: Contralateral, $F_{(1.410, 9.868)} = 83.36$, $P < 0.001$; $t = 10.06$, $df = 7$, $P < 0.001$, baseline vs Cap; $t = 0.34$, $df = 7$, $P = 0.74$, Cap vs Cap-light; Ipsilateral, $F_{(1.180, 8.260)} = 48.09$, $P < 0.001$; $t = 7.72$, $df = 7$, $P < 0.001$, baseline vs Cap; $t = 1.07$, $df = 7$, $P = 0.32$, Cap vs Cap-light. **(i-j)** 15 min after injection of Cap in hind paws, yellow light illumination in the right VP increased thermal

threshold on both sides in NpHR mice (n = 9), but on neither side in eGFP mice (n = 8). NpHR mice: Contralateral, $F_{(1.188, 9.508)}$ = 47.56, P < 0.001; t = 15.68, df = 8, P < 0.001, baseline vs Cap; t = 8.27, df = 8, P < 0.001, Cap vs Cap-light; Ipsilateral, $F_{(1.368, 10.94)}$ = 51.81, P < 0.001; t = 14.45, df = 8, P < 0.001, baseline vs Cap; t = 8.31, df = 8, P < 0.001, Cap vs Cap-light. eGFP mice: Contralateral, $F_{(1.770, 12.39)}$ = 75.71, P < 0.001; t = 17.22, df = 7, P < 0.001, baseline vs Cap; t = 0.26, df = 7, P = 0.8, Cap vs Cap-light; Ipsilateral, $F_{(1.574, 11.02)}$ = 78.00, P < 0.001; t = 11.25, df = 7, P < 0.001, baseline vs Cap; t = 0.35, P = 0.74, Cap vs Cap-light. **(K–L)** 45 min after Cap injection in lower hind legs, yellow light illumination in the right VP increased thermal threshold on both sides in NpHR mice (n = 9), but on neither side in eGFP mice (n = 8). NpHR mice: Contralateral, $F_{(1.489, 11.91)}$ = 50.24, P < 0.001; t = 5.13, P < 0.001, baseline vs Cap; t = 10.74, df = 8, P < 0.001, Cap vs Cap-light; Ipsilateral, $F_{(1.991, 15.93)}$ = 78.98, P < 0.001; t = 8.76, df = 8, P < 0.001, baseline vs Cap; t = 11.88, df = 8, P < 0.001, Cap vs Cap-light. eGFP: Contralateral, $F_{(1.927, 13.49)}$ = 8.76, P = 0.003; t = 4.14, df = 7, P = 0.008, baseline vs Cap; t = 0.27, df = 7, P = 0.80, Cap vs Cap-light; Ipsilateral, $F_{(1.084, 7.590)}$ = 9.07, P = 0.003; t = 2.87, df = 7, P = 0.04, baseline vs Cap; t = 0.66, df = 7, P = 0.52, Cap vs Cap-light. **m-n** Mechanical and thermal thresholds in Sham eGFP mice (n = 7), SNI eGFP mice (n = 7), and SNI NpHR mice (n = 8) after surgery on the left side. PWT: Time, $F_{(2.469, 46.91)}$ = 74.79, P < 0.0001; Group, $F_{(2, 19)}$ = 268, P < 0.0001. PWL: Time, $F_{(3.668, 69.69)}$ = 119.1, P < 0.0001; Group, $F_{(2, 19)}$ = 422.3, P < 0.0001. **o** Summary of distance traveled in sham mice (n = 12) and SNI mice (n = 14) in an open field arena. Distance: t = 0.84, df = 26, P = 0.41; Immobility: t = 0.73, P = 0.47. **p-q** Mechanical and thermal thresholds in SNI eGFP mice (n = 7) and SNI NpHR mice (n = 6) before and after repetitive yellow light illumination of the right VP. PWT: NpHR mice, t = 1.98, df = 5, P = 0.11; eGFP mice: t = 1.76, df = 6, P = 0.13. PWL: NpHR mice, t = 0.34, df = 5, P = 0.75; eGFP mice: t = 0.86, df = 6, P = 0.42. **r-u** Parameters in locomotion test in Sham-eGFP (n = 12), SNI-eGFP (n = 12), SNI-NpHR (n = 12) mice after repetitive yellow light illumination of the VP. Distance: $F_{(2, 33)}$ = 1.90, P = 0.17;

Immobility: $F_{(2, 33)}$ = 1.05, P = 0.36; Time in center: H = 4.07, P = 0.13; Distance in center: H = 4.00, P = 0.14.

One-way repeated measures ANOVAs with Bonferroni test were used in **(a-l)**. Two-way repeated measures ANOVAs were used in **(m-n)**. Two-tailed *t*-tests were used for **(o)**. Two-tailed paired *t*-tests were used for **(p-q)**. One-way ANOVA with Bonferroni test was used in (**r-s)**. Kruskal-Wallis one-way ANOVA on ranks test was used in **(t-u)**. ** P < 0.01; ns, not significant. Contra: contralateral; Ipsi: ipsilateral.

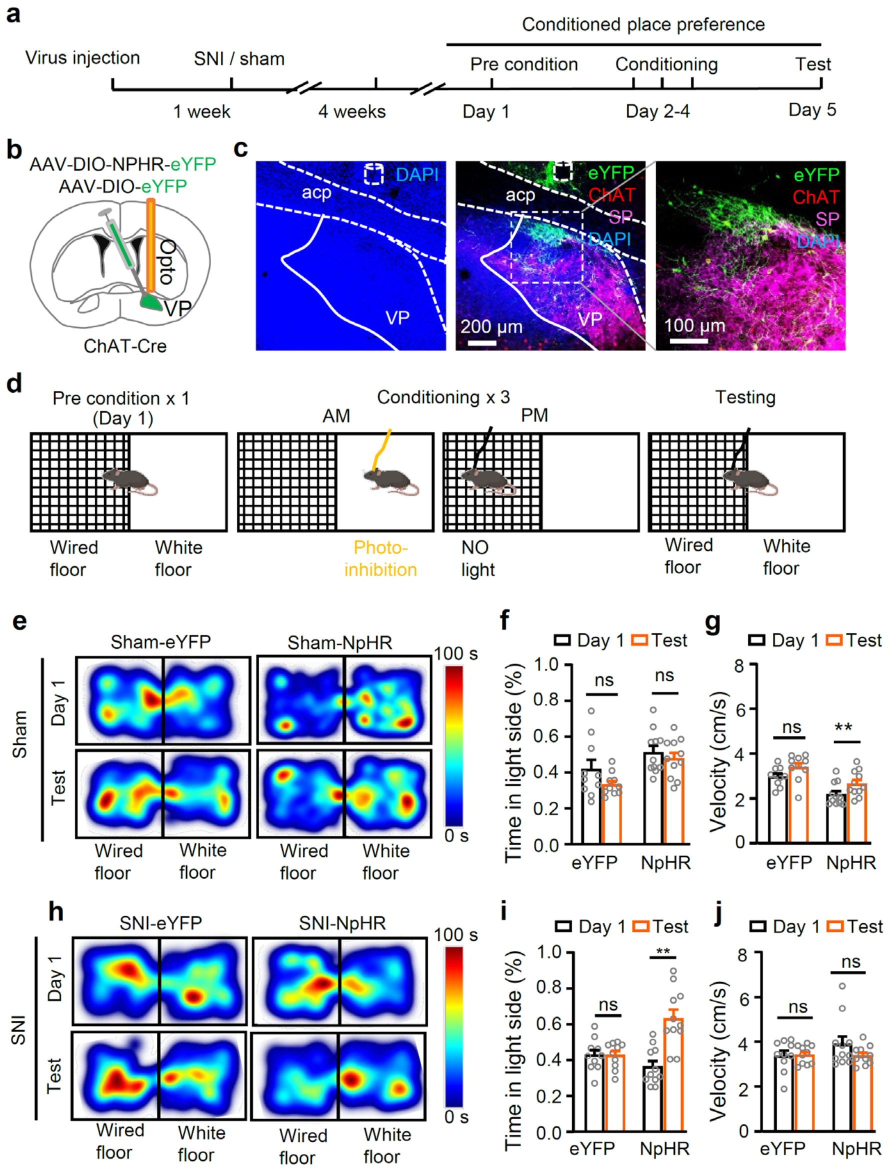

Fig. S6 Photoinhibition of VP ChAT neurons induces conditioned place preference. **a** Time line for photoinhibition of VP ChAT neurons-conditioned place preference. **b-**

c Schematic diagram and representative images from 10 (one from each mouse) showing the labeling of VP ChAT neurons with NpHR3.0-eYFP for photoinhibition. **d** Protocol for photo-inhibition-conditioned place preference. On Day 1, baseline preference to the two compartments with different walls and bottom in a chamber was tested. In Day 2-4, the mice were placed in the compartment with white floor and were paired with yellow light illumination (2 min constant yellow light with 2 min interval for 30 min) of the VP in the morning, and were placed in another compartment with meshed floor without light illumination in the afternoon. On Day 5, preference to the two compartments was tested. **e-g** Heatmaps and summary of time and movement velocity in the white compartment in sham mice. **f** Time in the white compartment before and after conditioning in sham eYFP mice (n = 10) and sham NpHR mice (n = 11). Group: $F_{(1, 19)}$ = 8.81, P = 0.008; Time: $F_{(1, 19)}$ = 3.36, P = 0.08; Interaction: $F_{(1, 19)}$ = 0.59, P = 0.45; sham-eYFP, n = 10, sham-NPHR, n = 11. **g** Movement velocity before and after conditioning in eYFP (n = 10) and NpHR (n = 11) sham mice. Group: $F_{(1, 19)}$ = 14.37, P <0.0001; Time: $F_{(1, 19)}$ = 17.47, P = 0.0005; Interaction: $F_{(1, 19)}$ = 0.06, P = 0.80. h-j Heatmaps and summary of time and movement velocity in the white compartment in SNI-eYFP mice (n = 10) and SNI-NpHR mice (n = 11). **i** Time in the white compartment. Group: $F_{(1, 19)}$ = 3.21, P = 0.09; Time: $F_{(1, 19)}$ = 19.55, P = 0.0003; Interaction: $F_{(1, 19)}$ = 19.61, P = 0.003. **j** Movement velocity. Group: $F_{(1, 19)}$ = 1.18, P = 0.29; Time: $F_{(1, 19)}$ = 1.33, P = 0.26; Interaction: $F_{(1, 19)}$ = 1.65, P = 0.22.

Two-way repeated measures ANOVAs were used for **(f-g)** and **(i-j)**. ** P < 0.01; ns, not significant.

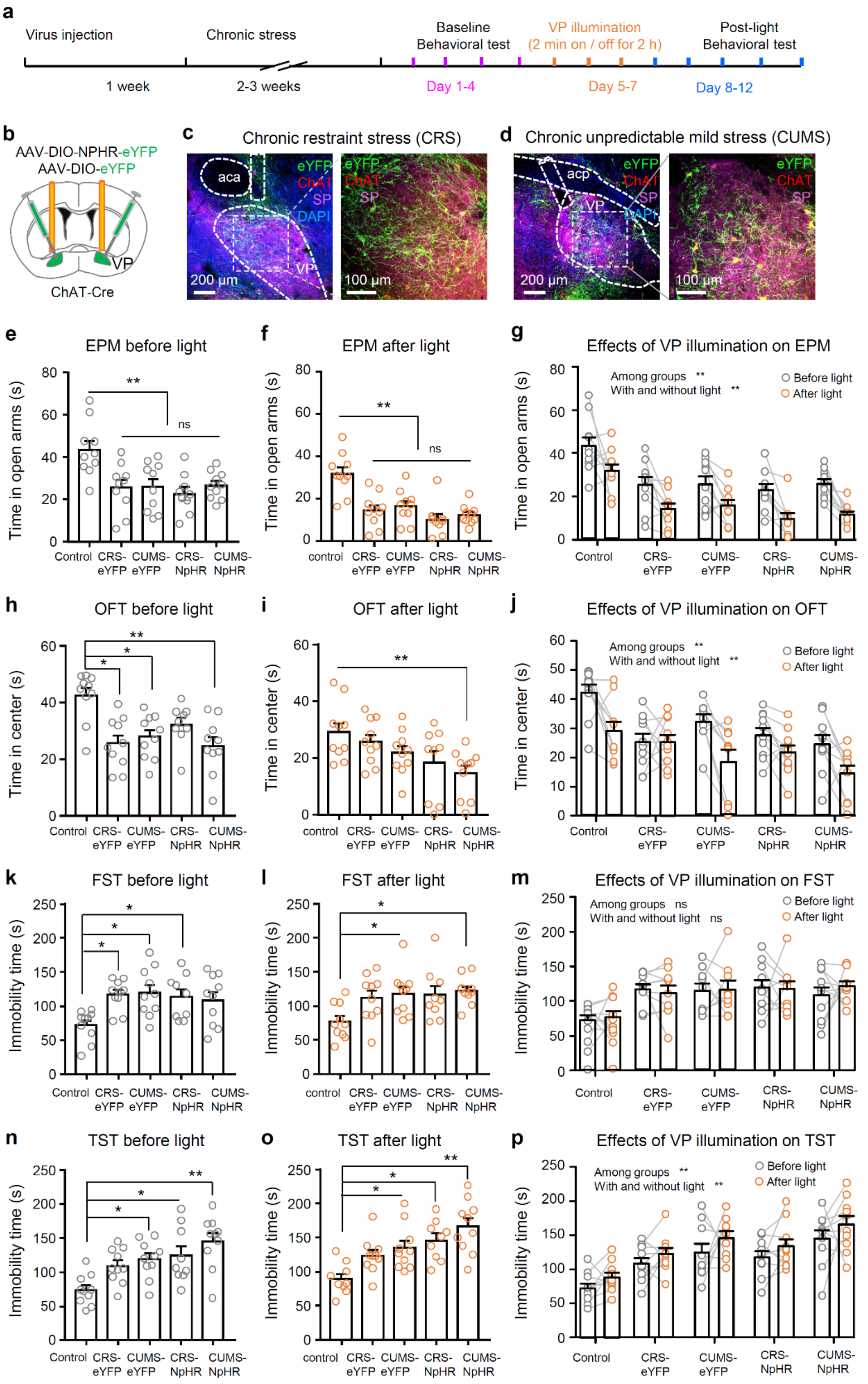

Fig. S7 Photoinhibition of VP ChAT neurons does not affect anxiety- and depression-like behavior in mice subjected to chronic restraint stress and chronic unexpected mild stress. **a** Time line of experiments, including viral vector transfection into VP ChAT neurons, chronic

stress treatement (2 weeks chronic restraint stress (CRS) or 4 weeks chronic unexpected mild stress (CUMS)), repetitive photoinhibition of VP ChAT neurons, and behavioral tests. **b-d** AAV-EF1a-DIO-NpHR-eYFP or AAV-EF1a-DIO-eYFP were injected into the VP of ChAT-Cre mice to label ChAT neurons. **c-d** Representative images from CRS mice (n = 10) and CUMS mice (n = 10). **e-g** Summary of parameters in the elevated plus maze (EPM) in control (n = 10), CRS-eYFP (n = 10), CUMS-eYFP (n = 9), CRS-NpHR (n = 10), and CUMS-NpHR (n = 10) mice before and after VP light illumination. **e** Baseline time in open arms. $F_{(4, 44)}$ = 6.04, P = 0.0006, one-way ANOVA. **f** Time in open arms after light in the VP. $F_{(4, 44)}$ = 12.18, P < 0.0001, one-way ANOVA. **g** Time in open arms before and after light. Group: $F_{(4, 44)}$ = 14.65, P < 0.001. Light: $F_{(1, 44)}$ = 44.81, P < 0.001. Interaction: $F_{(4, 44)}$ = 0.21, P = 0.93. Two-way repeated measures ANOVA. **h-j** Summary of time in the center in the open field test (OFT) in control (n = 10), CRS-eYFP (n = 10), CUMS-eYFP (n = 9), CRS-NpHR (n = 10), and CUMS-NpHR (n = 10) mice before and after VP light illumination. **h** Baseline time in center. $F_{(4, 44)}$ = 7.24, P = 0.0001; t = 4.40, df = 44, P = 0.0007, Control vs CRS-eYFP; t = 3.83, df = 44, P = 0.004, Control vs CUMS eYFP; t = 4.68, df = 44, P = 0.0003, Control vs CUMS-NpHR; t = 2.61, df = 44, P = 0.12, Control vs CRS-NpHR. **i** Time in center in the OFT after light. $F_{(4, 44)}$ = 3.69, P = 0.013. t = 3.45, df = 44, P = 0.01, Control vs CUMS-NpHR. **j** Time in center in the OFT before and after light. Group: $F_{(4, 44)}$ = 7.23, P < 0.001; light: $F_{(1, 44)}$ = 27.55, P < 0.001; Interaction: $F_{(4, 44)}$ = 2.44, P = 0.06. **k-m** Immobility time in the forced swim test (FST) in control (n = 10), CRS-eYFP (n = 10), CUMS-eYFP (n = 9), CRS-NpHR (n = 10), and CUMS-NpHR (n = 10) mice before and after VP light illumination. **k** Immobility time in FST before light. $F_{(4, 44)}$ = 4.08, P = 0.007. t = 3.27, df = 44, P = 0.02, Control vs CRS-eYFP; t = 3.50, df = 44, P = 0.01, Control vs CUMS-eYFP; t = 2.96, df = 44, P = 0.049, Control vs CRS-NpHR; t = 2.69, df = 44, P = 0.10, Control vs CUMS-NpHR. **l** Immobility time in FST after light. $F_{(4, 44)}$ = 3.56, P = 0.013; t = 3.01, df = 44, P = 0.04, Control vs CUMS-eYFP; t = 3.30, df = 44, P = 0.02, Control vs CUMS-NpHR. **m** Immobility time in FST before and after light. Group: $F_{(4, 44)}$ = 6.77, P < 0.001; Light: $F_{(1, 44)}$ = 0.21, P = 0.65; Interaction: $F_{(4, 44)}$ = 0.30, P = 0.88. **n-p** Immobility time in the tail suspension test (TST) in control (n = 10), CRS-eYFP (n = 10), CUMS-eYFP (n = 9), CRS-NpHR (n = 10), and CUMS-NpHR (n = 10) mice before and after

VP light illumination. **n** Immobility time in TST before light. $F_{(4, 44)} = 6.88$, $P = 0.0002$. $t = 3.19$, $df = 44$, $P = 0.03$, Control vs. CUMS-eYFP; $t = 3.49$, $df = 44$, $P = 0.01$, Control vs CRS-NpHR; $t = 5.07$, $df = 44$, $P < 0.0001$, Control vs CUMS-NpHR. **o** Immobility time in TST after light. $F_{(4, 44)} = 8.80$, $P < 0.001$. $t = 3.35$, $df = 44$, $P = 0.02$, Control vs CUMS-eYFP; $t = 4.06$, $df = 44$, $P = 0.002$, Control vs CRS-NpHR; $t = 5.65$, $df = 44$, $P < 0.0001$, Control vs CUMS-NpHR. **p** Immobility time in TST before and after light. Group: $F_{(4, 44)} = 15.82$, $P < 0.001$; Light: $F_{(1, 44)} = 7.80$, $P = 0.008$; Interaction: $F_{(4, 44)} = 0.05$, $P = 0.995$.

One-way ANOVAs with Bonferroni tests were used in **(e-f, h-i, k-l, n-o)**. Two-way repeated measures ANOVAs were used in **(g, j, m, p)**. * $P < 0.05$; ** $P < 0.01$; ns, not significant.

EPM: elevated plus maze; OFT: open field test; FST: forced swim test; TST: tail suspension test.

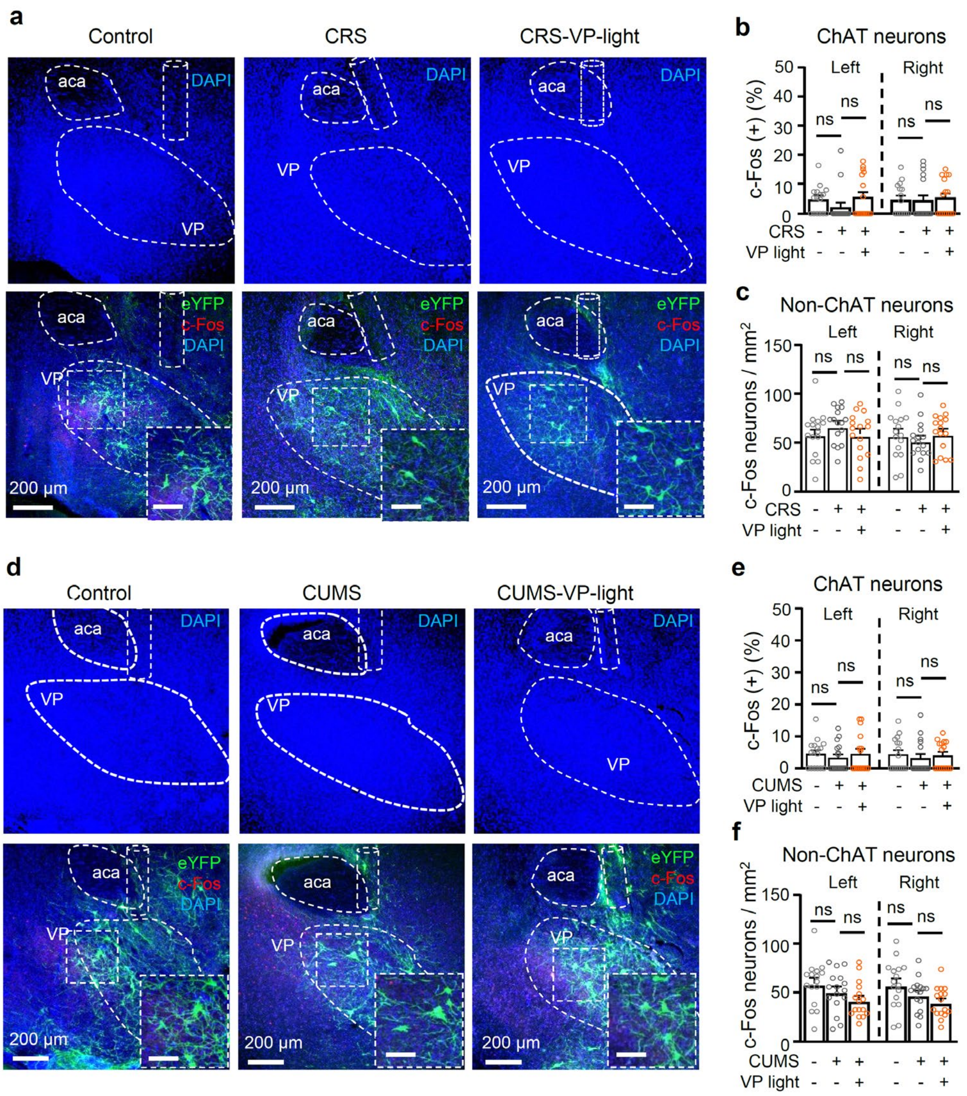

Fig. S8 VP neurons are not involved in chronic stress mice. The effects of repetitive inhibition of VP ChAT neurons on anxiety- and depression-like behaviours in eYFP and NpHR mice subjected to either chronic restraint stress (CRS) or chronic unpredictable mild stress (CUMS) were examined. **a-c** Representative images and summary showing c-Fos-positive ChAT and non-ChAT neurons in control, CRS-eYFP, CRS-NpHR mice. **b** c-Fos-positive ChAT neurons. Left VP: $F_{(2, 45)} = 1.52$, P = 0.23. Right VP: $F_{(2, 45)} = 0.11$, P = 0.90. **c** c-Fos-positive non-ChAT neurons. Left VP: $F_{(2, 45)} = 0.95$, P = 0.39. Right VP: $F_{(2, 45)} = 0.51$, P = 0.61. **d-f** Representative images and summary showing c-Fos-positive non-ChAT and ChAT neurons in control, CUMS-eYFP, CUMS-NpHR mice. **e** c-Fos-positive

ChAT neurons. Left VP: $F_{(2, 45)} = 0.33$, P = 0.72. Right VP: $F_{(2, 45)} = 0.24$, P = 0.79. **f** c-Fos-positive non-ChAT neurons. Left VP: $F_{(2, 45)} = 2.70$, P = 0.08. Right VP: $F_{(2, 45)} = 3.70$, P = 0.03. Sham vs CRS: t = 1.54, df = 45, P = 0.24; CRS vs CRS-Light: t = 1.17, df = 45, P = 0.25. One-way ANOVAs with Bonferroni tests were used for **(b-c, e-f)**. n = 16 sections from 4 mice in each group. ns, not significant. Scale bar in insets: 100 μm.

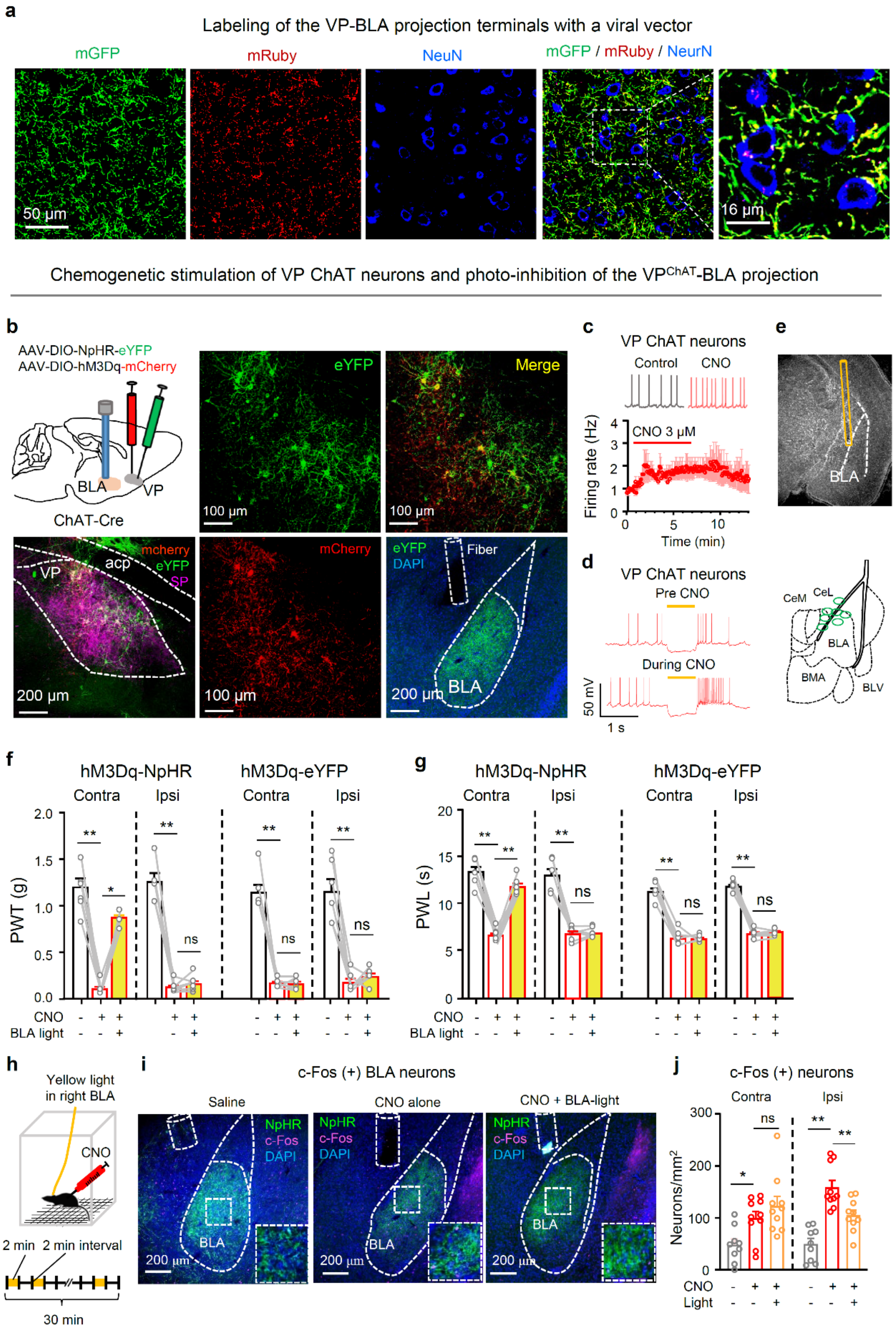

Figure S9 The VP^{ChAT}-BLA projection partially mediates analgesic effects of optogenetic inhibition of VP cholinergic neurons. a Representative images of 5 (one from each mice) taken from the BLA showing that injection of AAV-EF1α-DIO-

mGFP-Synaptophysin-mRuby into the VP of ChAT-Cre mice allows labeling of cholinergic terminals in the BLA. **b** The combination of AAV-EF1α-DIO-hM3Dq-mCherry with AAV-EF1α-DIO-NpHR3.0-eYFP or AAV-EF1α-DIO-hM3Dq-mCherry with AAV-EF1α-DIO-eYFP were simultaneously injected into the right VP of ChAT-Cre mice. Representative images of 5 sets (one set from each mice) showing the expression of two viral vectors in VP ChAT neurons and their fibers in the BLA, and an optical fiber implantation above the BLA. **c** The hM3Dq-mCherry-labeled VP cholinergic neurons were excited by bath perfusion of 3 μM CNO (n = 5). **d** On VP ChAT neurons expressing both hM3Dq-mCherry and NpHR-eYFP, CNO enhanced firing rate and yellow light hyperpolarized the membrane potential, resulting in silence of the neuron. **e** A representative image and summary showing the locations of optical fiber in the BLA for behavioral tests in **(f)** and histological assay in **(h-j)**. **f-g** Mechanical and thermal thresholds of hind paws of mice that received injection of viral vectors into the right VP before, 30 min after CNO i.p. injection, and during light illumination of the right BLA 45 min after CNO injection. **f** PWT. NpHR mice (n = 7): Contralateral (Contra), $F_{(1.221, 7.327)} = 93.98$, $P < 0.001$, $t = 20.76$, $df = 6$, $P < 0.001$, CNO vs CNO-BLA-light; Ipsilateral (Ipsi): $F_{(1.140, 6.837)} = 115.72$, $P < 0.001$, $t = 1.10$, $df = 6$, $P = 0.3$, CNO vs CNO-BLA-light. eYFP mice (n = 6): Contra, $F_{(1.091, 5.454)} = 121.02$, $P < 0.001$, $t = 0.36$, $df = 5$, $P = 0.74$, CNO vs CNO-BLA-light; Ipsi, $F_{(1.349, 6.744)} = 45.76$, $P < 0.001$, $t = 0.99$, $df = 5$, $P = 0.37$, CNO vs CNO-BLA-light. **g** PWL. NpHR mice (n = 7): Contra, $F_{(1.671, 10.03)} = 101.68$, $P < 0.001$, $t = 10.46$, $df = 6$, $P < 0.001$, CNO vs CNO-BLA-light; Ipsi: $F_{(1.118, 6.707)} = 59.45$, $P < 0.001$, $t = 0.18$, $df = 6$, $P = 0.86$, CNO vs CNO-BLA-light. eYFP mice (n = 6): Contra, $F_{(1.960, 9.798)} = 127.23$, $P < 0.001$, $t = 0.10$, $df = 5$, $P = 0.91$, CNO vs CNO-BLA-light; Ipsi, $F_{(1.282, 6.412)} = 311.95$, $P < 0.001$, $t = 0.58$, $df = 5$, $P = 0.59$, CNO vs CNO-BLA-light. **h** Three groups of mice received viral vectors to label VP ChAT neurons with hM3Dq + NpHR (n = 7). Yellow light (2 min episodes with 2 min intervals for 30 min) was delivered into the right BLA in these mice 45 min after saline or CNO i.p. injection. **i-j** Representative images and summary showing c-Fos-positive neurons in the BLA on both sides. Contra: $F_{(2, 26)} = 6.79$, P =

0.004, t = 1.19, df = 26, P = 0.24, CNO vs CNO-BLA-light. Ipsi: $F_{(2, 26)}$ = 22.42, P < 0.001; t = 3.40, df = 26, P = 0.007, CNO vs CNO-BLA-light. Saline, n = 9 sections; CNO, n = 10 sections; CNO-BLA-light, n = 10 sections. These sections in each group were from 5 mice.

One-way repeated measures ANOVA with Bonferroni test and Geisser-Greenhouse correction was used in **(f-g)**. One way ANOVA with Bonferroni test was used in **(j)**. Contra: contralateral; Ipsi: ipsilateral. * P < 0.05; ** P < 0.01; ns, not significant.

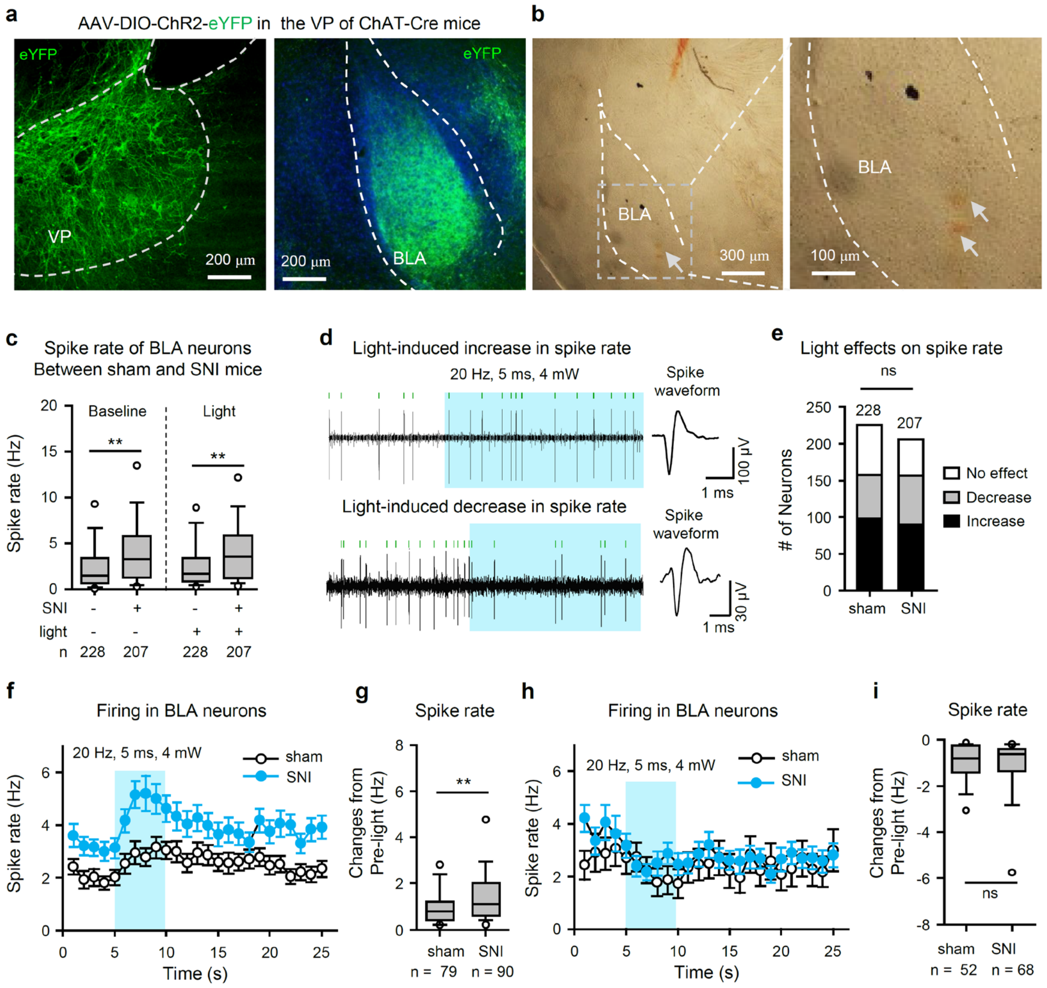

Fig. S10 Photostimulation of the VP-BLA cholinergic projection regulates BLA neurons in sham and SNI mice. a One set of representative images from 10 sets (one set from each mouse) showing that AAV-EF1α-DIO-ChR2-eYFP was injected into the VP of ChAT-Cre mice and these neurons projected to the BLA. **b** Representative image

from 10 (one from each mouse) showing two spots in the BLA as deepest locations of recording electrodes. **c** Comparison of spike rates of single-units in the BLA from sham and SNI mice before and during photostimulation of the VP-BLA cholinergic projection. Sham: n = 228 units from 5 mice; SNI: n = 208 units from 5 mice. H = 53.64. Baseline spike rate: Q = 5.72, P < 0.001, sham vs SNI. Spike rate during light stimulation (5 ms, 20 Hz, 4 mW): Q = 4.33, P < 0.001, sham vs SNI. Kruskal-Wallis one-way ANOVA on ranks with Dunn's method for pairwise comparison. **d-e** Representative traces (3 s), spike waveforms, and summary showing that photostimulation of the VP-BLA ChAT projection increased spike rates in some neurons, but decreased spike rates in some other neurons. x^2 = 3.05, P = 0.22, sham vs SNI, two-tailed Chi-square test. **f-g** Time courses and summary of increase in spike rates following photostimulation of the VP-BLA ChAT projection in some BLA neurons in sham and SNI mice. T = 5815, P = 0.004, sham: n = 99 units, SNI: n = 90 units. Mann-Whitney rank sum test (two-tailed) with Yates continuity correction. **h-i** Time courses and summary of decrease in spike rates following photo-stimulation of the VP-BLA ChAT projection in sham and SNI mice. T = 1749, P = 0.92, sham: n = 60 units, SNI: n = 68 units. Mann-Whitney rank sum test with Yates continuity correction. ** P < 0.01; ns, not significant.

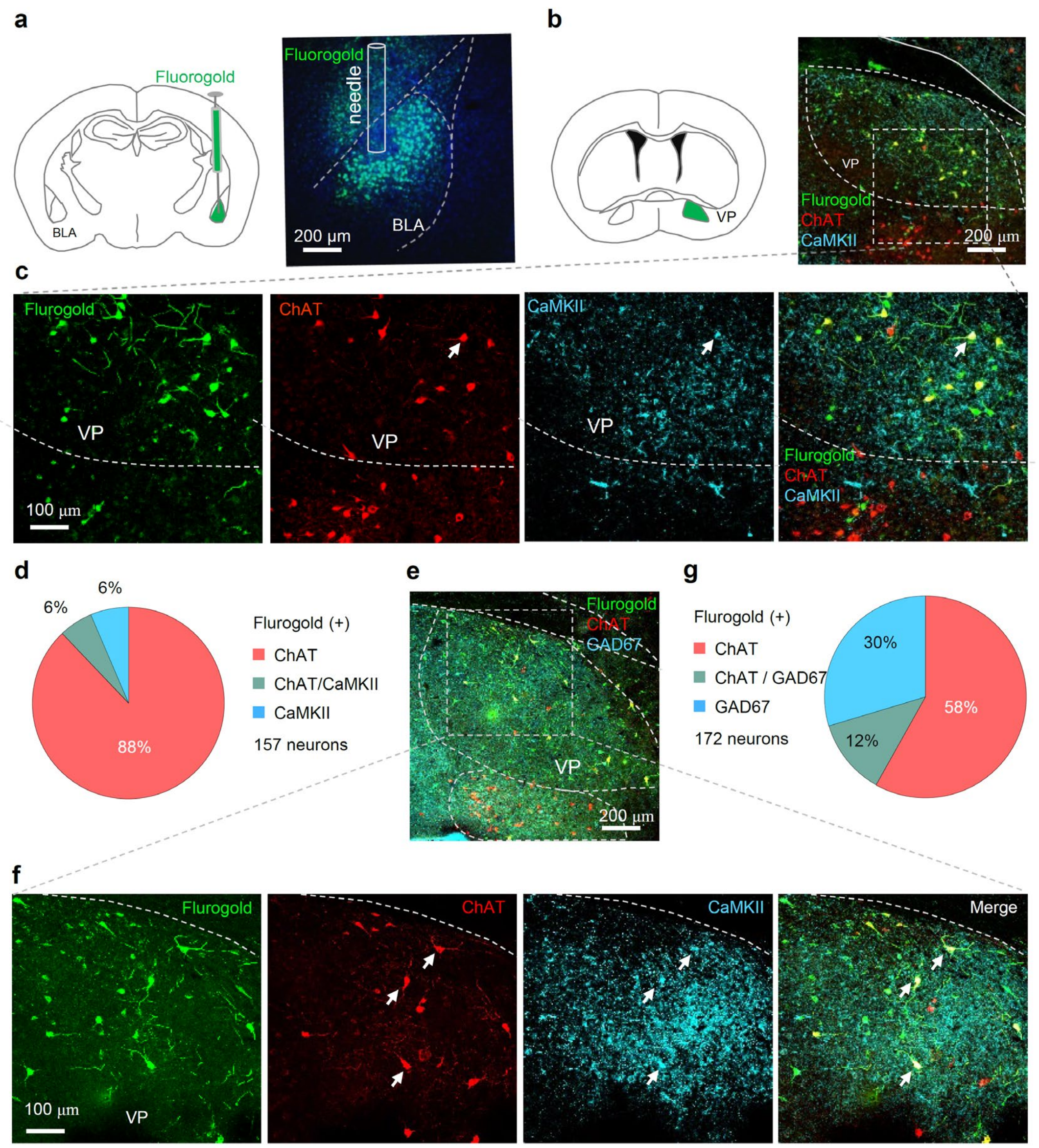

Fig. S11 Neurochemical properties of retrogradely labeled VP-BLA neurons. **a** Schematic diagram and a representative image showing microinjection of 2% Fluorogold into the basolateral amygdala (BLA). **b-c** Schematic diagram and representative images showing the labeling of Flurogold, ChAT, and CaMKII in the VP. **d** A pie chart showing rare overlapping of ChAT and CaMKII in Flurogold-labeled VP neurons. Data were from 15 sections from 3 mice. **e-f** Representative images showing the labeling of Flurogold, ChAT, and GAD67 in the VP. **g** A pie chart showing low incidence of overlapping of ChAT and GAD67 in Flurogold-labeled VP neurons. Data were from 15 sections from 3 mice.

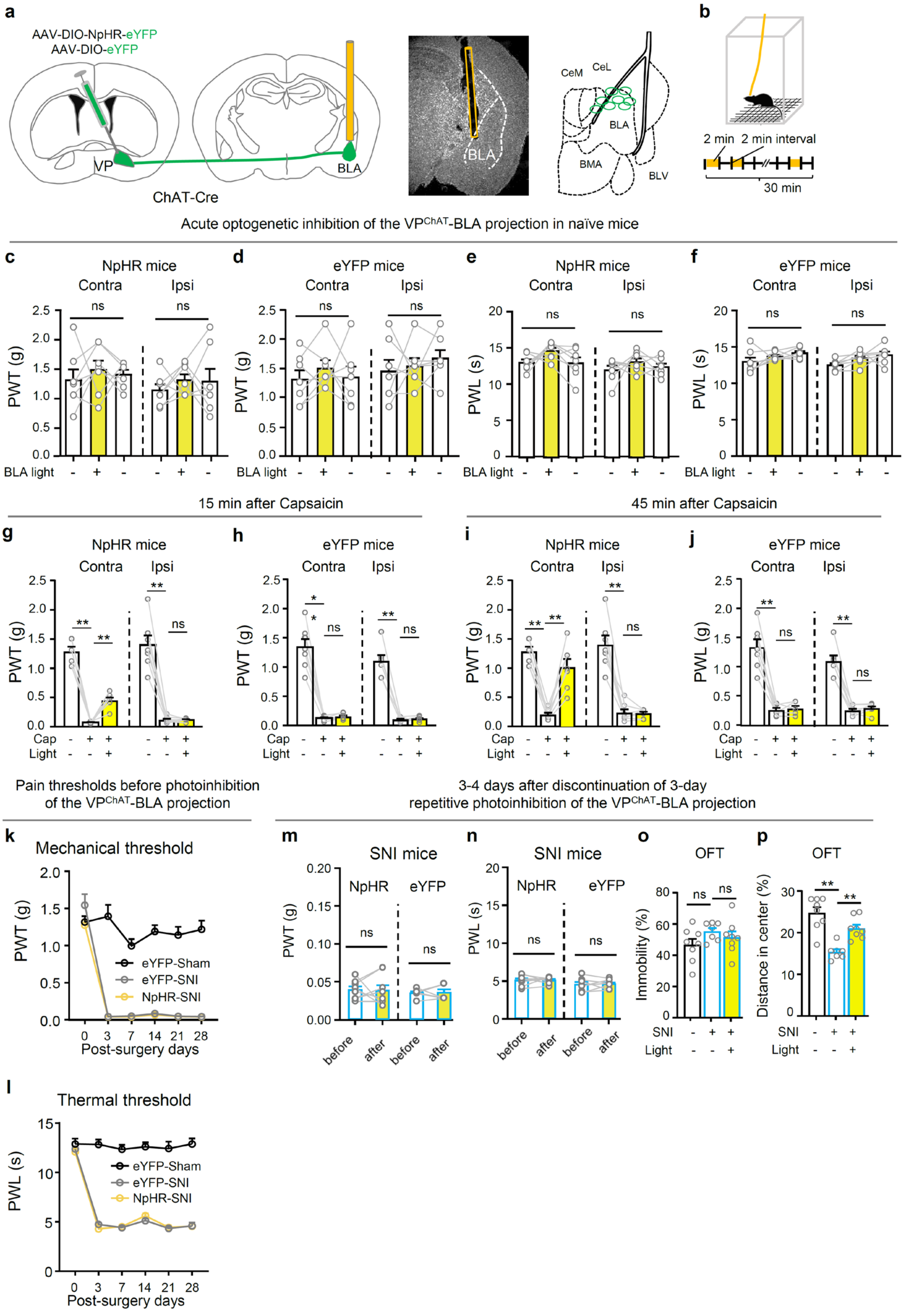

Figure S12 Optogenetic inhibition of the VP^{ChAT}-BLA projection regulates pain threshold. a-b AAV-EF1α-DIO-NpHR-eYFP or AAV-EF1α-DIO-eYFP was injected into the right VP of ChAT-Cre mice and an optical fiber was implanted above the right

BLA. These mice were respectively referred as VP ChAT-NpHR mice and VP ChAT-eYFP mice. Diagram showing 30 min optogenetic inhibition of the VP^{ChAT}-BLA projection. **c-d** Mechanical threshold on hind paws of VP ChAT-NpHR and VP ChAT-eYFP mice were measured before, during, and after yellow light illumination of the right BLA. NpHR mice (n = 7): Contralateral (Contra), $F_{(1.360, 8.163)} = 0.37$, P = 0.70; Ipsilateral (Ipsi), $F_{(1.582, 9.494)} = 0.35$, P = 0.71. eYFP (n = 7): Contra, $F_{(1.581, 9.483)} = 0.43$, P = 0.66; Ipsi, $F_{(1.494, 8.963)} = 0.41$, P = 0.67. **e-f** Thermal threshold on hind paws of VP ChAT-NpHR and VP ChAT-eYFP mice were measured before, during, and after yellow light illumination of the right BLA. NpHR mice (n = 7): Contra, $F_{(1.478, 8.866)} = 2.25$, P = 0.15; Ipsi, $F_{(1.538, 9.226)} = 0.85$, P = 0.45. eYFP mice (n = 7): Contra, $F_{(1.322, 7.935)} = 1.90$, P = 0.19; Ipsi, $F_{(1.202, 7.213)} = 3.65$, P = 0.06. **g-j** Mechanical threshold on hind paws were measured before and during yellow light illumination of the right BLA in VP ChAT-NpHR and VP ChAT-eYFP mice that received Capsaicin (Cap) injection in the lower hind legs. **g-h** 15 min after Cap injection. NpHR mice (n = 7): Contra, $F_{(1.506, 9.036)} = 147.25$, P < 0.001; t = 13.72, df = 6, P < 0.001, baseline vs Cap; t = 7.34, df = 6, P < 0.001, Cap vs Cap-light; Ipsi, $F_{(1.021, 6.125)} = 69.75$, P < 0.001; t = 8.65, df = 6, P < 0.001, baseline vs Cap; t = 0.057, df = 6, P = 0.96, Cap vs Cap-light. eYFP mice (n = 7): Contra, $F_{(1.044, 6.264)} =72.26$, P < 0.001; t = 8.82, df = 6, P < 0.001, baseline vs Cap; t = 1.07, df = 6, P = 0.32, Cap vs Cap-light; Ipsi, $F_{(1.071, 6.429)} = 108.69$, P < 0.001; t = 10.20, df = 6, P < 0.001, baseline vs Cap; t = 0.66, df = 6, P = 0.53, Cap vs Cap-light. **i-j** 45 min after Cap injection. NpHR mice (n = 7): Contra, $F_{(1.525, 9.151)} = 40.20$, P < 0.001; t = 12.49, df = 6, P < 0.001, baseline vs Cap; t = 6.18, df = 6, P < 0.001, Cap vs Cap-light; Ipsi, $F_{(1.129, 6.775)} = 48.96$, P < 0.001; t = 7.12, df = 6, P < 0.001, baseline vs Cap; t = 0.17, df = 6, P = 0.86, Cap vs Cap-light. eYFP mice (n = 7): Contra, $F_{(1.078, 6.468)} = 53.87$, P < 0.001; t = 7.80, df = 6, P < 0.001, baseline vs Cap; t = 0.74, df = 6, P = 0.49, Cap vs Cap-light; Ipsi, $F_{(1.171, 7.028)} = 76.32$, P < 0.001; t = 9.30, df = 6, P < 0.001, baseline vs Cap; t = 1.21, df = 6, P = 0.27, Cap vs Cap-light. **k-l** Mechanical and thermal thresholds of eYFP-Sham (n = 8), eYFP-SNI (n = 8), and NpHR-SNI (n = 8) mice with optical fiber in the BLA. PWT: Time, $F_{(3.271, 68.70)} = 75.81$, P < 0.0001; Group, $F_{(2, 21)} =$

500.7, $P < 0.0001$. PWL: Time, $F_{(4.048, 85.01)} = 113.3$, $P < 0.0001$; Group, $F_{(2, 21)} = 417.1$, $P < 0.0001$. **m-n** Repeated photoinhibition of the VP^{ChAT}-BLA projection did not change baseline mechanical and thermal thresholds in NpHR-SNI mice (n = 8) and eYFP-SNI mice (n = 7). PWT: NpHR, t = 0.89, df = 7, P = 0.44; eYFP, t = 0.78, df = 6, P = 0.39. PWL: NpHR, t = 0.96, df = 7, P = 0.48; eYFP, t = 0.86, df = 6, P = 0.42. **o-p** Locomotion in sham mice (n = 8), SNI mice (n = 7), and SNI mice after repetitive photoinhibition of the VP-BLA ChAT projection. Immobility: $F_{(2, 20)} = 1.51$, P = 0.24. Distance in center: $F_{(2, 20)} = 19.21$, $P < 0.001$; t = 6.18, df = 20, $P < 0.001$, sham vs SNI; t = 3.72, df = 20, P = 0.004, SNI vs SNI-BLA-light.

One-way repeated measures ANOVAs with Bonferroni test and Geisser-Greenhouse correction were used in **(a-l)**. Two-way repeated measures ANOVAs with Geisser-Greenhouse correction were used in **(k-l)**. Two-tailed paired *t*-test was used in **(m-n)**. One-way ANOVAs with Bonferroni tests were used in **(o-p)**. Contra: contralateral; Ipsi: ipsilateral. ** $P < 0.01$; ns, not significant.

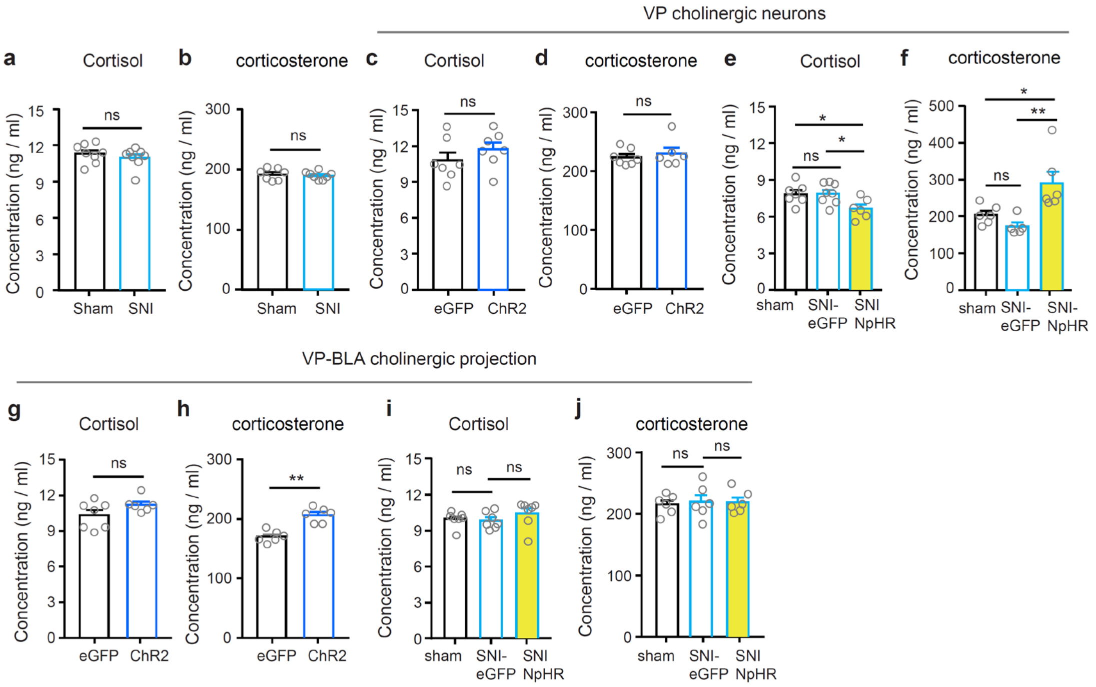

Fig. S13 Bidirectional repetitive modulation of VP ChAT neurons and the VP^{ChAT}-BLA projection exert complex effects on serum concentration of cortisol and

corticosterone. a Serum concentrations of cortisol in sham and SNI mice. t = 0.86, df = 14, P = 0.41, sham (n = 8) vs SNI (n = 8). **b** Serum concentrations of corticosterone in sham and SNI mice. t = 0.47, df = 13, P = 0.65, sham (n = 8) vs SNI (n = 8). **c** Serum concentrations of cortisol in eGFP and ChR2 mice subjected to repetitive blue light illumination in the VP. t = 1.03, df = 12, P = 0.32, sham (n = 7) vs SNI (n = 7). **d** Serum concentrations of corticosterone in eGFP and ChR2 mice subjected to repetitive blue light illumination in the VP. t = 0.56, df = 11, P = 0.57, eGFP (n = 7) vs ChR2 (n = 7). **e** Serum concentrations of cortisol in sham-eGFP (n = 7), SNI-eGFP (n = 8) and SNI-NpHR (n = 6) mice subjected to repetitive yellow light illumination in the VP. $F_{(2, 18)}$ = 4.56, P = 0.03; t = 2.57, df = 18, P = 0.05, sham-eGFP vs SNI-NpHR; t = 2.74, df = 18, P = 0.04, SNI-eGFP vs SNI-NpHR. **f** Serum concentrations of corticosterone in sham-eGFP (n = 7), SNI-eGFP (n = 8), and SNI-NpHR (n = 6) mice subjected to repetitive yellow light illumination in the VP. $F_{(2, 14)}$ = 8.19, P = 0.004; t = 2.94, df = 14, P = 0.02, sham-eGFP vs SNI-NpHR; t = 3.85, df = 14, P = 0.005, SNI-eGFP vs SNI-NpHR. **g** Serum concentrations of cortisol in sham-eGFP (n = 7) and ChR2 (n = 6) mice subjected to repetitive blue light illumination in the BLA. t = 1.69, df = 11, P = 0.12, eGFP vs ChR2. **h** Serum concentrations of corticosterone in eGFP (n = 7) and ChR2 (n = 6) mice subjected to repetitive blue light illumination in the VP. t = 5.62, df = 10, P = 0.002, eGFP vs ChR2. **i** Serum concentrations of cortisol in sham-eGFP (n = 7), SNI-eGFP (n = 7) and SNI-NpHR (n = 7) mice subjected to repetitive yellow light illumination in the BLA. $F_{(2, 18)}$ = 0.93, P = 0.41. **j** Serum concentrations of corticosterone in sham-eGFP (n = 6), SNI-eGFP (n = 6), and SNI-NpHR (n = 6) mice subjected to repetitive yellow light illumination in the BLA. $F_{(2, 15)}$ = 0.06, P = 0.94.

Two-tailed t-tests were used for a-d, and g-h. One-way ANOVAs with Bonferroni tests were used for e-f and i-j. * P < 0.05; ** P < 0.01; ns, not significant.

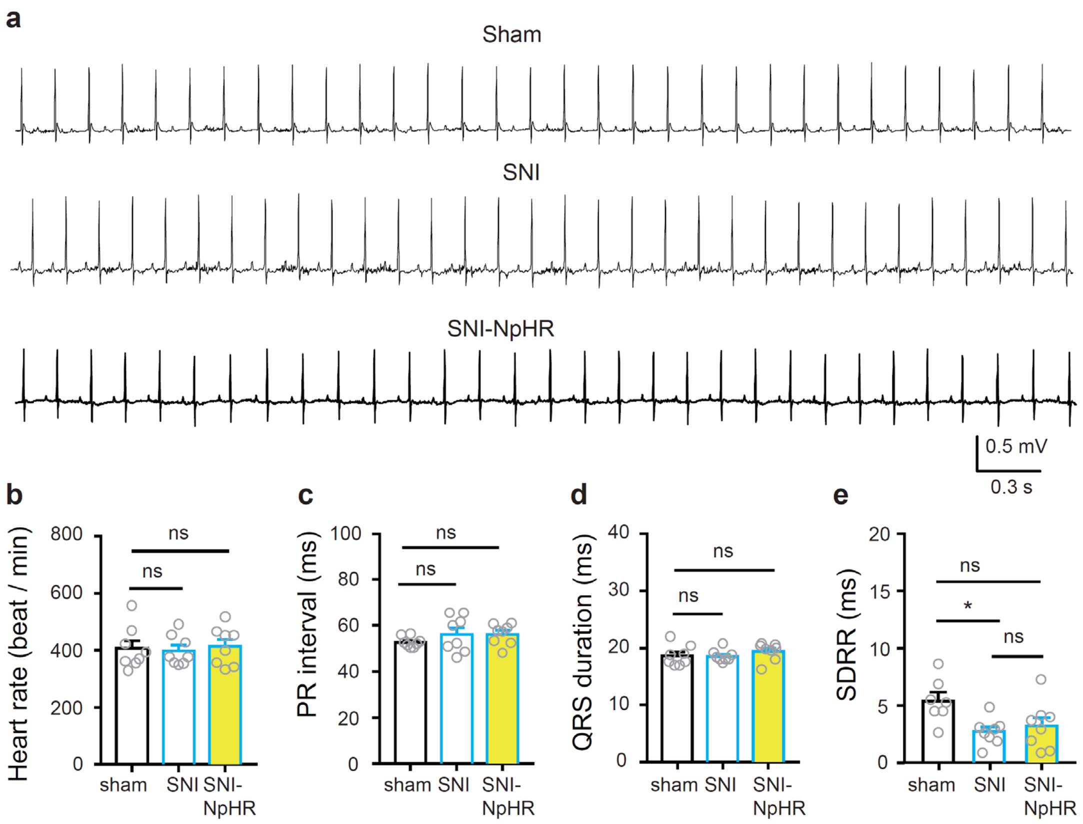

Fig. S14 Electrocardiograph recordings in SNI mice with anxiety- and depression-like behaviors. **a** Typical traces of electrocardiograph (ECG) recordings from sham, SNI, and SNI+repetitive photoinhibition of VP ChAT neurons. **b-e** Comparison of ECG parameters among sham, SNI, and SNI+repetitive optogenetic inhibition of VP ChAT neurons. **b** Heart rate. $F_{(2, 21)} = 0.12$, $P = 0.88$. **c** RR intervals. $F_{(2, 21)} = 1.11$, $P = 0.35$. **d** QRS duration. $_{(2, 21)}= 0.81$, $P = 0.46$. **e** Standard deviation of RR intervals. $F_{(2, 21)} = 4.93$, $P = 0.01$. $t = 2.99$, df = 14, $P = 0.02$, sham vs SNI. $t = 2.42$, df = 14, $P = 0.08$, sham vs SNI+NpHR. $t = 0.59$, df = 14, $P = 0.99$.

One-way ANOVA with Bonferroni tests were used for **(b-e)**. n = 8 mice in each group. * $P < 0.05$. ns, not significant.
