# Supplementary material for: Plasticity in ventral pallidal cholinergic neuron-derived circuits contributes to comorbid chronic pain-like and depression-like behaviour in male mice
Source: Nat Commun. 2023 Apr 17;14:2182. doi: 10.1038/s41467-023-37968-x (PMC10110548; doi:10.1038/s41467-023-37968-x)
Supplement: Supplementary file 2 — Reporting Summary [file 41467_2023_37968_MOESM2_ESM.pdf]

nature portfolio

Corresponding author(s): Jun-Li Cao, Chunyi Zhou, Cheng Xiao

Last updated by author(s): Mar 18, 2023

Reporting Summary

Nature Portfolio wishes to improve the reproducibility of the work that we publish. This form provides structure for consistency and transparency in reporting. For further information on Nature Portfolio policies, see our Editorial Policies and the Editorial Policy Checklist.

Statistics

For all statistical analyses, confirm that the following items are present in the figure legend, table legend, main text, or Methods section.

n/a	Confirmed	
☐	☒	The exact sample size (n) for each experimental group/condition, given as a discrete number and unit of measurement
☐	☒	A statement on whether measurements were taken from distinct samples or whether the same sample was measured repeatedly
☐	☒	The statistical test(s) used AND whether they are one- or two-sided<br>*Only common tests should be described solely by name; describe more complex techniques in the Methods section.*
☒	☐	A description of all covariates tested
☐	☒	A description of any assumptions or corrections, such as tests of normality and adjustment for multiple comparisons
☐	☒	A full description of the statistical parameters including central tendency (e.g. means) or other basic estimates (e.g. regression coefficient) AND variation (e.g. standard deviation) or associated estimates of uncertainty (e.g. confidence intervals)
☐	☒	For null hypothesis testing, the test statistic (e.g. F, t, r) with confidence intervals, effect sizes, degrees of freedom and P value noted<br>*Give P values as exact values whenever suitable.*
☒	☐	For Bayesian analysis, information on the choice of priors and Markov chain Monte Carlo settings
☒	☐	For hierarchical and complex designs, identification of the appropriate level for tests and full reporting of outcomes
☒	☐	Estimates of effect sizes (e.g. Cohen's d, Pearson's r), indicating how they were calculated

Our web collection on statistics for biologists contains articles on many of the points above.

Software and code

Policy information about availability of computer code

Data collection	We used commercially available softwares suitable to our hardwares to collect data. The softwares include PClamp 10.7, Ethovision XT 14.0, Zen 2, OmniPlex neural data acquisition system, and LabChart 8.0.
Data analysis	We used GraphPad Prism 7.0, SigmaPlot 14.0, Clampfit 10.7, Offline sorter V4, Neuroexplorer V5, and LabChart 8.0 for data analysis in this manuscript.

For manuscripts utilizing custom algorithms or software that are central to the research but not yet described in published literature, software must be made available to editors and reviewers. We strongly encourage code deposition in a community repository (e.g. GitHub). See the Nature Portfolio guidelines for submitting code & software for further information.

Data

Policy information about availability of data

All manuscripts must include a data availability statement. This statement should provide the following information, where applicable:

- Accession codes, unique identifiers, or web links for publicly available datasets
- A description of any restrictions on data availability
- For clinical datasets or third party data, please ensure that the statement adheres to our policy

The source data included in main figures and supplementary figures are provided as a supplementary file submitted with this paper. Raw data will be provided upon request.

Human research participants

Policy information about studies involving human research participants and Sex and Gender in Research.

Reporting on sex and gender	N/A
Population characteristics	N/A
Recruitment	N/A
Ethics oversight	N/A

Note that full information on the approval of the study protocol must also be provided in the manuscript.

Field-specific reporting

Please select the one below that is the best fit for your research. If you are not sure, read the appropriate sections before making your selection.

[x] Life sciences [ ] Behavioural & social sciences [ ] Ecological, evolutionary & environmental sciences

For a reference copy of the document with all sections, see nature.com/documents/nr-reporting-summary-flat.pdf

Life sciences study design

All studies must disclose on these points even when the disclosure is negative.

Sample size	For each experiment, we did an initial test with a sample size of 5, calculated the variation (standard deviation) in the parameters, and used the power analysis function in SigmaPlot 14.0 software (α = 0.05, β = 0.85) to estimate the sample sizes needed to obtain reliable statistics. Sample sizes are all greater than these values.<br>We confirmed the sufficiency of the sample size for each experiment according to power value. Traditionally, power value > 0.80 means that the experiment in this condition is likely to detect the real difference. In experiments with positive results, SigmaPlot 14.0 gives P values and power values at alpha = 0.05. In experiments with negative results, we made sure that the sample sizes were similar to those in experiments with positive results.
Data exclusions	In neuromodulation experiments, we excluded data from mice with misplacement of cannula or optical fibers or mice that viral vectors did not express well.
Replication	We confirm that all results are able to be successfully replicated. In establishment of acute and persistent pain models, we did more than 10 cohorts in this study and had a 100% success rate. For neuromodulation experiments, we did the same manipulation at least twice 3 or more days apart. For morphological experiments, we repeated the results that CFA and SNI mice showed hyperactivity in the VP-BLA pathway in Fig. 3d-f, 6h-m, 7e-g, 8j-k, 9g-h, 10b-e, 10k-l. Data in these figures came from different cohorts of mice. We performed electrophysiological recordings for two purposes. For qualitative experiments, we confirmed whether we recorded the reported phenomenon in >=5 neurons in our study. For instance, to confirm the function of transfected ChR2, NpHR, and hM3Dq in neurons. For quantitative assay, we recorded more than 3 neurons from each mice and repeated in more than 5 mice in brain slice patch-clamp recordings, and recorded as many neurons as we could from 5 mice subjected to each treatment for in vivo single-unit recordings.
Randomization	We allocated animals randomly into different groups.
Blinding	The experimenters performing behavioral and morphological assays did not know the drugs injected through cannula or the viral vectors transfected for optogenetic / chemogenetic modulation or with / without neuromodulation or pain states of the mice. The experimenters performing electrophysiological recordings did not know whether the mice were subjected to SNI, but those aiming to verify the function of actuators carried by viral vectors were not blind to types of viral vectors injected into the mouse brain.

Reporting for specific materials, systems and methods

We require information from authors about some types of materials, experimental systems and methods used in many studies. Here, indicate whether each material, system or method listed is relevant to your study. If you are not sure if a list item applies to your research, read the appropriate section before selecting a response.

Materials & experimental systems

n/a	Involved in the study	
	x	Antibodies
x		Eukaryotic cell lines
x		Palaeontology and archaeology
	x	Animals and other organisms
x		Clinical data
x		Dual use research of concern

Methods

n/a	Involved in the study	
x		ChIP-seq
x		Flow cytometry
x		MRI-based neuroimaging

Antibodies

Antibodies used	Antibodies used in this study were all purchased from Millipore, Cell Signaling Technology, Sigma-Aldrich, and Jackson ImmunoResearch. These antibodies have been widely used in the field.<br>Primary antibodies include 1) rabbit anti-c-Fos IgG, 1:2000, c-Fos (9F6) Rabbit mAb, Cell Signaling Technology, Catalogue No. 2250S; 2) Goat anti-ChAT IgG, 1:200, Millipore, Catalogue No. AB144P; 3) rat anti-SP IgG, 1:200, Sigma-Aldrich, Catalogue No. MAB356; 4) rabbit anti-Fluorogold, 1:1000, Millipore, Catalogue No. AB153; 5) Mouse anti-CaMKII, 1:300, Cell signaling Technology, Catalogue No. 50049; 6) Mouse anti-GAD67, 1:250, Millipore, Catalogue No. MAB5406.<br>Secondary antibodies from Jackson ImmunoResearch include 1) Donkey anti-rabbit Alexa 488, 1:500, Code: 711-545-152; RRID: AB_2313584; 2) Donkey anti-rabbit Cy3, 1:500, Code: 711-165-152; RRID: AB_2307443; 3) Donkey anti-rabbit Alexa 647, 1:500, Code: 711-605-152; RRID: AB_2492288; 4) Donkey anti-goat Alexa 488, 1:500, Code: 705-545-003; RRID: AB_2340428; 5) Donkey anti-goat Alexa 647, 1:500, Code: 705-605-003; RRID: AB_2340436; 6) Donkey anti-goat Cy3, 1: 500, Code: 705-165-003; RRID: AB_2340411; 7) Donkey anti-rat Alexa 647, 1:500, Code: 712-605-150; RRID: AB_2340693; 8) Donkey anti-mouse Alexa 647, 1:500, Code: 715-605-150; RRID: AB_2340862.
Validation	These antibodies were validated by the manufacturers and numerous scientists and the results are available in the websites of manufacturers. The websites and representative citations of primary antibodies are: 1) rabbit anti-c-Fos IgG, https://www.cellsignal.cn/products/primary-antibodies/c-fos-9f6-rabbit-mab/2250, PMID: 36198341; 2) Goat anti-ChAT IgG, https://www.sigmaaldrich.cn/CN/en/product/mm/ab144p, PMID: 27100197; 3) rat anti-SP IgG, https://www.sigmaaldrich.cn/CN/en/product/mm/mab356, PMID: 29403029; 4) rabbit anti-Fluorogold, https://www.sigmaaldrich.cn/CN/en/product/mm/ab153i, PMID: 35149515; 5) Mouse anti-CaMKII, https://www.cellsignal.cn/products/primary-antibodies/camkii-a-6g9-mouse-mab/50049?site-search-type=Products&N=4294956287&Ntt=50049&fromPage=plp&_requestid=164819, PMID: 11470799; 6) Mouse anti-GAD67, https://www.sigmaaldrich.cn/CN/en/product/mm/mab5406, PMID: 21458543.

Animals and other research organisms

Policy information about [studies involving animals](); [ARRIVE guidelines]() recommended for reporting animal research, and [Sex and Gender in Research]()

Laboratory animals	C57BL/6J background ChAT-IRES-Cre mice were purchased from the Jackson Laboratory (stock no. 006410). C57BL/6J mice were purchased from the animal facility of Xuzhou Medical University. The mice were group housed (no more than 4 per cage) on a 12-hour light/dark cycle in an environment with stable temperature (21-23 ℃) and humidity (40-70%). They have ad libitum access to water and food. Male C57BL/6J and heterozygous transgenic mice at least 8 weeks old were used for the experiments. All behavioural experiments were performed during the light cycle. Efforts were made to minimize animal suffering and to reduce the number of mice used.
Wild animals	We did not use wild animals in this study.
Reporting on sex	We used only male mice in our study because pain modalities and methods to establish pain models in this study have been commonly reported in previous studies using either male mice or female mice, or both. We state in Discussion that it is a limitation of this study.
Field-collected samples	We did not collect samples from wild field.
Ethics oversight	The care and use of animals and the experimental protocols (No. 202011A363) used in this study were approved by the Institutional Animal Care and Use Committee and the Office of Laboratory Animal Resources of Xuzhou Medical University under the Regulations for the Administration of Affairs Concerning Experimental Animals (1988) in China.

Note that full information on the approval of the study protocol must also be provided in the manuscript.
